# A Novel One-Pot and One-Step Microwave-Assisted Cyclization-Methylation Reaction of Amino Alcohols and Acetylated Derivatives with Dimethyl Carbonate and TBAC

**DOI:** 10.1155/2014/634935

**Published:** 2014-10-14

**Authors:** Adrián Ochoa-Terán, Leticia Guerrero, Ignacio A. Rivero

**Affiliations:** ^1^Centro de Graduados e Investigación en Química del Instituto Tecnológico de Tijuana, Boulevard Alberto Limón Padilla S/N 22510 Tijuana, BC, Mexico; ^2^Área de Ciencias Básicas e Ingenierías, Universidad Autónoma de Nayarit, Cd. de la Cultura Amado Nervo S/N 63190 Tepic, Nay, Mexico

## Abstract

A simple and efficient microwave-assisted methodology for the synthesis of 4-substituted-3-methyl-1,3-oxazolidin-2-ones from amino alcohols catalyzed by a ionic liquid was developed. This novel one-pot and one-step cyclization-methylation reaction represents an easier and faster method than any other reported protocols that can be used to obtain the desired products in good yields and high purity. Applying microwave irradiation at 130°C in the presence of TBAC, dimethyl carbonate acts simultaneously as carbonylating and methylating agent and surprisingly promotes an *in situ* basic trans esterification when a *N*-acetylated amino alcohol is used as starting material. Furthermore, dimethyl carbonate worked better than diethyl carbonate in performing this reaction.

## 1. Introduction

In general, amino alcohols are readily available by reduction of an *α*-aminoacid and are important precursors in the synthesis of 1,3-oxazolidin-2-ones [1–6]. Phenylalanine-derived aminoalcohols, phenylglycine, and valine, among others, are used for the preparation of commercially important 1,3-oxazolidin-2-one chiral auxiliaries. In this context, a large number of reagents have been used in the presence of amino alcohols to synthesize 1,3-oxazolidin-2-ones. Early methods are focused to employ phosgene in reactions with amino alcohols [7–9]. Other early efforts toward the synthesis of 1,3-oxazolidin-2-ones consist in the fusion of urea with the amino alcohols above their melting points [10–12]. Cyclization with tosyl chloride can be achieved with* N*-methylated Boc amino alcohol derivatives [13]. More recently, with the decreasing use of phosgene, an efficient synthesis of 1,3-oxazolidin-2-ones from diethyl carbonate (DEC) has become prevalent [3, 14, 15].

**Scheme 1:**
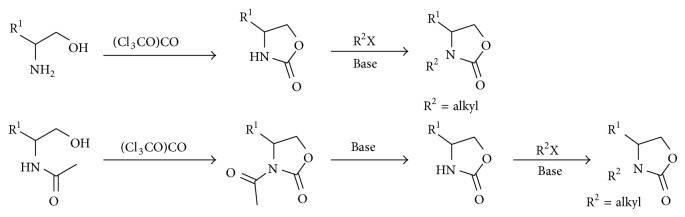
Conventional procedures in the synthesis of 4-substituted* N*-alkyl-1,3-oxazolidin-2-ones.

In general, the preparation of 4-substituted-*N*-alkyloxazolidin-2-ones is achieved through a two steps sequence, that is, the carbonylation of 1,2-amino alcohols or* N*-acetylated amino alcohols with phosgene or triphosgene, followed by the* N*-alkylation of the resulting 1,3-oxazolidin-2-one with an alkyl halide (R^2^X) or a basic deacetylation and alkylation (Scheme 1).

These procedures are not ecofriendly due to risks associated with toxic and corrosive reagents, such as phosgene and carcinogenic alkyl halides. A safer, easier, and ecofriendly alternative can be conceived with the use of nontoxic dimethyl carbonate (DMC). There are only a few examples where dimethyl carbonate simultaneously acts as carbonylating and alkylating agent. Among them, the case of oximes with an *α*–CH_2_ group for the synthesis of 3-methyl-4,5-disubstituted-4-oxazolin-2-ones [16]. More recently, the preparation of* N*-methylbenzoxazol-2-ones from* o*-aminophenol and dimethyl carbonate with catalytic Pb(AcO)_2_ or from* o*-aminophenol and dialkyl carbonates with catalytic K_2_CO_3_ has been reported [17, 18].

Recently, significant progress has been made in the application of ionic liquids as new kind of green solvents. Their unique chemical and physical properties have attracted their use as organic catalysts and many catalytic reactions proceeded in ionic liquids with excellent performance [19–23]. Recently, the using of ionic liquids for cycloaddition of CO_2_ to epoxides to produce cyclic carbonates has been extensively studied [24, 25].

In addition, microwave irradiation has been employed for the promotion of a variety of organic transformations leading to faster and cleaner reactions compared with conventional heating [26–28]. Pawelczyk and Zaprutko reported a two-step protocol for the synthesis of* N*-alkyl 1,3-oxazolidin-2-ones under microwave irradiation [29]. First, the 1,3-oxazolidin-2-one derived from racemic 2-amino-1-propanol was prepared in 78% yield under classical conditions using DMC and sodium methoxide. Microwave-assisted reaction conditions were attempted to explore the possibility of increasing the chemical yield, however, only unsuccessful results were observed. Then, the 1,3-oxazolidin-2-one was* N*-alkylated using K_2_CO_3_, KOH and a catalytic amount of TBAB (10% molar) as a phase transfer catalyst. The product was obtained in 54% yield after irradiation for 4 min with 450 W power of microwaves. Since the use of both ionic liquid and microwaves for organic transformations is not a widely used strategy, we decided to test whether there is any advantage for this approach in the synthesis of* N*-alkyl-1,3-oxazolidin-2-ones using alkyl carbonates and TBAC under microwave irradiation.

To the best of our knowledge, it has not been previously reported any studies to examine the use of TBAC as catalyst for the synthesis of* N*-alkyl-1,3-oxazolidin-2-ones from 1,2-amino alcohols and mono and diacetylated amino alcohols and dialkyl carbonates. Herein we report an efficient and novel synthesis of 4-substituted* N*-methyloxazolidin-2-ones where DMC acts as both carbonylating and alkylating agent in the presence of TBAC. This protocol exploits an unusual simultaneous cyclization-methylation using ionic liquids catalyst and microwave irradiation. Mono- and diacetylated amino alcohols are used in the cyclization-deacetylation-methylation process giving the desired products in excellent yields.

## 2. Results and Discussion

**Scheme 2:**
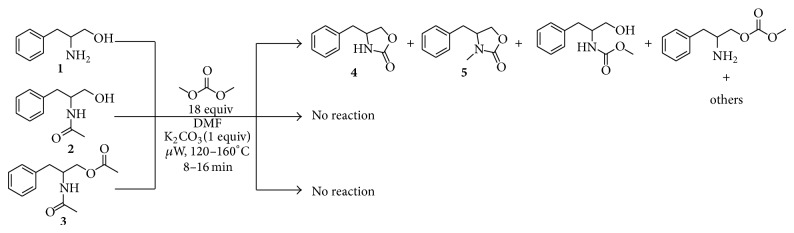
Reaction of amino alcohols** 1**,** 2**, and** 3** with DMC and K_2_CO_3 _under microwave irradiation.

Recently, our research group reported that DMC acts as both carbonylating and alkylating agent in the synthesis of 1-alkyl-2,4-quinazolinediones from 2-aminobenzamides, mediated by a ionic liquid in catalytic amount and microwaves as the energy source [30]. Based on these precedents and continuing with our research interest, we considered ionic liquids as a potential catalyst into the reaction of 1,2-aminoalcohols with DMC to form 4-substituted 3-methyl-1,3-oxazolidin-2-ones. It was also decided to work with* N*-acetylated amino alcohols in order to obtain* N*-acetylated 1,3-oxazolidin-2-ones. Initial experiments were carried out using dl-phenylalaninol (**1**),* N*-(1-Hydroxy-3-phenylpropan-2-yl)acetamine (**2**) or 2-acetamido-3-phenylpropyl acetate (**3**) with DMC (18 equivalents) and K_2_CO_3 _(1 equivalent) in* N*,*N*-dimethylformamide (DMF) and were heated under microwave irradiation at 120°C, 140°C and 160°C during 8 to 16 minutes (Scheme 2). In the analysis of crude from substrate** 1** made by GC-MS was detected a mixture of products. Considering their molecular weights, compounds** 4** (177 amu) and** 5** (191 amu) werefound to be part of the mixture into a ratio of 1 : 2 approximately. But the most abundant compounds were the carbamate and carbontate (209 amu) regioisomeric intermediates leading the synthesis of** 4** and** 5 **in a 1 : 1 ratio approximately. For the mono and diacetylated aminoalcohols (**2** and** 3**) no products were detected.

**Scheme 3:**
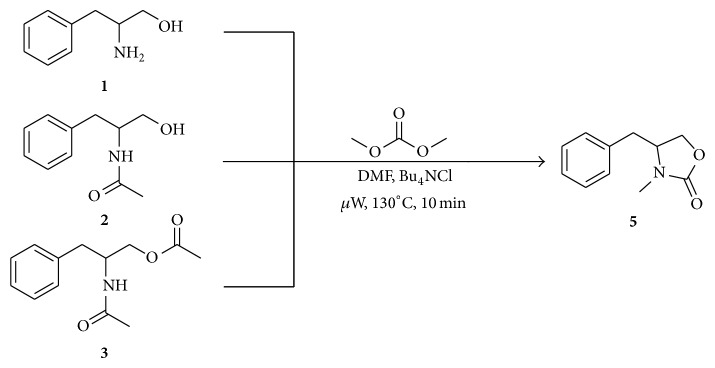
Reaction of aminoalcohols** 1**,** 2**, and** 3** with DMC and TBAC under microwave irradiation.

Experiments carried out under the same reaction conditions were also performed in the presence of TBAC instead of K_2_CO_3_ (Scheme 3). After several tests and reproducibility control, optimal conditions were determined as 130°C and 10 minutes. Surprisingly, only compound** 5** was obtained as the product in similar yields for the three substrates. Noteworthy, the amount of TBAC was decreased to 0.2 equivalents in order to determine the potential use of TBAC as an additive. Yields were similar to those obtained with one equivalent. Since good conversions were achieved, clean products were isolated after simple acidic and basic aqueus work-up without further purification.

To investigate the synthetic scope of this reaction, a variety of racemic and enantiopure amino alcohols derived from *α*-aminoacids were carbonylated and methylated using DMC, 0.2 equivalents of TBAC and DMF as the solvent of choice (Table 1). All the reactions were completed in 10 min and conversions were achieved in a 79–95% yield interval. Apparently, there is not racemization during the course of the reaction due to conservation of the optical activity in the products obtained from enantiopure amino alcohols.

The reaction proceeds with high yields using all the starting materials. TBAC and DMC perform efficiently the simultaneous cyclization-methylation in amino alcohols and cyclization-deacetylation-methylation of acetylated amino alcohols under very simple conditions. In the reaction of aminoalcohols** 6**,** 8**, and** 9** a double methylation is carried out due to the presence of the aromatic hydroxyl group in tyrosinol and the indol nitrogen present in tryptophanol.

**Scheme 4:**
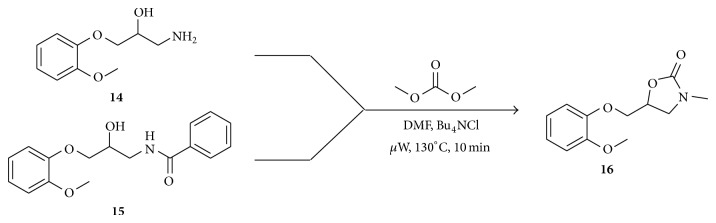
Reaction of secondary amino alcohols with DMC and TBAC.

In order to verify the protocol established using a secondary aminoalcohol, compounds** 14** and** 15** were tested under established conditions at 130°C and 10 min (Scheme 4) [31]. The reaction product observed in both reactions was the* N*-methyl mephenoxalone (**16**) showing the versatility of this method. Another important finding was that methyl benzoate was detected by GC-MS on the crude product mixture with** 15** (see Supporting Information in Supplementary Information available online at http://dx.doi.org/10.1155/2014/634935) suggesting a trans esterification in the reaction mechanism.

**Scheme 5:**
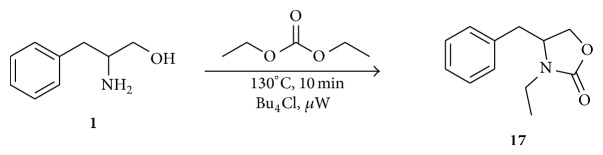
Microwave reaction of dl-phenylalaninol, DEC, and TBAC.

Finally, a similar reaction was conducted using diethyl carbonate (DEC) and dl-phenylalaninol (Scheme 5). Compound** 17** was obtained with 48% yield and purification was anfractuous. Based on these results, it was concluded that the use of DEC is less favorable than DMC for this reaction, even with the presence of TBAC in the reaction media.

In order to establish a reaction mechanism and the function of TBAC and DMF, several modified experiments were performed at 130°C, 10 min, and 200 W using aminoalcohols** 1** and** 3** as starting materials. As can be seen in Table 2 reaction does not work well using only DMC (entries 1 and 2), when TBAC was included in the reaction compound** 5** was obtained in good yields with both aminoalcohols (entries 3 and 4). At this point the needing of TBAC in the reaction was conclusive.

Then, the reaction was performed in absence of TBAC using aminoalcohol** 1** (entry 5) and several products were detected by TLC and IR spectrum showed various vibrations for carbonyl group, although the most intense was at 1749 cm^−1^ which corresponds to the carbonyl group of compound** 5**. The reaction performed with aminoalcohol** 3** works better under these conditions and compound** 5** was isolated in a 68% yield (entry 6). This result suggested that DMF or its derivate obtained by thermal degradation dimethylamine may be acting as a catalytic species.

On the other hand, it is documented that trialkylamonium salts produce trialkylamines by thermal elimination (Hofmann elimination) that may act as basic catalytic species. In order to confirm these observations some experiments were made using TEA (1.5 eq) instead TBAC. In presence of DMF the reaction of both aminoalcohols,** 1** and** 3**, was unsatisfactory (entries 7 and 8). Several compounds were detected by TLC and in the IR spectra of crude product were several vibrations for carbonyl group. In contrast, in absence of DMF the reaction was very clean and the yield of** 5** was 84% (entry 9), which is comparable with the obtained using TBAC under the same conditions. Finally, in order to explore the applicability of this method an* N*-alkylated aminoalcohol was utilized to synthesized a* N*-alkyl-1,3-oxazolidin-2-one. The reaction was performed using DMC and TBAC (0.2 eq) (entry 10) and DMC and TEA (1.5 eq) (entry 11) finding excellent results. The product was isolated with high purity from both reactions and yields were quantitative.

The last results confirmed the formation of tributylamine under conditions in which the reaction is performed. One definitive result was the detection of tributylamine and dibutylmethylamine in the reaction mixture using GC-MS. Interestingly, in the analysis of the reaction mixture performed with TEA was detected diethylmethylamine. Also, a TBAC solution was analyzed as blank sample and only tributylamine was detected demonstrating the thermal degradation of TBAC (see Supporting Information).

**Scheme 6:**
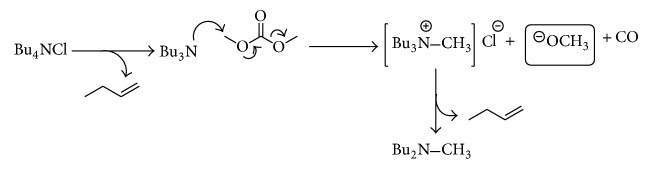
Plausible mechanism for an* in situ* generation of methoxyde ions from TBAC and DMC.

These results lead us to propose a mechanism where methoxyde ions are generated* in situ *in a two-step process. Fisrt, Hofmann elimination occurs to produce tributylamine. Then tributylamine attacks DMC forming tributylmethylammonium salt, methoxyde ions, and carbon monoxide. Then tributylammonium salt degrades to dibutylmethylamine (Scheme 6).

**Scheme 7:**
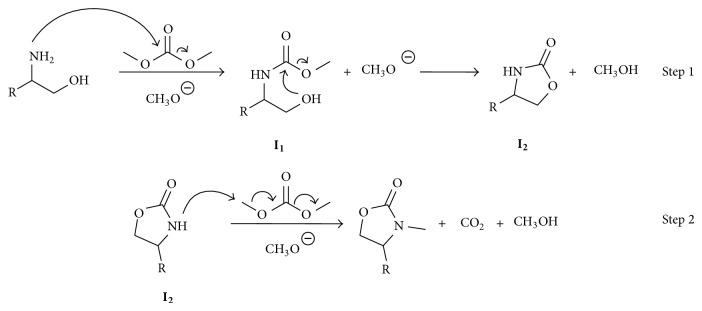
Plausible mechanism for cyclization of amino alcohols using TBAC and DMC.

It is believed that these uncommon reactions proceed through a *B*_AC⁡_2/*B*_*AL*⁡_2 sequence [18, 32, 33]. A plausible mechanism leading to 4-substituted-3-methyloxazolidin-2-ones is proposed in Scheme 7; TBAC generates a basic environment in the reaction media that favors the nucleophilic attack of the amino group to DMC yielding the intermediate** I**_**1**_ (*B*_AC⁡_2 reaction), followed by a ring closure to the oxazolidin-2-one** I**_**2 **_(Step 1). Although the intermediate** I**_**2**_ is not observed by GC-MS under these reaction conditions, the *B*_*AL*⁡_2* N*-methylation of** I**_**2**_ must yields the final product (Step 2).

**Scheme 8:**
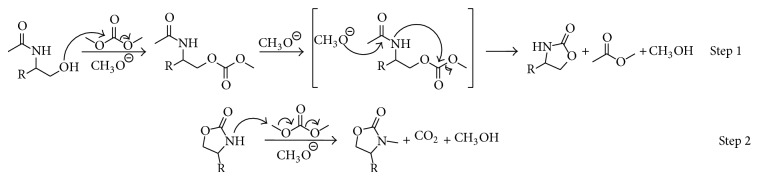
Plausible mechanism for the cyclization of* N*-acetylated aminoalcohol using TBAC and DMC.

As well, a plausible mechanism starting with a* N*-acetylated aminoalcohol is shown in Scheme 8, here hydroxyl group attacks DMC yielding an unsymmetric carbonate which could be transformed into the 1,3-oxazolidin-2-one via a concerted transition state were an* in situ* basic trans esterification occurs between the methoxyde ions released from DMC and the carbonyl of the* N*-acetylamino group, followed by the intramolecular attack of the amine to the carbonate (Step 1). Then the 1,3-oxazolidin-2-one is* N*-methylated yielding the final product (Step 2). In the reaction mechanism it is proposed that a molecule of ethyl acetate and a molecule of methanol are obtained as byproducts.

**Scheme 9:**
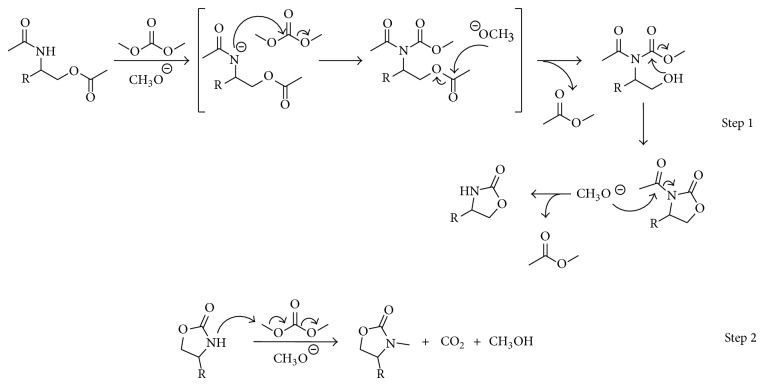
Plausible mechanism for the cyclization of adiacetylated aminoalcohol using TBAC and DMC.

In the reaction with a diacetylated aminoalcohol is proposed a transition state were the acetylamide group reacts with the DMC forming an* N*-acetylcarbamate intermediate which subsequently reacts with a methoxyde ion via a trans esterification process, producing an alcohol (Scheme 9). Then, a cyclization occurs by attack of hydroxyl group to the carbamate group forming the* N*-acetyl-1,3-oxazolidin-2-one. Finally, a transesterification process leads the 1,3-oxazolidin-2-one synthesis (Step 1). The 1,3-oxazolidin-2-one is then* N*-methylated, yielding the final product (Step 2). In the proposed reaction mechanism, two molecules of ethyl acetate and methanol are obtained as byproducts.

The proposed mechanisms are supported by the following experimental findings: (i) carbamate and carbonate intermediates were detected by GC-MS when reaction was catalyzed with K_2_CO_3_ and similar intermediates can be formed in the reaction with TBAC as is indicated in Schemes 7, 8, and 9; (ii) tributylamine and dibutylmethylamine were detected by GC-MS when TBAC was used as catalyst and diethylmethylamine when TEA was used; (iii) methyl acetate and methyl benzoate were detected by GC-MS (see Supporting Information) in the crude when a* N*-benzoylated amino alcohol and a diacetylated aminoalcohol were used as starting material, providing clear evidence of the trans esterification process and methoxide ions formation during the course of the reaction (Schemes 8 and 9); (iv) when reaction was performed using the diacetylated amino alcohol and NaCl under the same experimental conditions, only starting material was recovered, showing no degradation of DMC by chloride ions or effect of ionic strength.

## 3. Conclusions

We have developed and optimized an unusual, efficient, and clean cyclization-deacetylation-methylation protocol for the synthesis of 4-susbstituted-3-methyl-1,3-oxazolidin-2-ones from a variety of amino alcohols and DMC using TBAC and microwave irradiation. The notable advantages of this method are (a) one step protocol, (b) reproducibility, (c) fast and simple work-up of products purification, (d) low quantities of TBAC with very high yields and purity, and (e) valuable environmental and synthetic advantages are the use of nontoxic compounds and catalyst. Experimental data support our proposal about the* in situ* generation of methoxyde ions and a transesterification process when acetylated amino alcohols are used in the reaction.

## 4. Experimental

All reagents and solvents were purchased from commercial suppliers and used without further purification. ^1^H-NMR and ^13^C-NMR spectra were recorded at 200 MHz and 50 MHz, respectively, on a spectrometer in CDCl_3_ with TMS as internal standard. The microwave experiments were performed in a self-tuning single mode focused synthesizer apparatus. The instrument continuously adjusted the applied voltage to maintain the desired temperature. GC-MS analysis was obtained on a gas chromatographer coupled to a mass selective detector at 70 eV by direct insertion. Infrared (IR) spectra were recorded on a FT-IR 1600 spectrometer.

### 4.1. General Procedure for Synthesis of 4-Susbtituted-3-methyloxazolidin-2-one with DMC

A designed 10 mL pressure-rated vial was charged with aminoalcohol (0.1 g), TBAC (0.2 equiv) or TEA (1.5 equiv), DMC (1 or 2 mL) and with or wihthout DMF (1 mL). The vial was sealed and the mixture under stirring was irradiated at 130°C for 10 minutes in the microwave reactor (200 W power level). The resulting mixture was cooled to 25°C and diluted with either CH_2_Cl_2_ or EtOAc. The organic layer was washed twice with 10% aqueous citric acid, twice with saturated aqueous NaHCO_3_, and twice with H_2_O. The organic layer was dried over Na_2_SO_4_, filtered, and concentrated under vacuum to afford the corresponding product.

*4-Benzyl-3-ethyloxazolidin-2-one (* ***5***). Yellow oil. 79% yield from** 1** and** 2**; 82% yield from** 3**. FTIR (NaCl): 2917, 1747, 1433, 1031 cm^−1^. ^1^H NMR (CDCl_3_, 200 MHz): *δ* 7.37–7.29 (m, 5H, Ar–H), 4.24–4.11 (m, 1H, CH), 4.01–3.87 (m, 2H, O–CH_2_), 3.12 (dd, 1H, *J* = 13.6, 4.4 Hz, Ar–CH_2_), 2.88 (s, 3H, N–CH_3_), 2.75–2.64 (m, 1H, Ar–CH_2_). ^13^C NMR (CDCl_3_, 50 MHz): *δ* 158.4, 135.4, 129.0, 128.9, 127.1, 66.6, 58.3, 38.4, 29.5.

*(R)-4-(4-methoxybenzyl)-3-methyloxazolidin-2-one (* ***10***). Colorless oil. 81.8% yield. [*α*]_D_^25^ = + 102.1° (CH_2_Cl_2_,* c* 0.90) FTIR (NaCl): 2921, 1751, 1513, 1249 cm^−1^. ^1^H NMR (CDCl_3_, 200 MHz): *δ* 7.09 (d, 2H, *J* = 8.4 Hz, Ar–H), 6.87 (d, 2H, *J* = 8.4 Hz, Ar–H), 4.19 (t, 1H, *J* = 8.2 Hz, CH), 4.02–3.84 (m, 2H, O–CH_2_), 3.80 (s, 3H, O–CH_3_), 3.07 (dd, 1H, *J* = 13.7, 4.4 Hz, Ar–CH_2_), 2.90 (s, 3H, N–CH_3_), 2.71–2.60 (m, 1H, Ar–CH_2_). ^13^C NMR (CDCl_3_, 50 MHz): *δ* 158.7, 130.0, 127.2, 114.3, 66.6, 58.5, 55.3, 37.5, 29.5.

*(S)-4-isobutyl-3-methyloxazolidin-2-one (* ***11***). Pale yellow oil. 79.6% yield. [*α*]_D_^25^ = +33.3° (CH_2_Cl_2_,* c* 1.21). FTIR (NaCl): 2958, 2927, 1755, 1042 cm^−1^. ^1^H NMR (CDCl_3_, 200 MHz): *δ* 4.40 (t, 1H, *J* = 8.3 Hz, O–CH_2_), 3.91 (dd, 1H, *J* = 8.3, 7.3 Hz, N–CH), 3.79–3.62 (m, 1H, O–CH_2_), 2.84 (s, 3H, N–CH_3_), 1.74–1.56 (m, 2H, CH_2_), 1.43–1.31 (m, 1H, CH), 0.98–0.92 (m, 6H, 2CH_3_). ^13^C NMR (CDCl_3_, 50 MHz): *δ* 157.6, 67.9, 56.0, 41.4, 29.2, 24.6, 23.6, 22.0.

*3-Methyl-4-((1-methyl-1H-indol-3-yl) methyl)oxazolidin-2-one (* ***12***). Light brown oil. 93% yield. FTIR (NaCl): 2920, 1748, 1042 cm^−1^. ^1^H NMR (CDCl_3_, 200 MHz): *δ* 7.54–7.49 (m, 1H, Ar–H), 7.33–7.08 (m, 3H, Ar–H), 6.86 (s, 1H, =CH), 4.21–4.08 (m, 1H, N–CH), 4.04–3.91 (m, 2H, O–CH_2_), 3.74 (s, 3H, N–CH_3_ indol), 3.25 (dd, 1H, *J* = 14.4, 3.1 Hz, –CH_2_), 2.95 (s, 3H, N–CH_3_), 2.86–2.74 (m, 1H, –CH_2_) ppm. ^13^C NMR (CDCl_3_, 50 MHz): *δ* 158.6, 136.9, 127.7, 127.1, 121.9, 119.2, 118.3, 109.5, 107.6, 67.0, 57.4, 32.7, 29.3, 27.7.

*(R)-3-methyl-4-((1-methyl-1H-indol-3-yl) methyl)oxazolidin-2-one (* ***13*** *).* Light brown oil. 95% yield. [*α*]_D_^25^ = +31.5° (CH_2_Cl_2_,* c* 0.89). FTIR (NaCl): 2920, 1748, 1042 cm^−1^. ^1^H NMR (CDCl_3_, 200 MHz): *δ* 7.54–7.49 (m, 1H, Ar–H), 7.33–7.08 (m, 3H, Ar–H), 6.86 (s, 1H, =CH), 4.21–4.08 (m, 1H, N–CH), 4.04–3.91 (m, 2H, O–CH_2_), 3.74 (s, 3H, N–CH_3_ indol), 3.25 (dd, 1H, *J* = 14.4, 3.1 Hz, –CH_2_), 2.95 (s, 3H, N–CH_3_), 2.86–2.74 (m, 1H, –CH_2_) ppm. ^13^C NMR (CDCl_3_, 50 MHz): *δ* 158.6, 136.9, 127.7, 127.1, 121.9, 119.2, 118.3, 109.5, 107.6, 67.0, 57.4, 32.7, 29.3, 27.7.

*4-Benzyl-3-ethyloxazolidin-2-one (* ***17***). Yellow oil. 48.0% yield. FTIR (NaCl): 2932, 1747, 1423, 1030 cm^−1^. ^1^H NMR (CDCl_3_, 200 MHz): *δ* 7.38–7.14 (m, 5H, Ar–H), 4.18–3.96 (m, 3H), 3.60 (qd, 1H, *J* = 14.7, 7.4 Hz), 3.24–3.07 (m, 2H), 2.74–2.61 (m, 1H), 1.20 (t, 3H, *J* = 7.2 Hz, CH_3_). ^13^C NMR (CDCl_3_, 50 MHz): *δ* 135.5, 129.0, 128.9, 127.2, 66.7, 55.7, 38.6, 36.9, 12.7.

### 4.2. Synthesis of 3-Benzyl-4-isobutyloxazolidin-2-one (**19**)

A designed 10 mL pressure-rated vial was charged with 2-(benzylamino)-4-methylpentan-1-ol (**18**) (0.1 g), TBAC (0.2 equiv) or TEA (1.5 equiv), and DMC (2 mL). The vial was sealed and the mixture under stirring was irradiated at 130°C for 10 minutes in the microwave reactor (200 W power level). The resulting mixture was cooled to 25°C and diluted with either CH_2_Cl_2_ or EtOAc. The organic layer was washed twice with 10% aqueous citric acid, twice with saturated aqueous NaHCO_3_, and twice with H_2_O. The organic layer was dried over Na_2_SO_4_, filtered, and concentrated under vacuum to afford the corresponding product. Colorless oil. >99.0% yield. FTIR (NaCl): 3027, 2957, 1746, 1415, 1252, 1061 cm^−1^. ^1^H NMR (CDCl_3_, 200 MHz): *δ* 7.32 (m, 5H, Ar–H), 4.80 (d, *J* = 15.2 Hz, 1H), 4.34 (d, *J* = 8.4 Hz, 1H), 4.07 (d, *J* = 15.2 Hz, 1H), 3.93 (dd, *J* = 8.5, 7.0 Hz, 1H), 3.55 (m, 1H), 1.80–1.20 (m, 3H), 0.90 (d, *J* = 6.2 Hz, 3H), 0.79 (d, *J* = 6.2 Hz, 3H). ^13^C NMR (CDCl_3_, 50 MHz): *δ* 158.6, 136.0, 129.0, 128.4, 128.2, 68.3, 53.0, 46.1, 41.1, 24.7, 23.8, 12.9.

### 4.3. GC-MS Analytical Conditions

Products** 5**,** 10**–**13,** and** 17** were analyzed under the following conditions: injector temp: 250°C, detector temp: 280°C, initial oven temp: 70°C, final oven temp: 240°C, oven temp rate: 9°C/min. Column SPB-5, 30 m × 0.25 mm, 0.25 *μ*m film. A 1 mL/min helium flow was used as mobile phase.

The detection of volatile components in reaction mixture was performed under the following conditions: injector temp: 200°C, detector temp: 280°C, initial oven temp: 45°C during 2 min, final oven temp: 250°C, oven temp rate: 30°C/min. Column 30 m × 0.25 mm, 0.25 *μ*m film. A 1 mL/min was used as mobile phase.

## Supplementary Material


^1^H NMR and ^13^C NMR spectra for representative compounds are available. Infrared spectra and GC-MS analytical data are provided.

## Figures and Tables

**Table 1:** Synthesis of 4-substituted-3-methyl-1,3-oxazolidin-2-ones.

	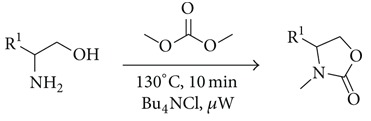		

Entry	Starting material	Product	Yield (%)^a,b^

**1**	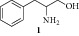	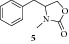	79

**2**	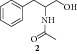	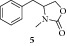	79

**3**	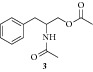	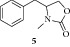	81

**4**	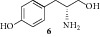	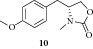	82

**5**	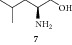	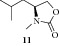	80

**6**	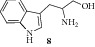	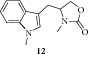	93

**7**	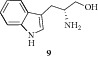	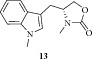	95

^a^Isolated yields based on the starting amino alcohol. ^b^Products characterized by ^1^H and ^13^C NMR.

**Table 2:** Synthesis of 4-substituted-3-alkyl-1,3-oxazolidin-2-ones under modified conditions.

Entry	Starting material	Reaction conditions 130°C, 10 min 200 W	Product	Yield (%)^a,b^
**1**	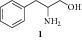	DMC	Starting material + other unidentified products	—

**2**	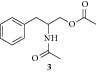	DMC	Only starting material	—

**3**	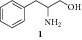	DMC, TBAC (0.2 eq)	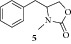	74

**4**	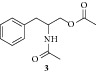	DMC, TBAC (0.2 eq)	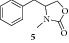	69

**5**	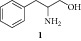	DMC, DMF	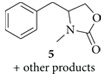	—

**6**	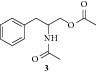	DMC, DMF	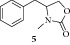	68

**7**	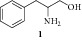	DMC, DMF, TEA (1.5 eq)	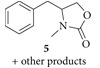	—

**8**	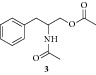	DMC, DMF, TEA (1.5 eq)	Unidentified products	—

**9**	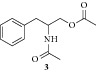	DMC, TEA (1.5 eq)	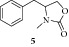	84

**10**	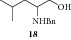	DMC, TBAC (0.2 eq)	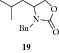	>99

**11**	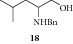	DMC, TEA (1.5 eq)	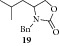	>99

^a^Isolated yields based on the starting amino alcohol. ^b^Products characterized by ^1^H and ^13^C NMR.
