# Supplementary material for: A Novel One-Pot and One-Step Microwave-Assisted Cyclization-Methylation Reaction of Amino Alcohols and Acetylated Derivatives with Dimethyl Carbonate and TBAC
Source: ScientificWorldJournal. 2014 Oct 14;2014:634935. doi: 10.1155/2014/634935 (PMC4322309; doi:10.1155/2014/634935)
Supplement: Supplementary file 1 — 1H NMR and 13C NMR spectra for representative compounds are available. Infrared spectra and GC-MS analytical data are provided. [file 634935.f1.zip › 634935.f1/634935.f1.pdf]

Supplementary Material

A novel one-pot and one-step microwave-assisted cyclization-methylation reaction of amino alcohols and acetylated derivatives with dimethyl carbonate and TBAC

Leticia Guerrero R.*[,1], Adrián Ochoa-Terán[2], Ignacio A. Rivero[2]

[1] *Área de Ciencias Básicas e Ingenierías. Universidad Autónoma de Nayarit. Cd. de la Cultura Amado Nervo S/N. Tepic, Nay. 63190. México*

[2] *Centro de Graduados e Investigación en Química. Instituto Tecnológico de Tijuana. Blvd. Alberto Limón Padilla S/N. Tijuana, B.C. 22510. México*

* e-mail: lguerrer@uan.edu.mx

S2-S17: ^{1}H NMR and ^{13}C NMR spectra

S18, S19: GC-MS and IR spectra

^{1}H-NMR and ^{13}C-NMR spectra were recorded at 200 MHz and 50 MHz respectively, on a spectrometer in $CDCl_3$ with TMS as internal standard.

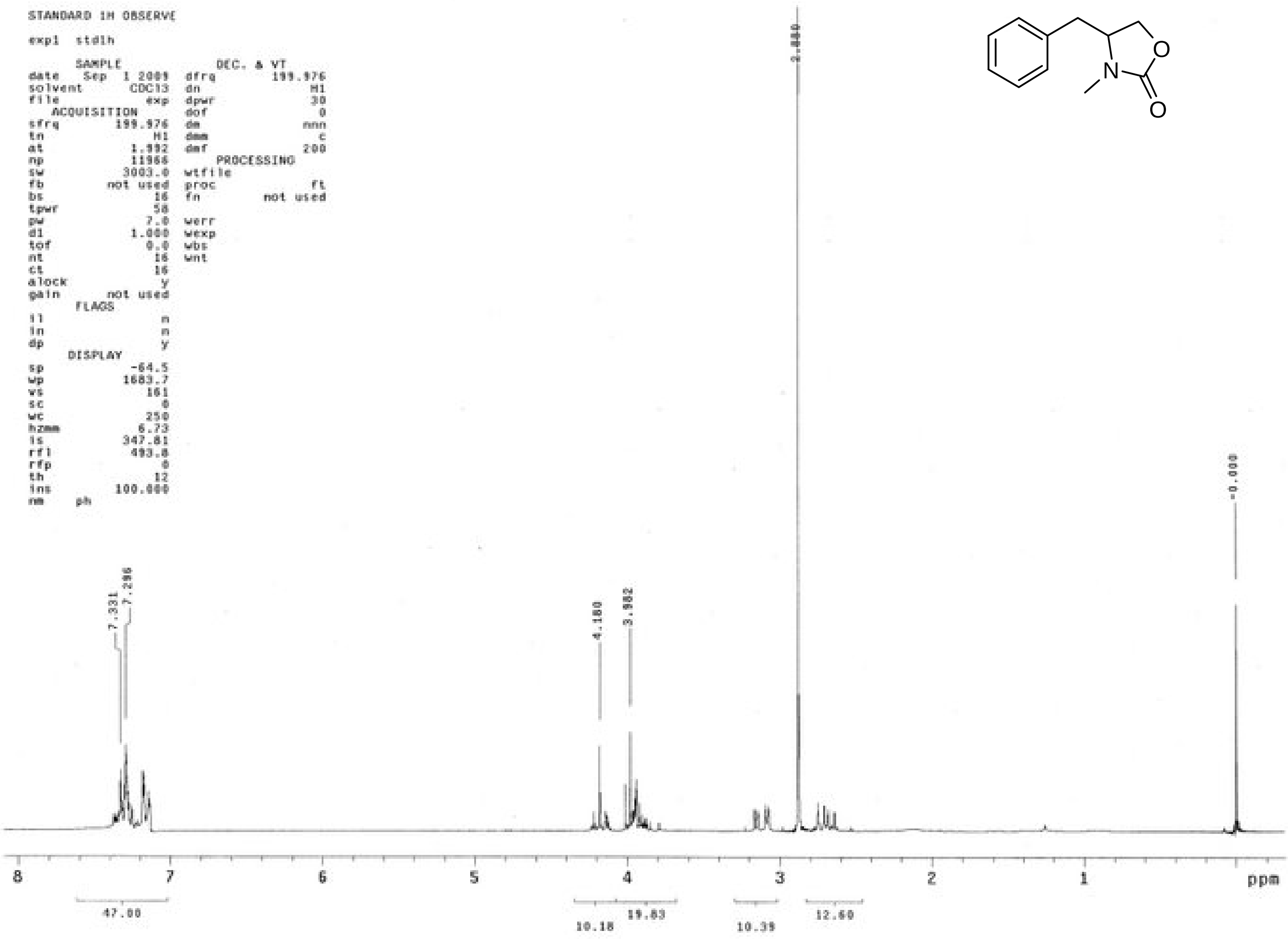

Figure 1: ^{1}H NMR spectrum of 4-benzyl-3-methyloxazolidin-2-one (**5**)

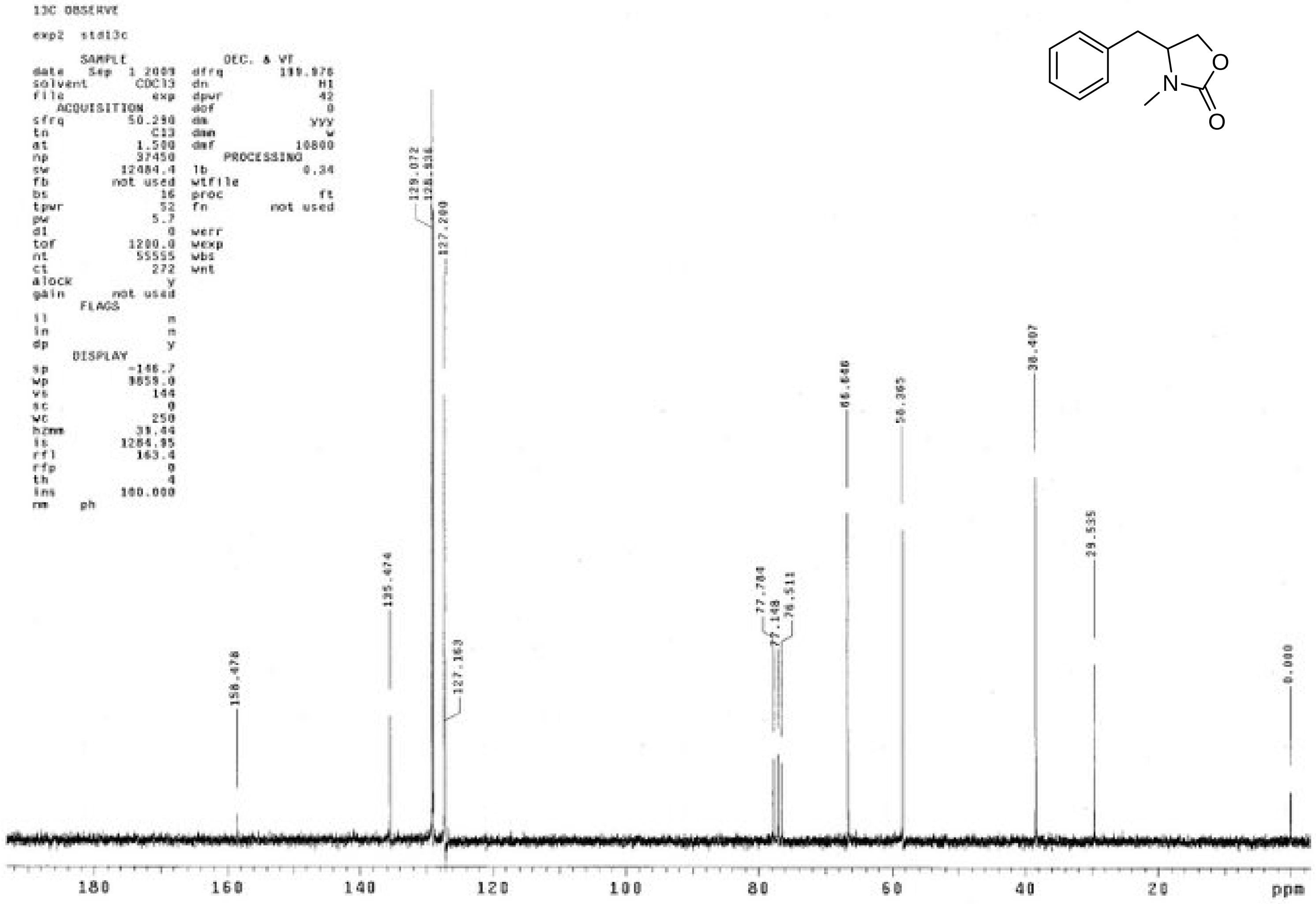

Figure 2: ^{13}C NMR spectrum of 4-benzyl-3-methyloxazolidin-2-one (**5**)

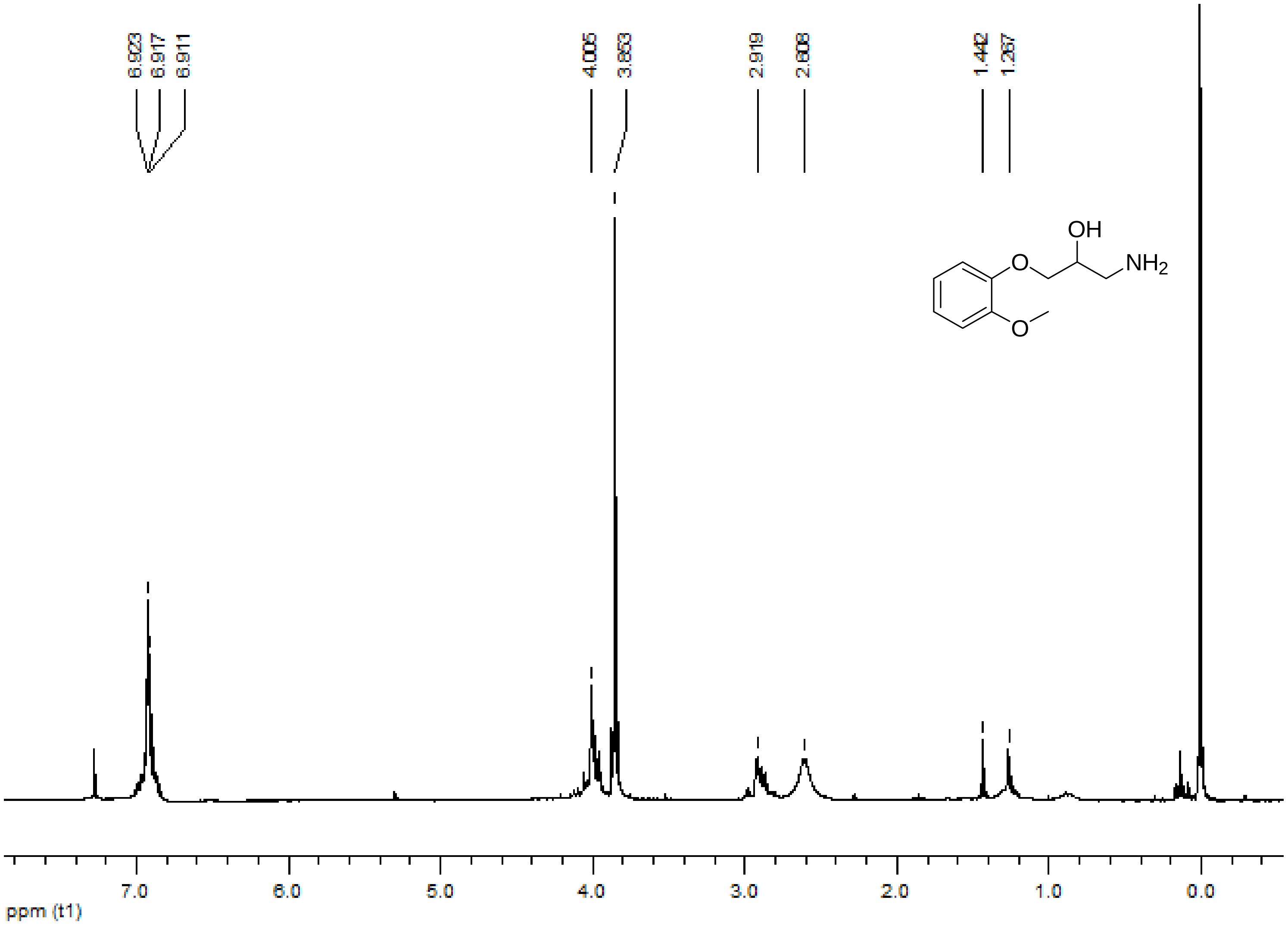

Figure 3: ^{1}H NMR spectrum of 1-amino-3-(2-methoxyphenoxy)propan-2-ol (**14**)

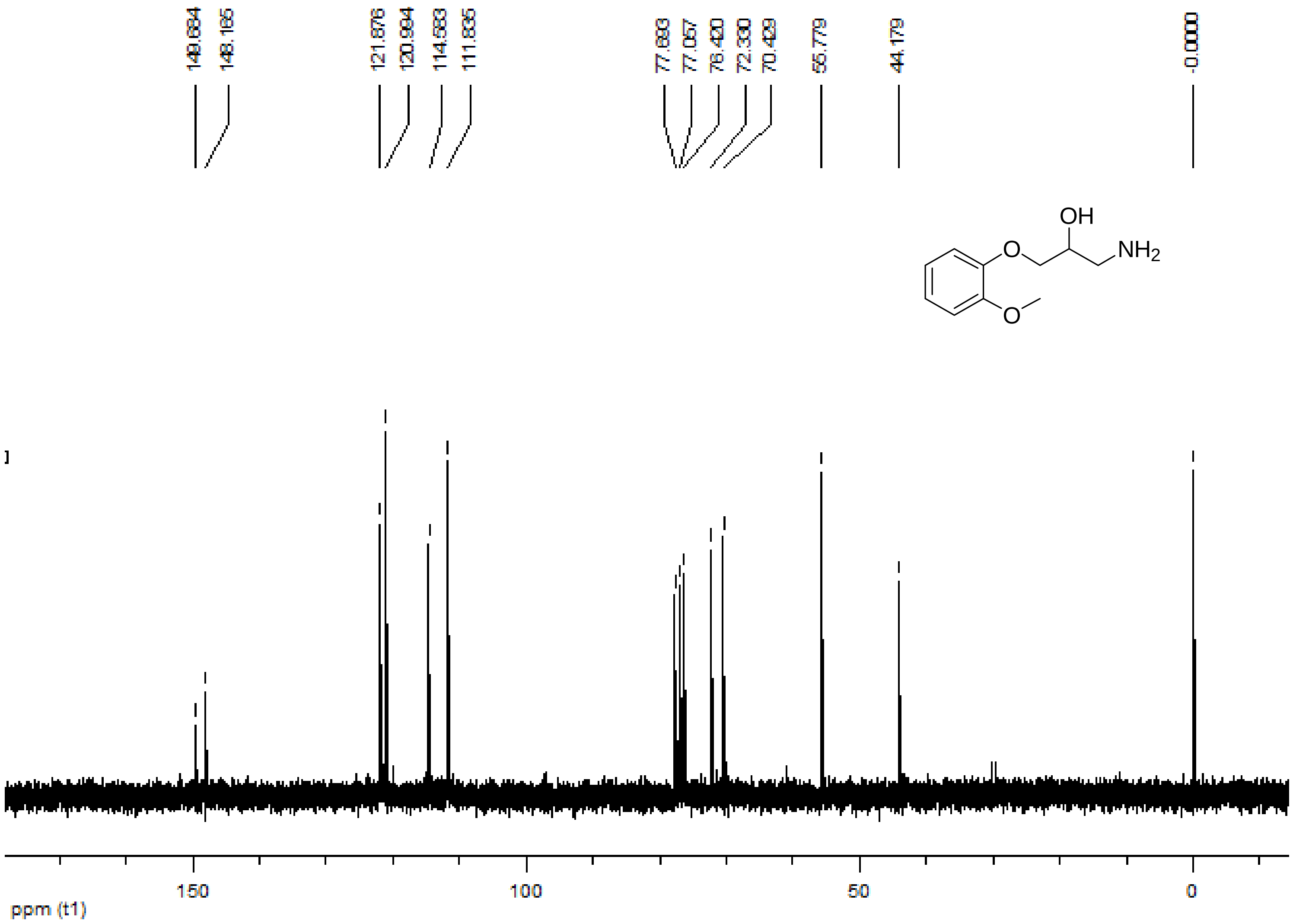

Figure 4: ^{13}C NMR spectrum of 1-amino-3-(2-methoxyphenoxy)propan-2-ol (**14**)

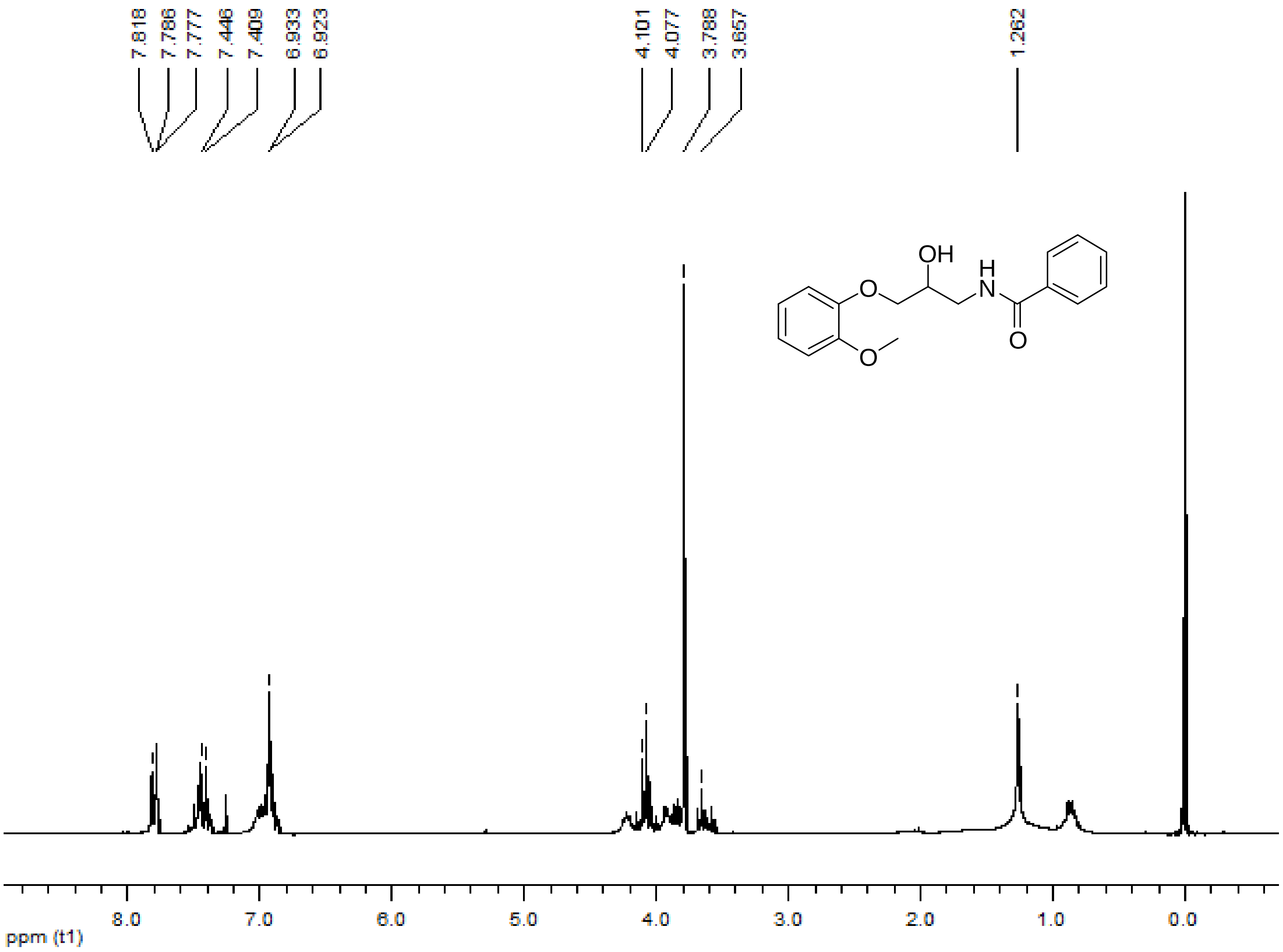

Figure 5: ^{1}H NMR spectrum of *N*-(2-hydroxy-3-(2-methoxyphenoxy)propyl)benzamide (**15**)

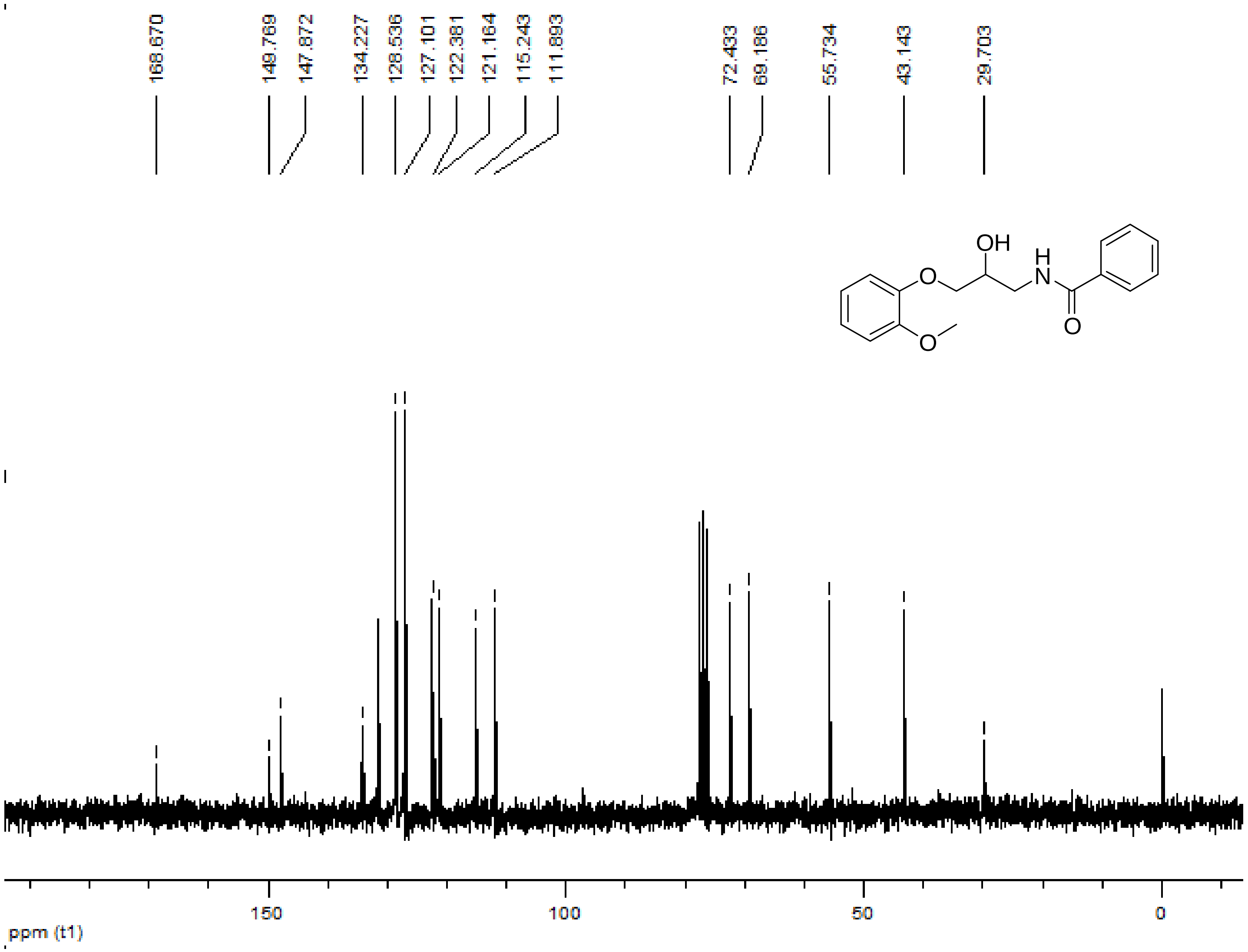

Figure 6: ^{13}C NMR spectrum of *N*-(2-hydroxy-3-(2-methoxyphenoxy)propyl)benzamide (**15**)

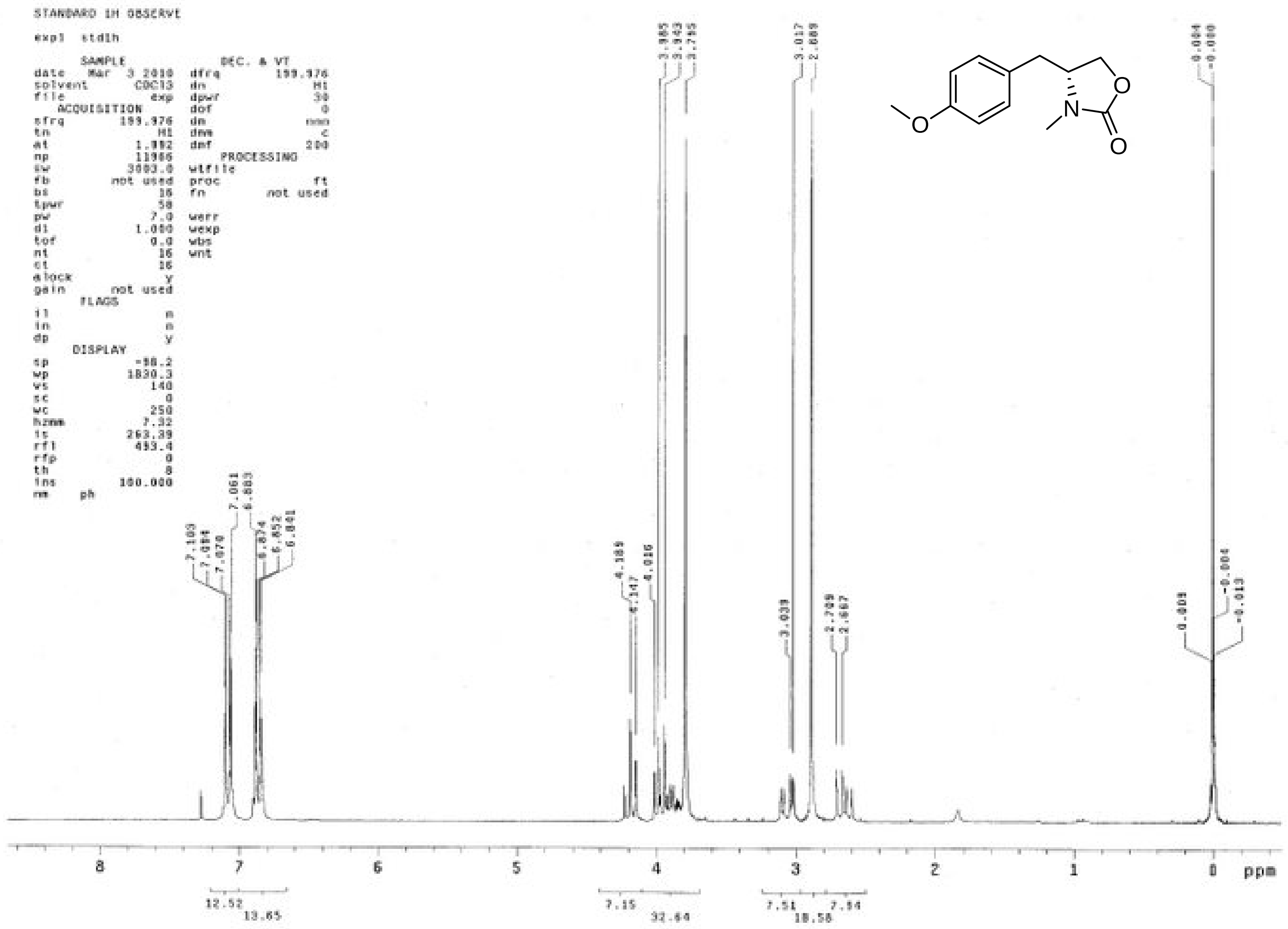

Figure 7: ^{1}H NMR spectrum of (*R*)-4-(4-methoxybenzyl)-3-methyloxazolidin-2-one (**10**)

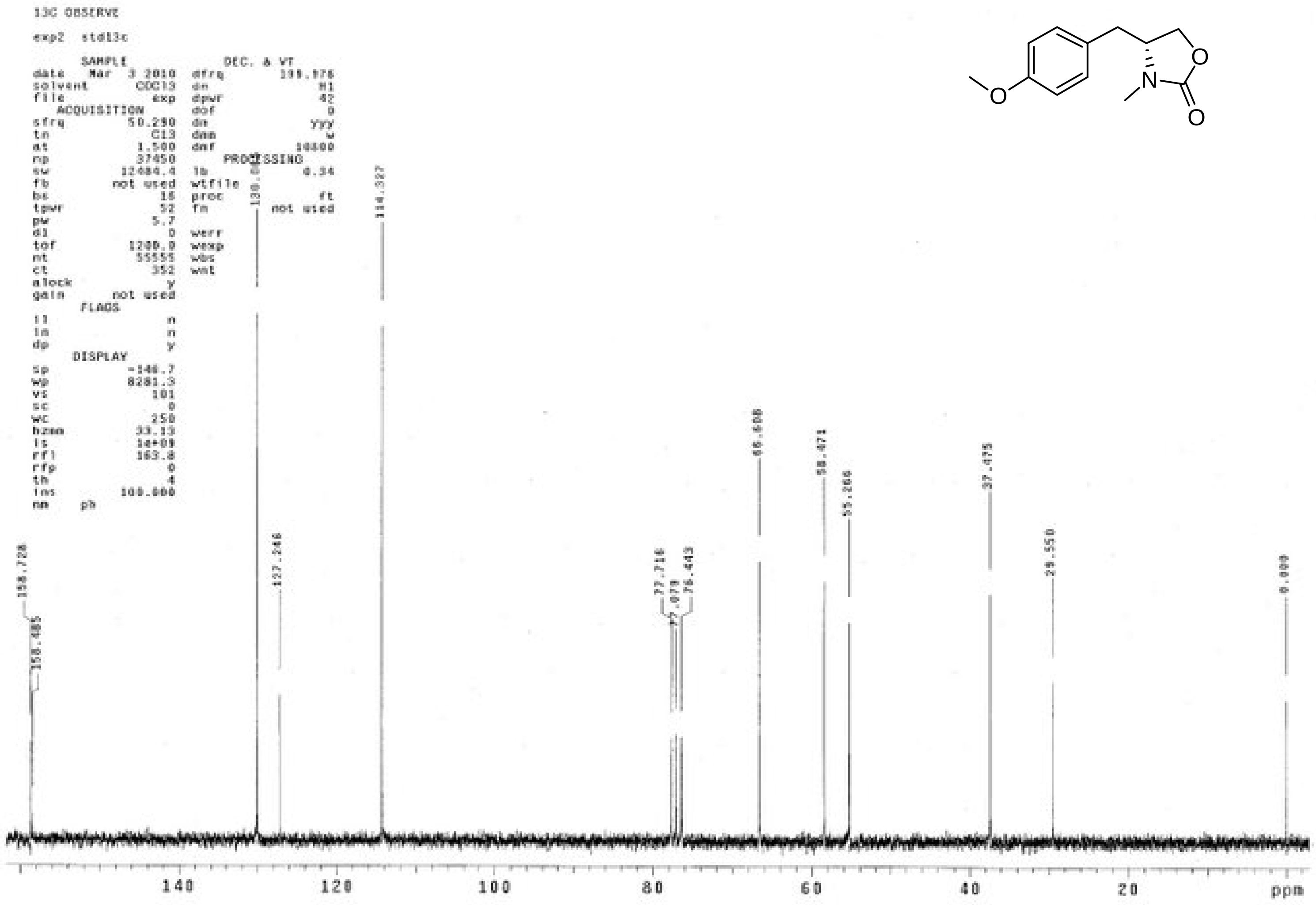

Figure 8: ^{13}C NMR spectrum of (*R*)-4-(4-methoxybenzyl)-3-methyloxazolidin-2-one (**10**)

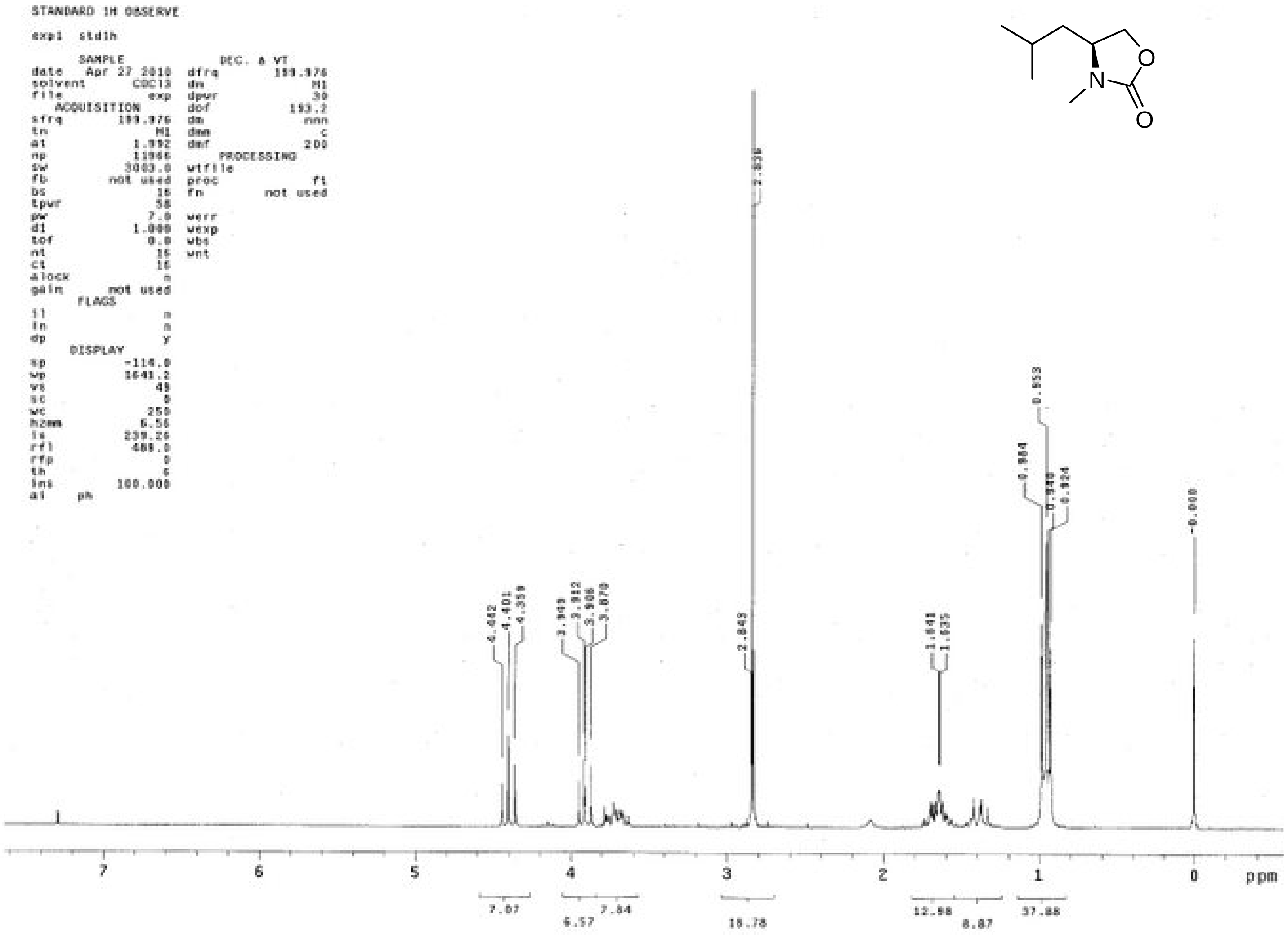

Figure 9: ^{1}H NMR spectrum of (*S*)-4-isobutyl-3-methyloxazolidin-2-one (**11**)

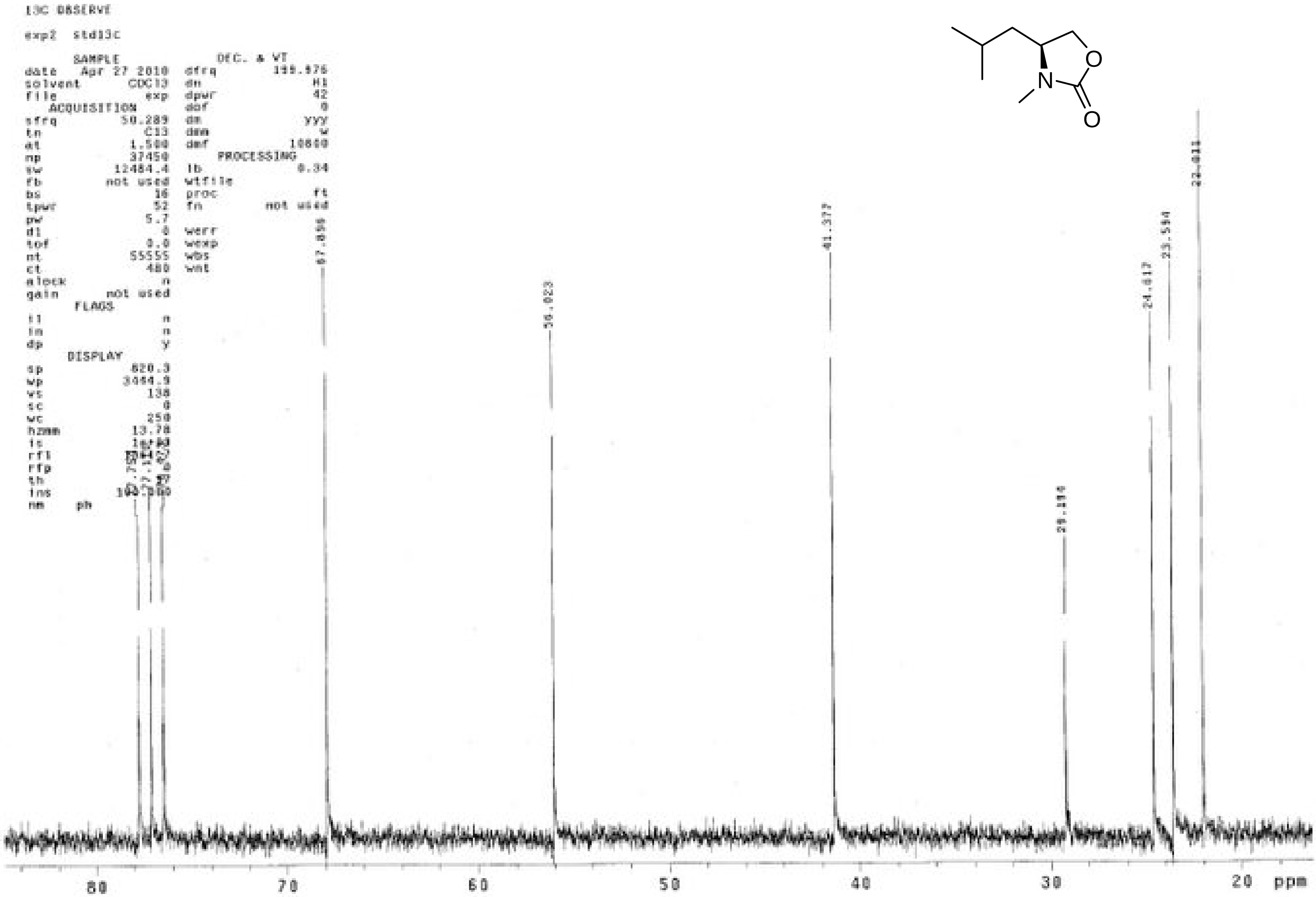

Figure 10: ^{13}C NMR spectrum of (*S*)-4-isobutyl-3-methyloxazolidin-2-one (**11**)

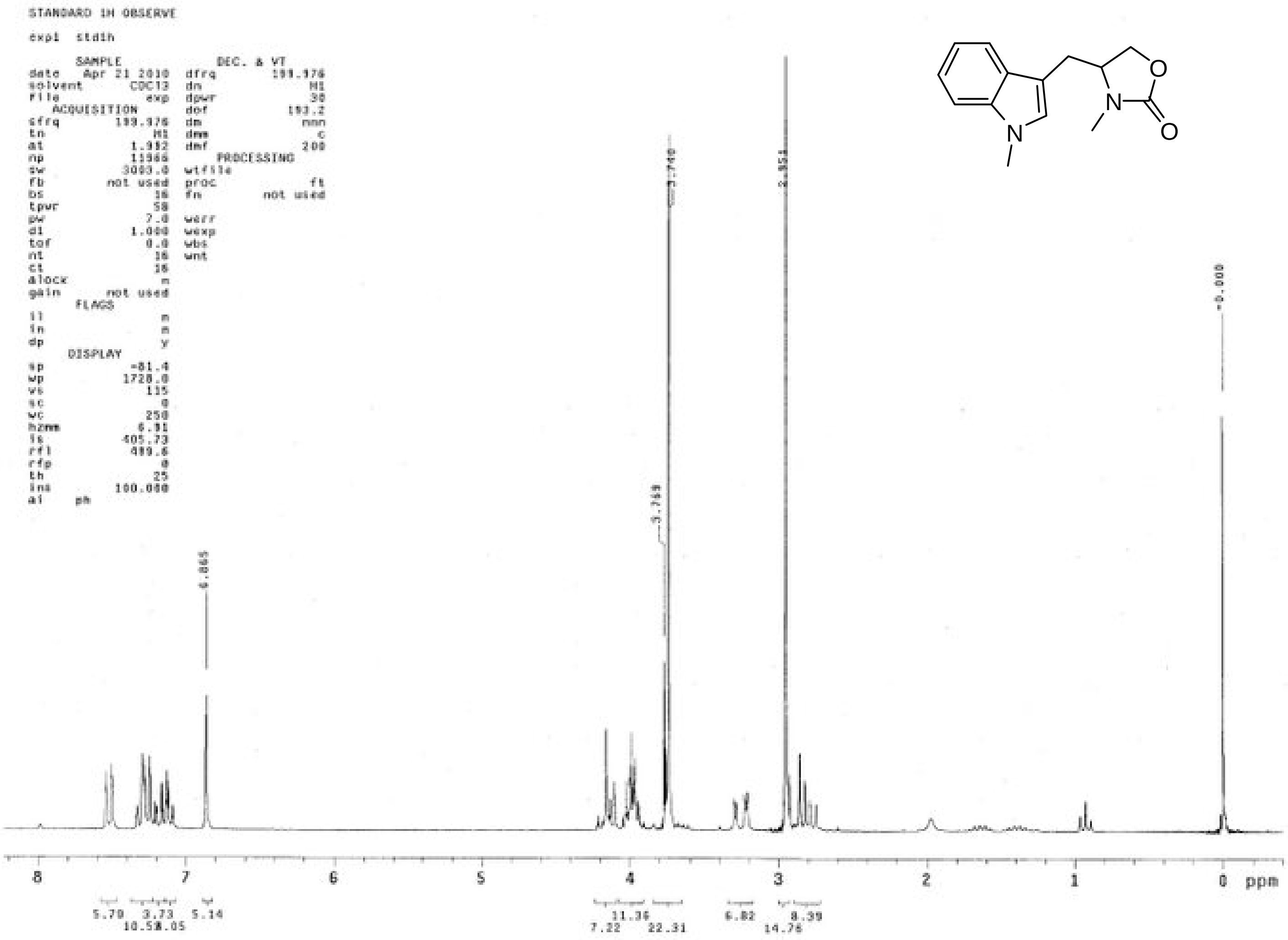

Figure 11: ^{1}H NMR spectrum of 3-methyl-4-((1-methyl-1*H*-indol-3-yl)methyl)oxazolidin-2-one (**12**)

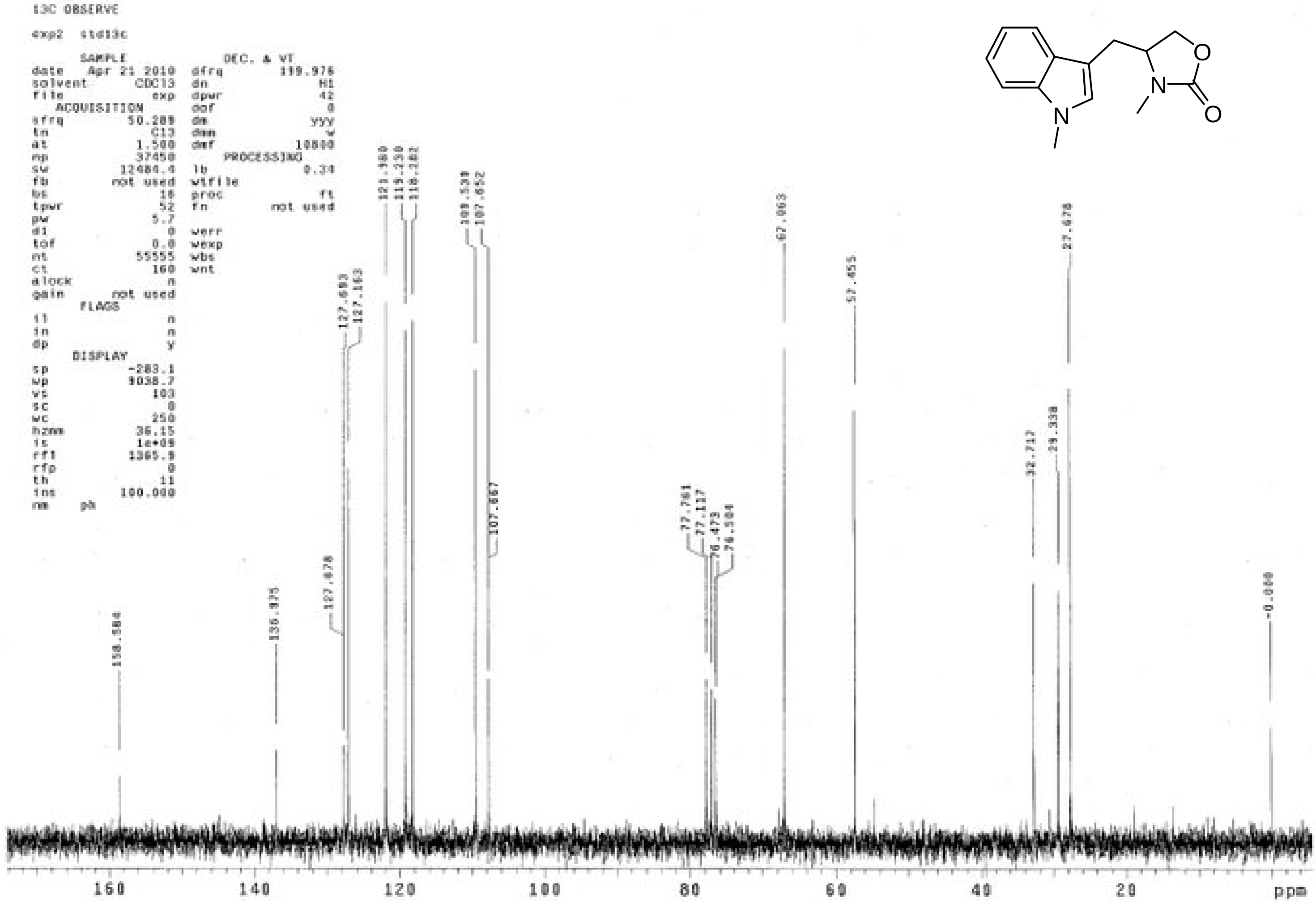

Figure 12: ^{13}C NMR spectrum of 3-methyl-4-((1-methyl-1*H*-indol-3-yl)methyl)oxazolidin-2-one (**12**)

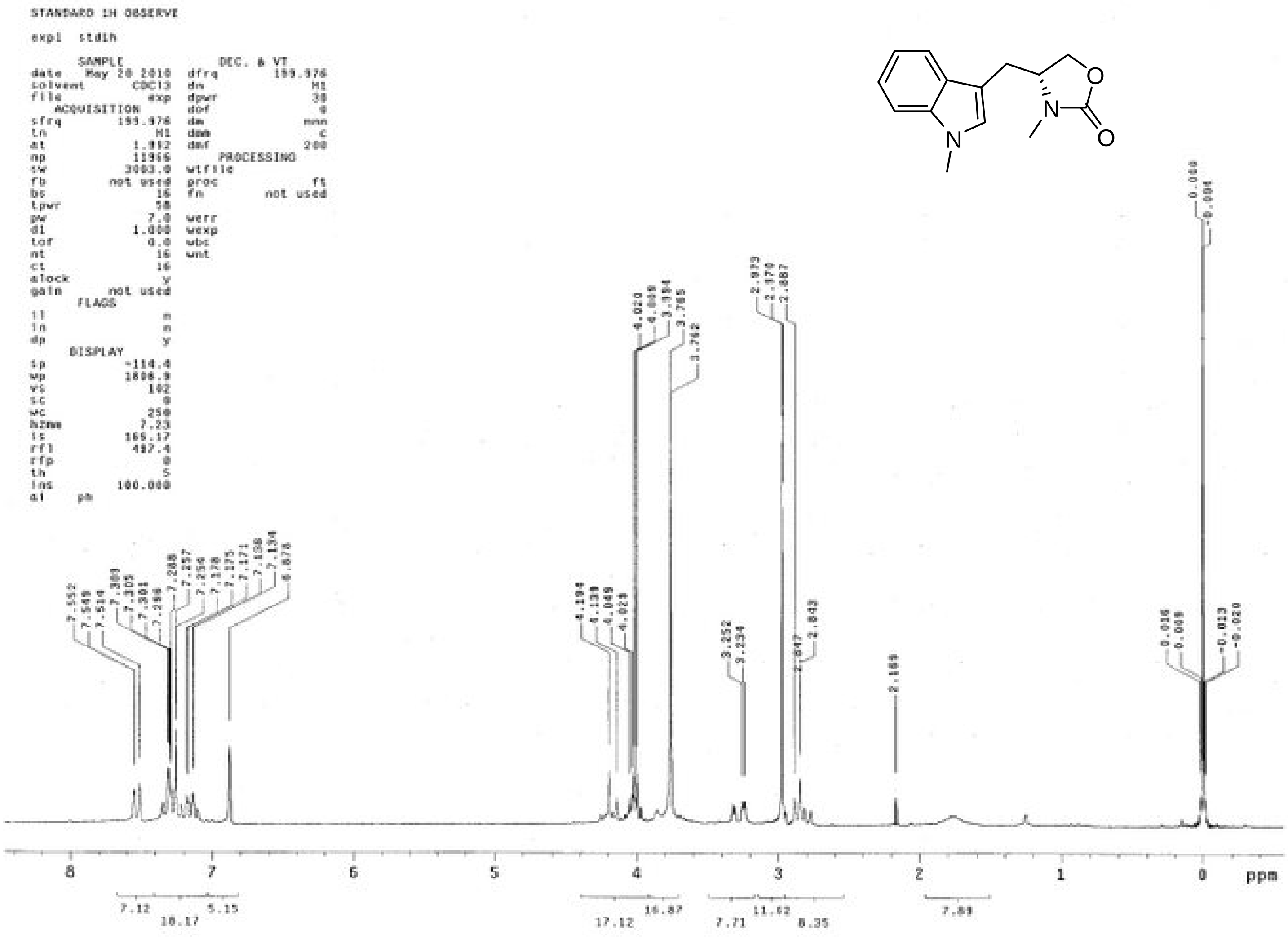

Figure 13: ^{1}H NMR spectrum of (*R*)-3-methyl-4-((1-methyl-1*H*-indol-3-yl)methyl)oxazolidin-2-one (**13**)

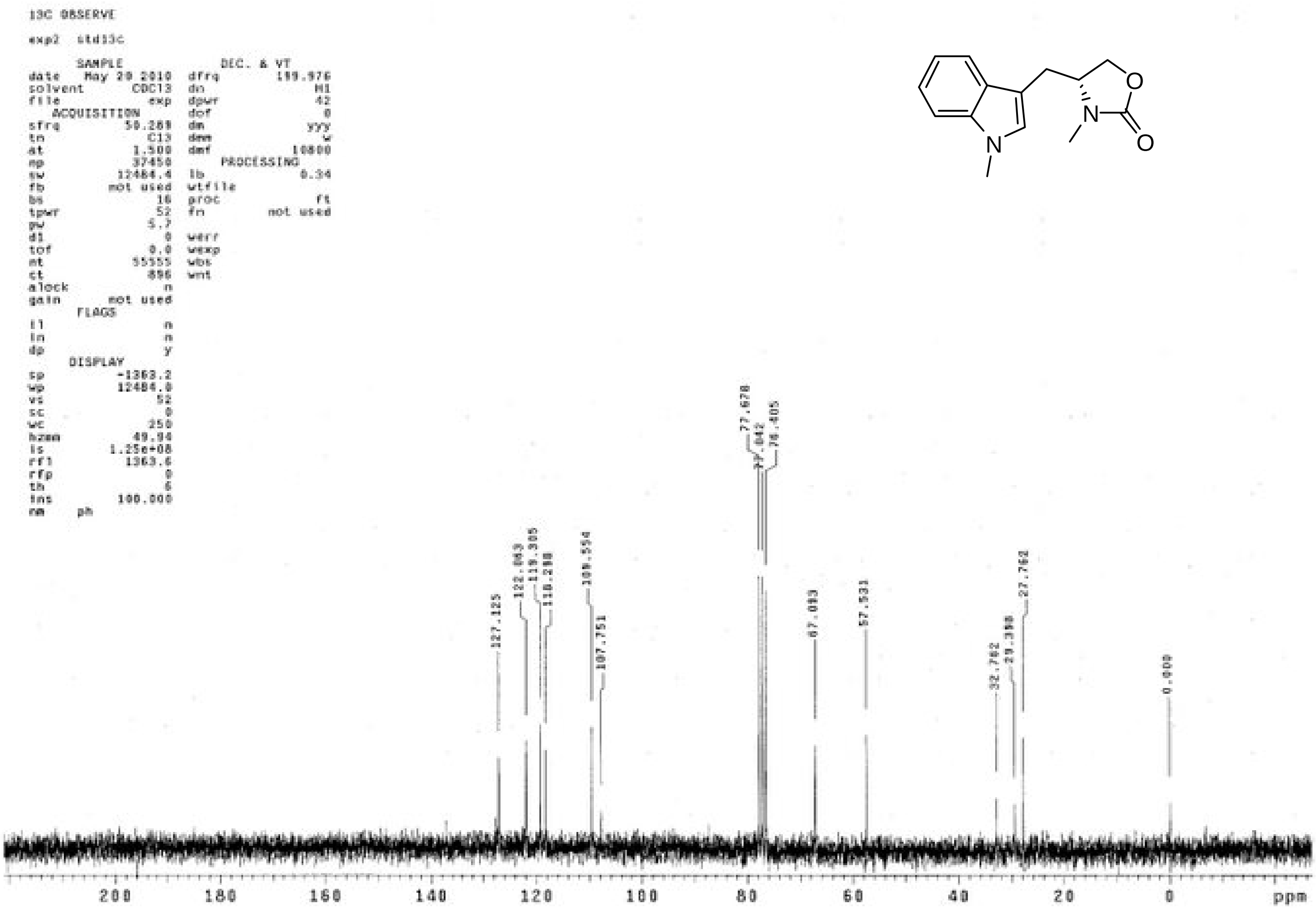

Figure 14: ^{1}C NMR spectrum of (*R*)-3-methyl-4-((1-methyl-1*H*-indol-3-yl)methyl)oxazolidin-2-one (**13**)

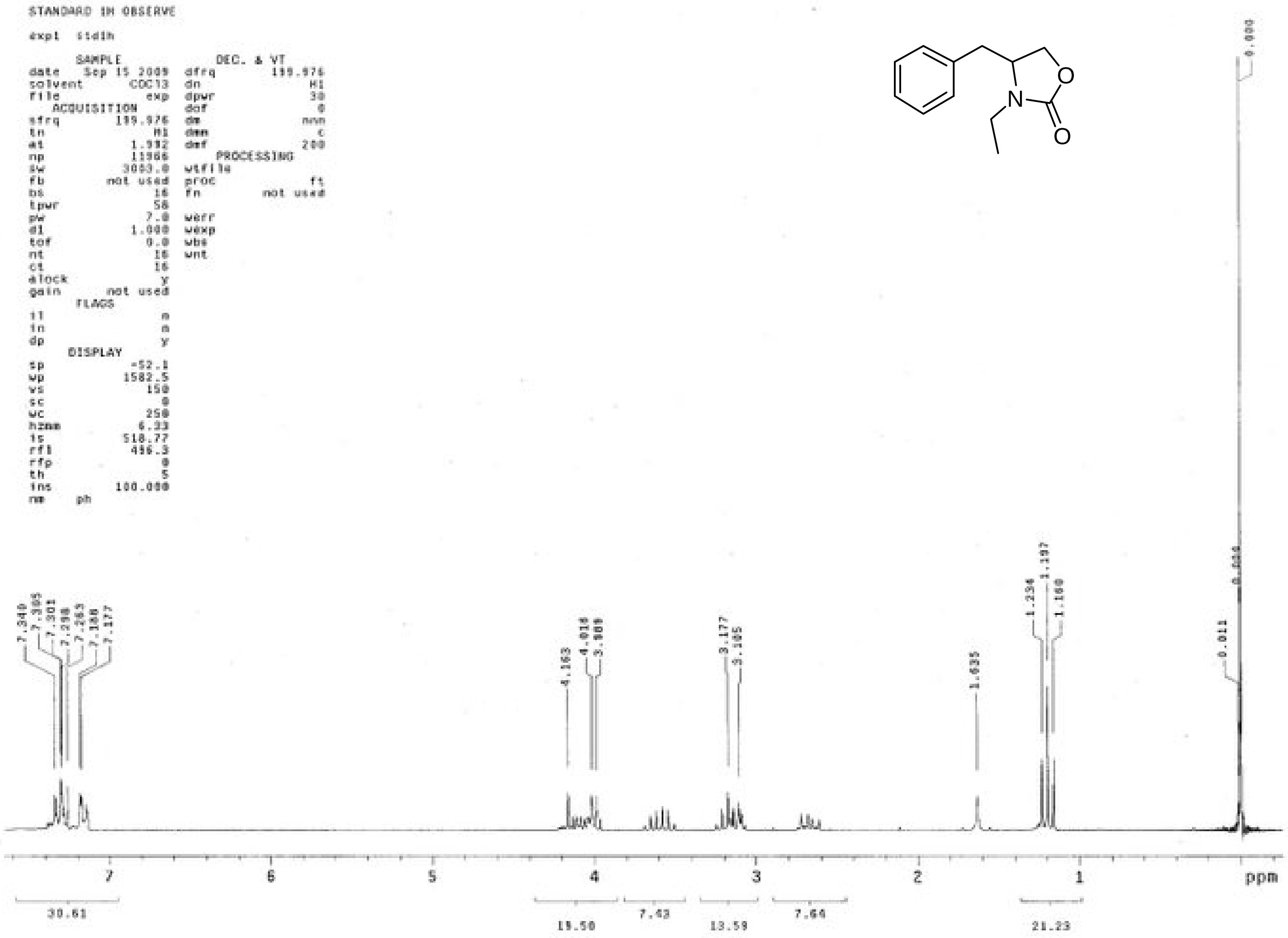

Figure 15: ^{1}H NMR spectrum of 4-benzyl-3-ethyloxazolidin-2-one (**17**)

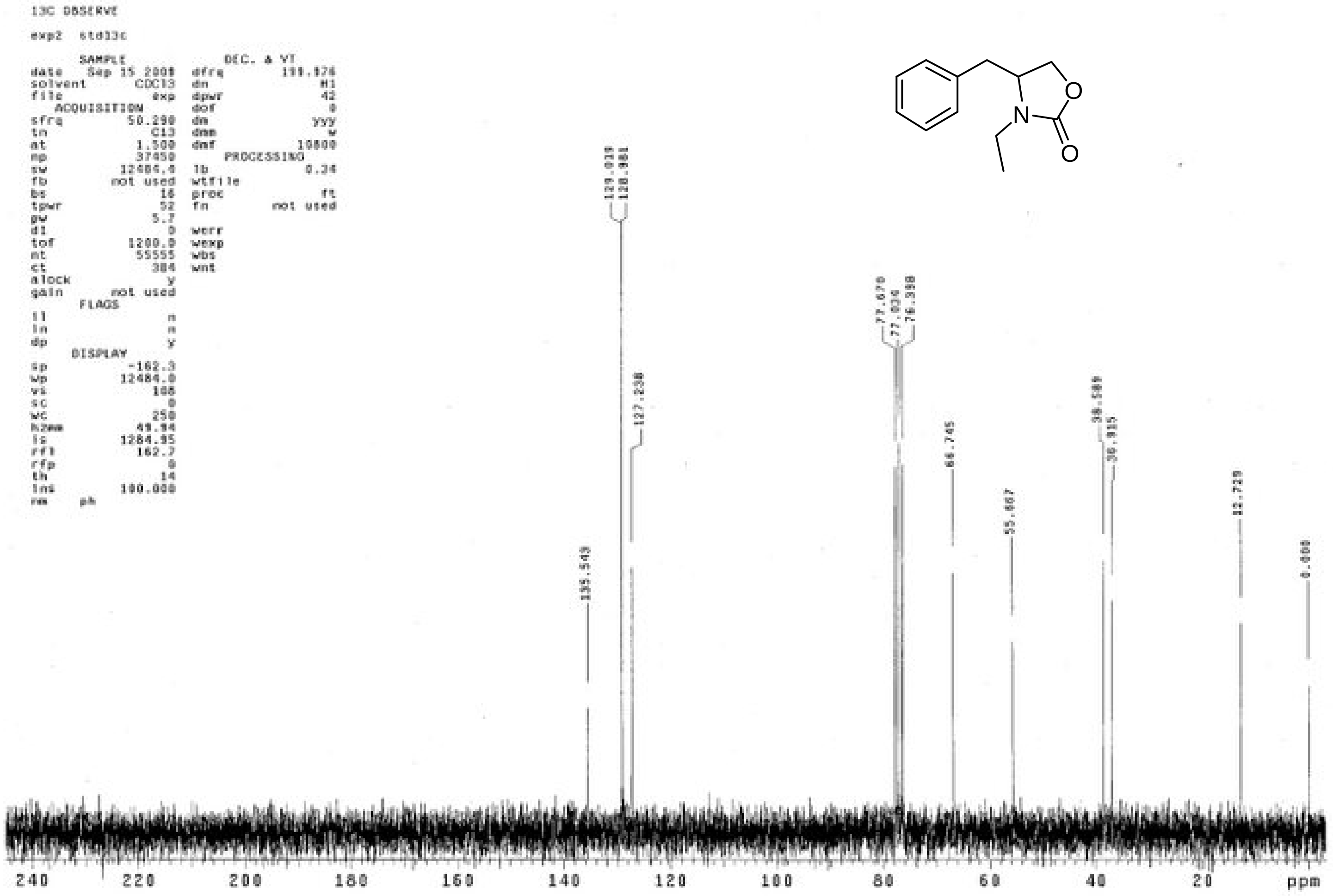

Figure 16: ^{13}C NMR spectrum of 4-benzyl-3-ethyloxazolidin-2-one (**17**)

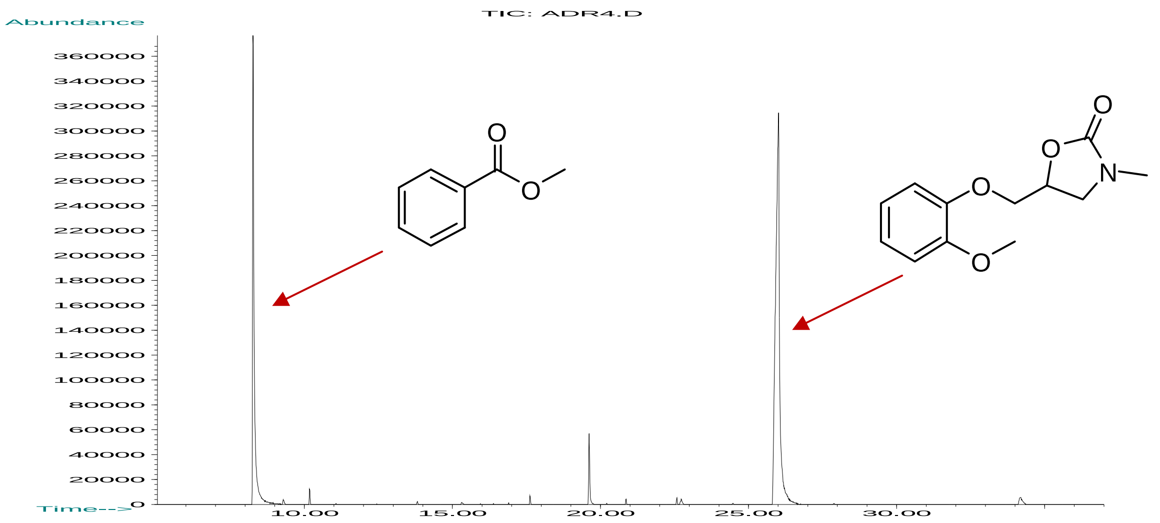

Figure 17: GC of reaction products 5-((2-methoxyphenoxy)-3-methyloxazolidin-2-one (**16**) and methyl benzoate.

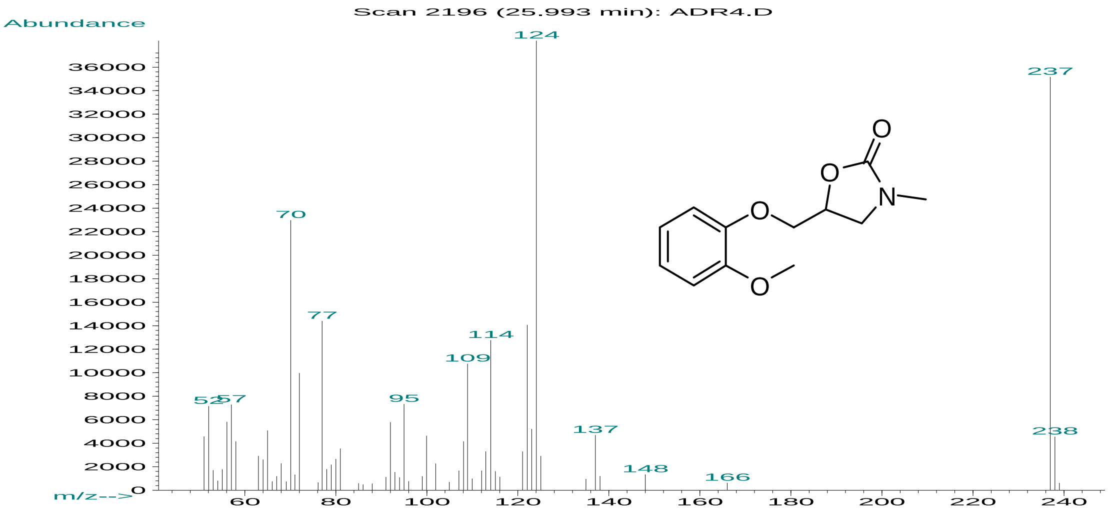

Figure 18: MS of 5-((2-methoxyphenoxy)-3-methyloxazolidin-2-one (**16**)

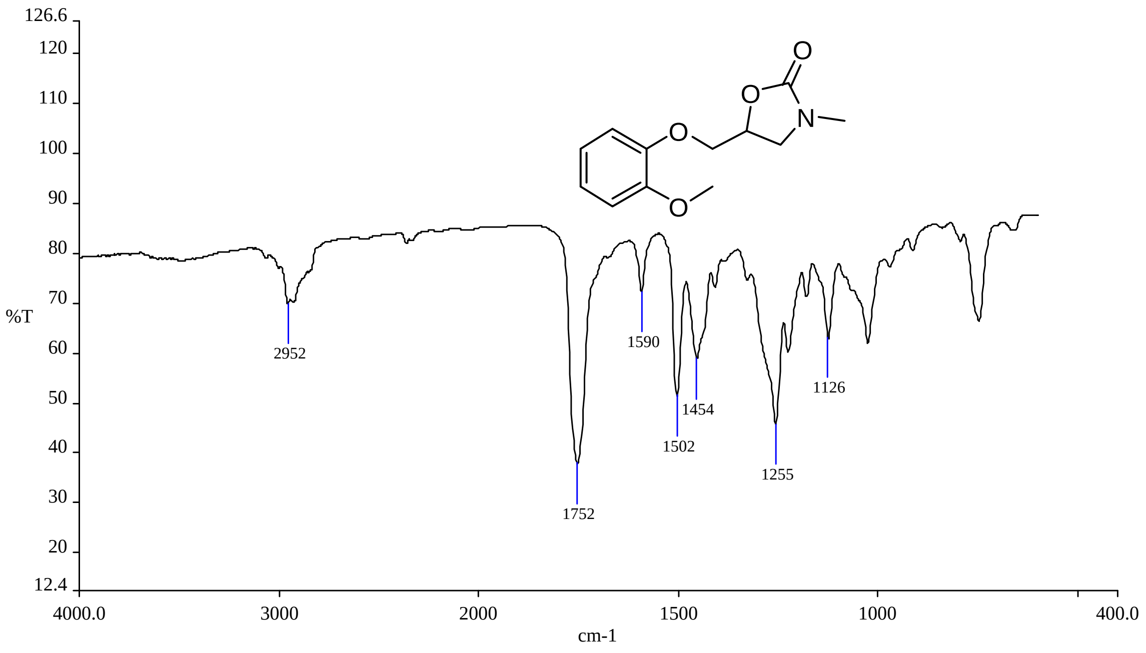

Figure 19: Infrared of 5-((2-methoxyphenoxy)-3-methyloxazolidin-2-one (**16**)
