# Supplementary material for: A Novel One-Pot and One-Step Microwave-Assisted Cyclization-Methylation Reaction of Amino Alcohols and Acetylated Derivatives with Dimethyl Carbonate and TBAC
Source: ScientificWorldJournal. 2014 Oct 14;2014:634935. doi: 10.1155/2014/634935 (PMC4322309; doi:10.1155/2014/634935)
Supplement: Supplementary file 1 — 1H NMR and 13C NMR spectra for representative compounds are available. Infrared spectra and GC-MS analytical data are provided. [file 634935.f1.zip › 634935.f1/634935.f2.pdf]

Supplementary information

A novel one-pot and one-step microwave-assisted cyclization-methylation reaction of amino alcohols and acetylated derivatives with dimethyl carbonate and TBAC

Adrián Ochoa-Terán[1], Leticia Guerrero R.*[,2] and Ignacio A. Rivero[1]

[1]*Centro de Graduados e Investigación en Química. Instituto Tecnológico de Tijuana. Blvd. Alberto Limón Padilla S/N. Tijuana, B.C. 22510. México*

[2] *Área de Ciencias Básicas e Ingenierías. Universidad Autónoma de Nayarit. Cd. de la Cultura Amado Nervo S/N. Tepic, Nay. 63190. México*

* e-mail: lguerrer@uan.edu.mx and grolet_ros@yahoo.com.mx

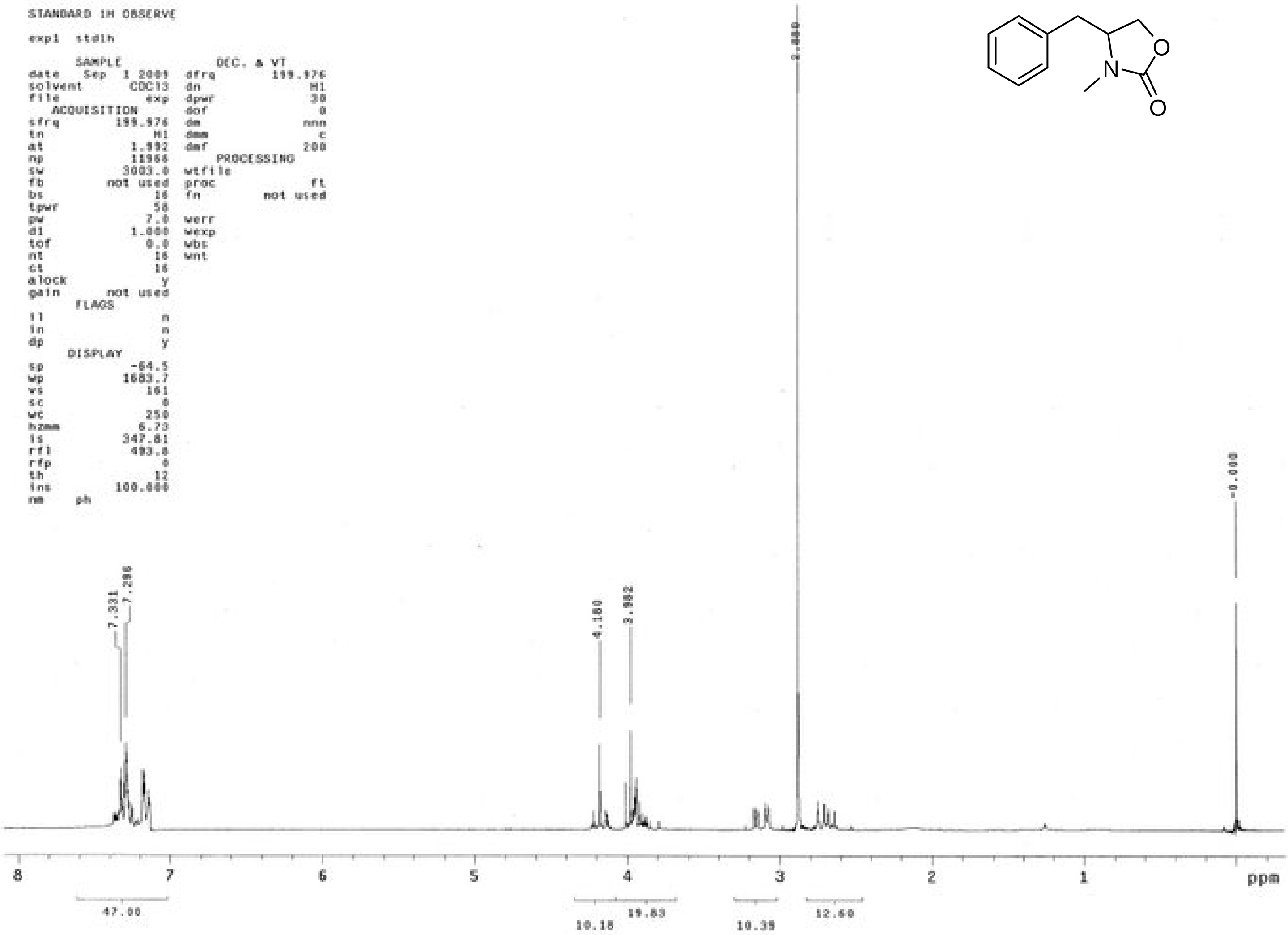

Figure 1: ^{1}H NMR spectrum of4-benzyl-3-methyloxazolidin-2-one (**5**)

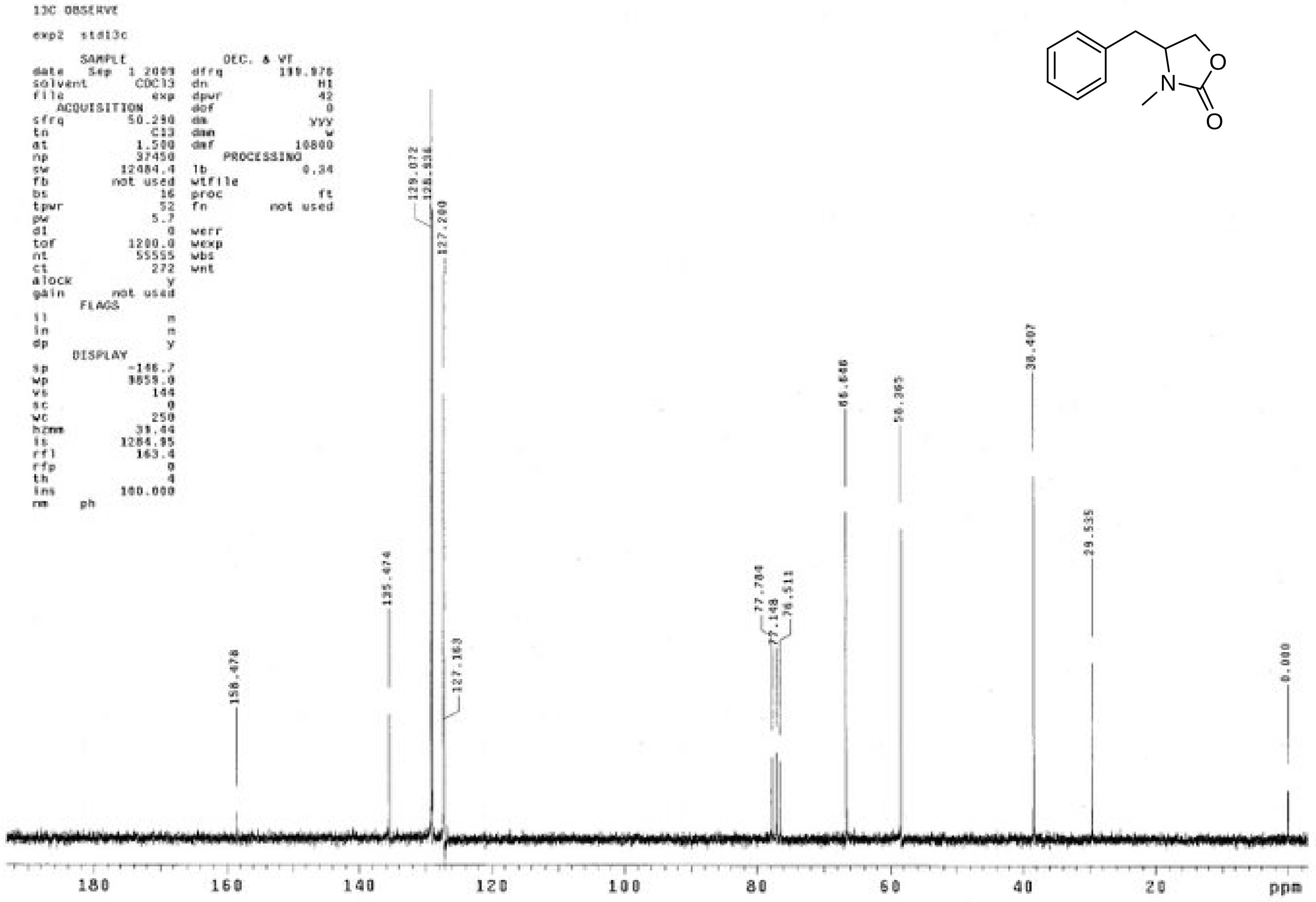

Figure 2: ^{13}C NMR spectrum of 4-benzyl-3-methyloxazolidin-2-one (**5**)

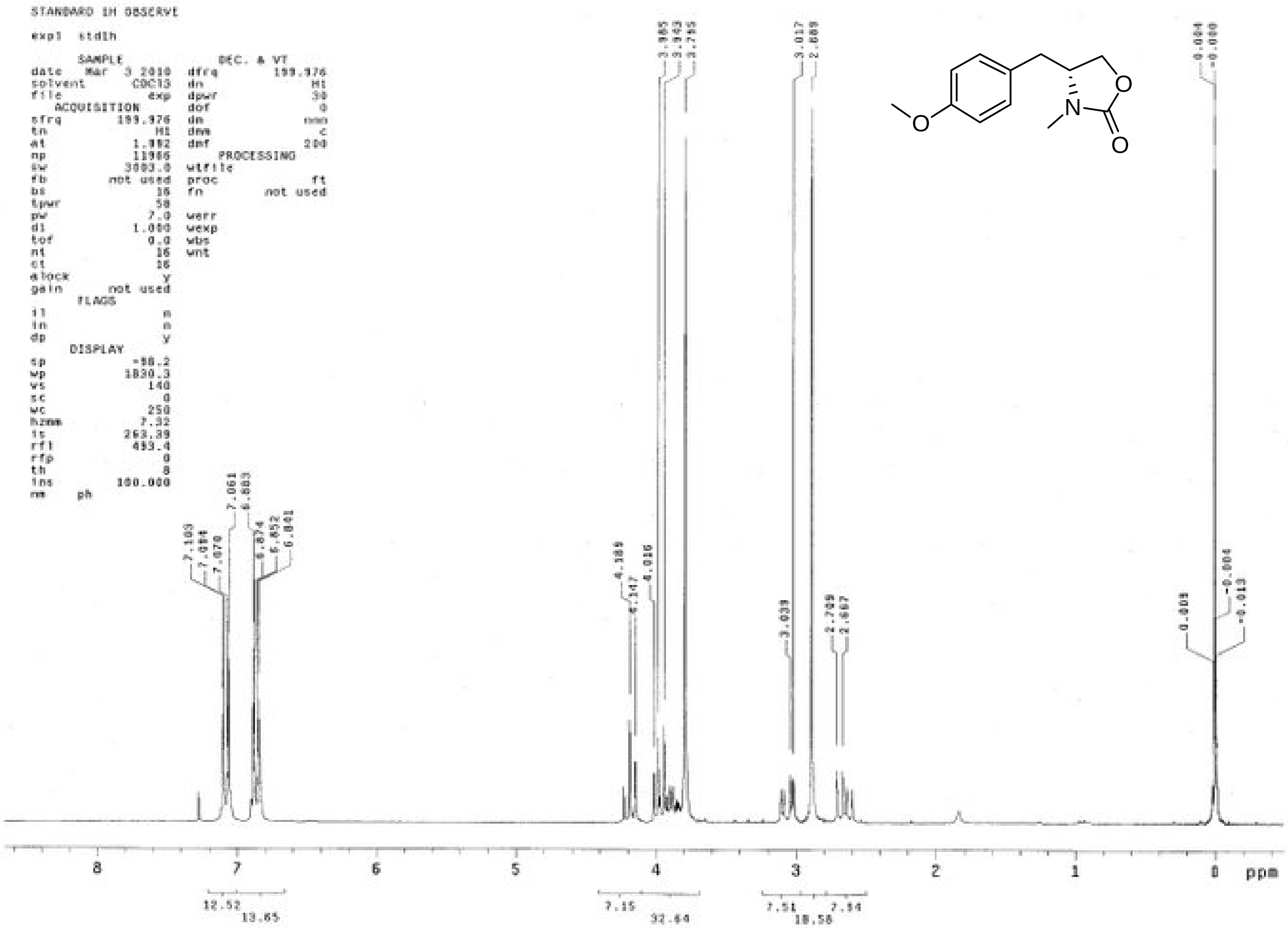

Figure 3:^{1}H NMRspectrum of (*R*)-4-(4-methoxybenzyl)-3-methyloxazolidin-2-one (**10**)

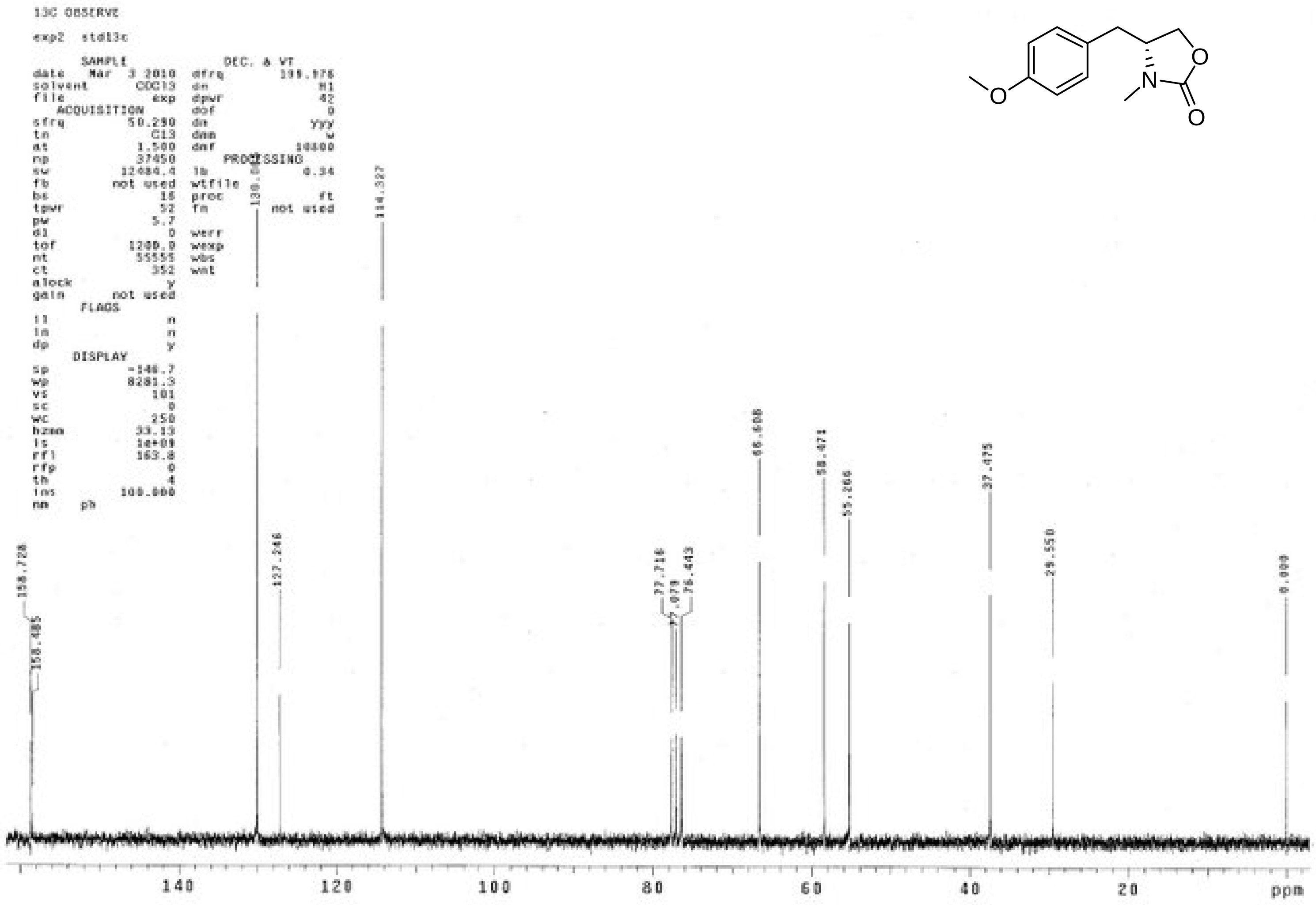

Figure 4: ^{13}C NMR spectrum of (*R*)-4-(4-methoxybenzyl)-3-methyloxazolidin-2-one (**10**)

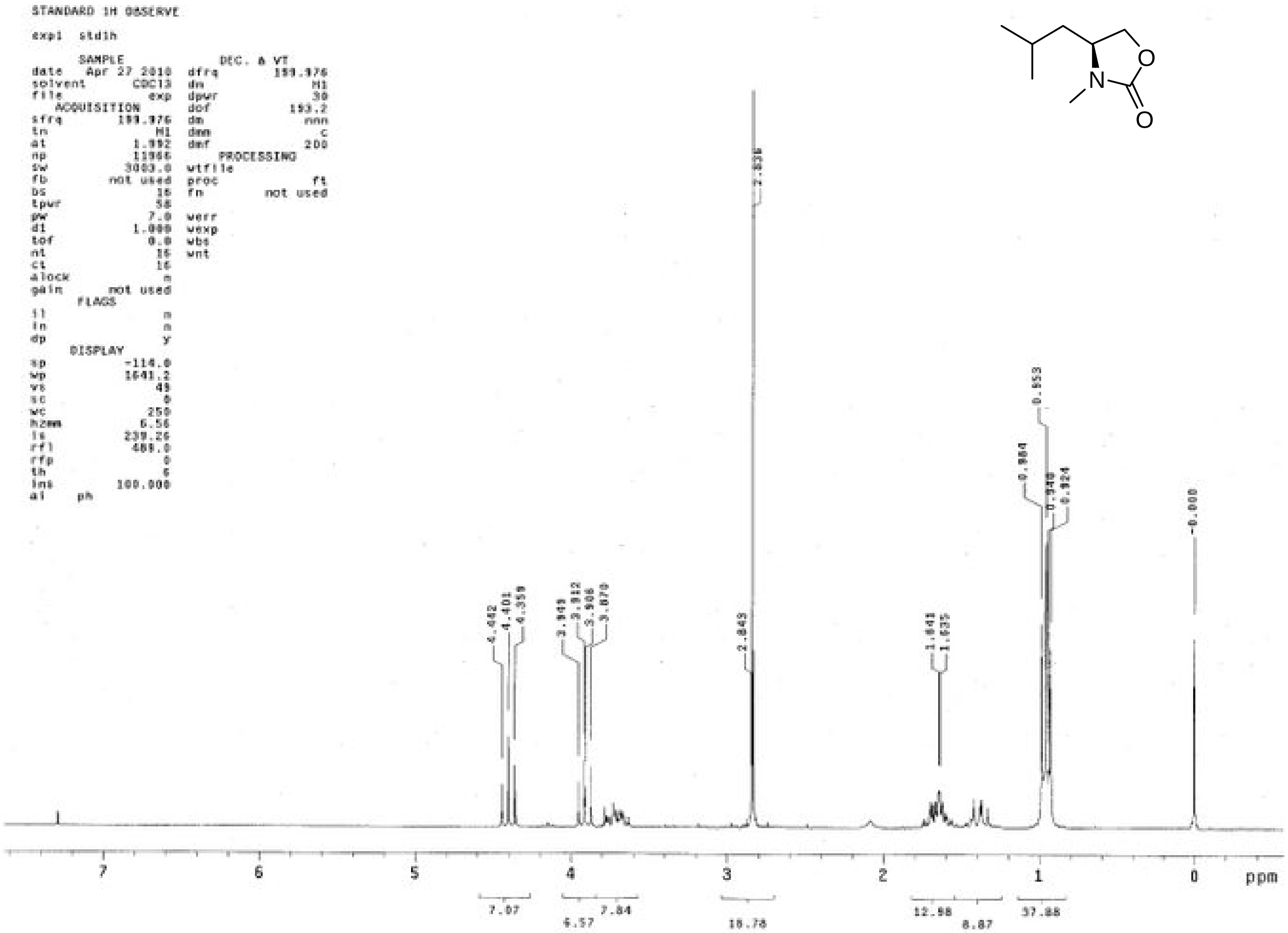

Figure 5: ^{1}H NMR spectrum of (*S*)-4-isobutyl-3-methyloxazolidin-2-one (**11**)

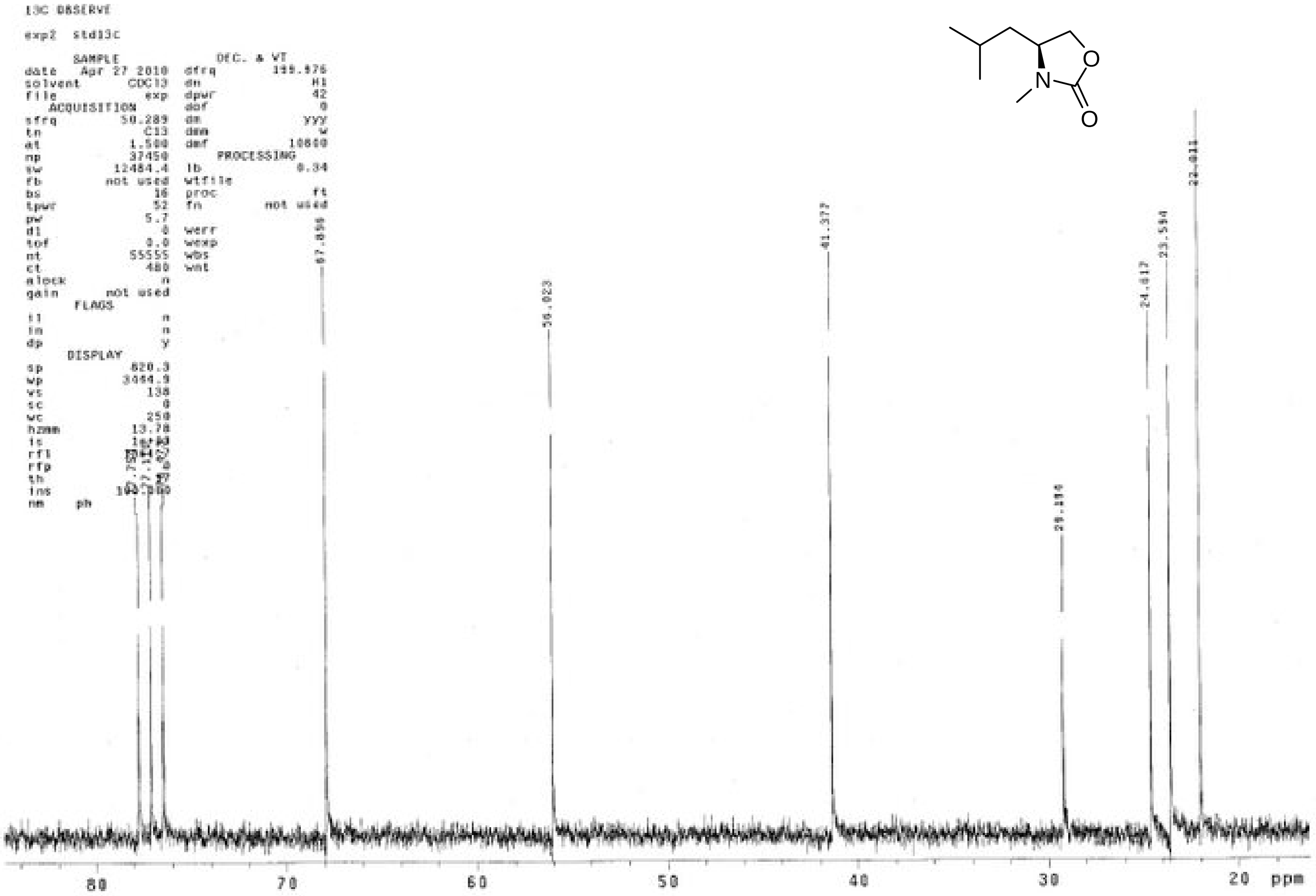

Figure 6: ^{13}C NMR spectrum of (*S*)-4-isobutyl-3-methyloxazolidin-2-one (**11**)

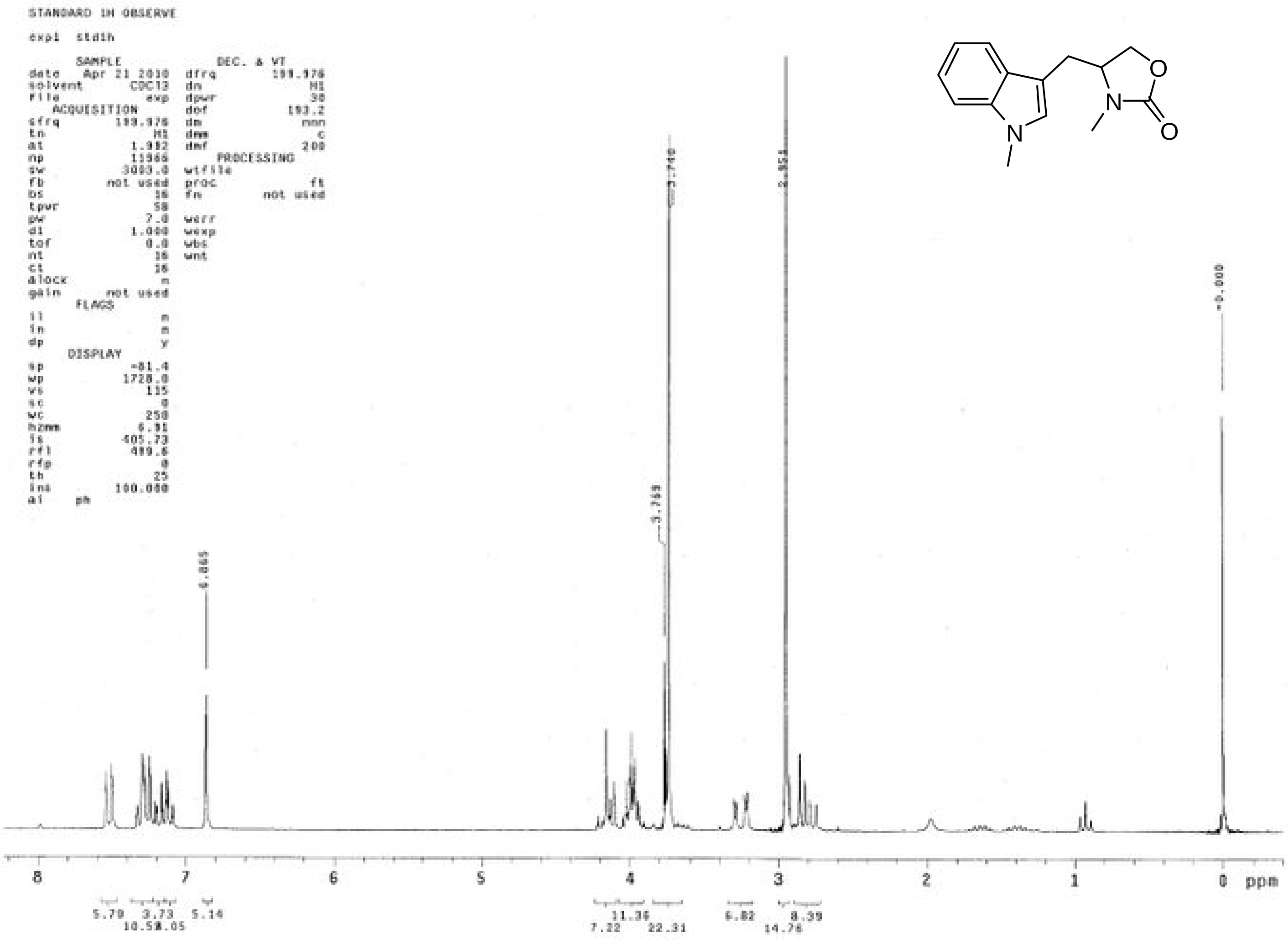

Figure 7: ^{1}H NMR spectrum of 3-methyl-4-((1-methyl-1*H*-indol-3-yl)methyl)oxazolidin-2-one (**12**)

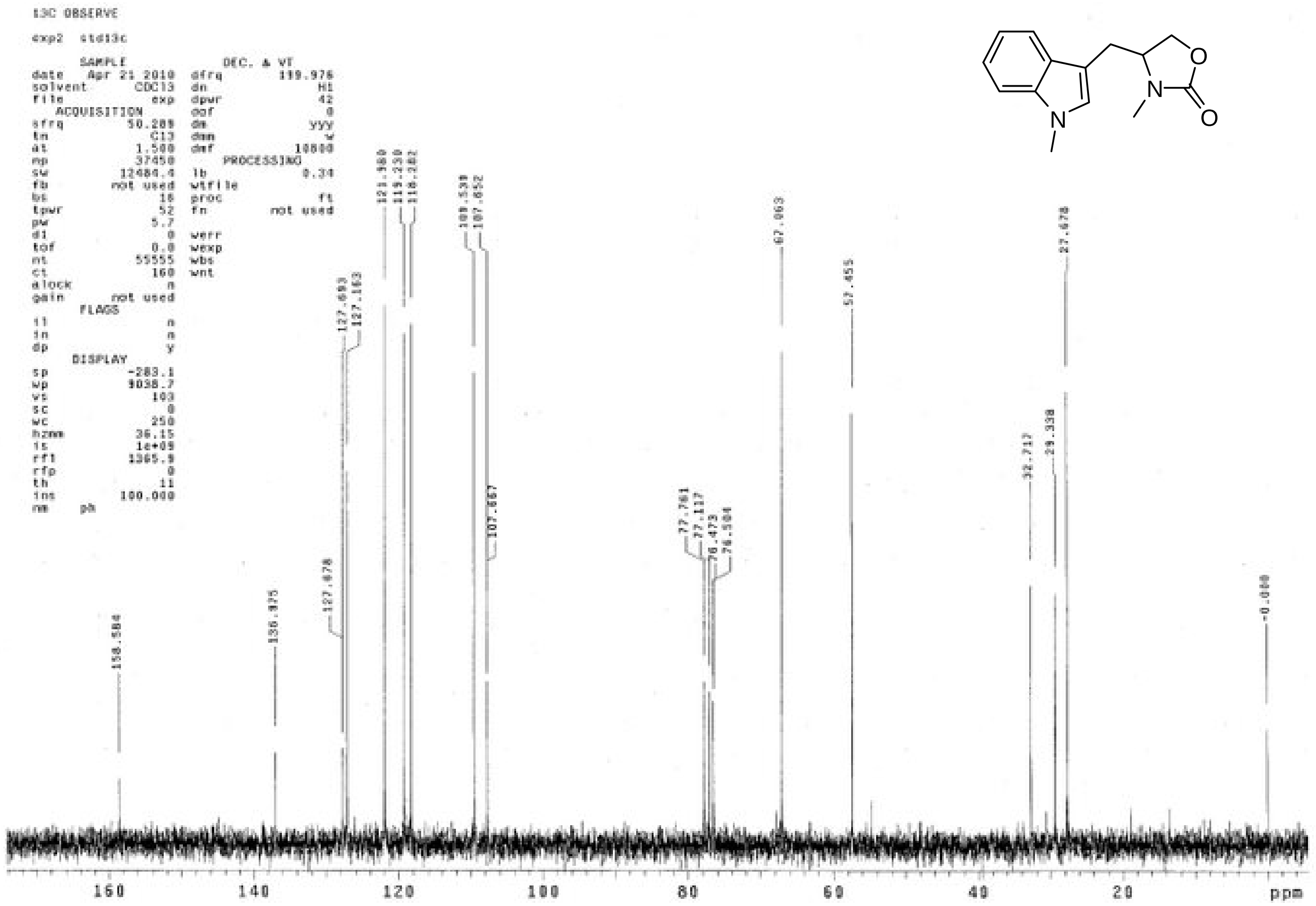

Figure 8:^{13}C NMR spectrum of 3-methyl-4-((1-methyl-1*H*-indol-3-yl)methyl)oxazolidin-2-one (**12**)

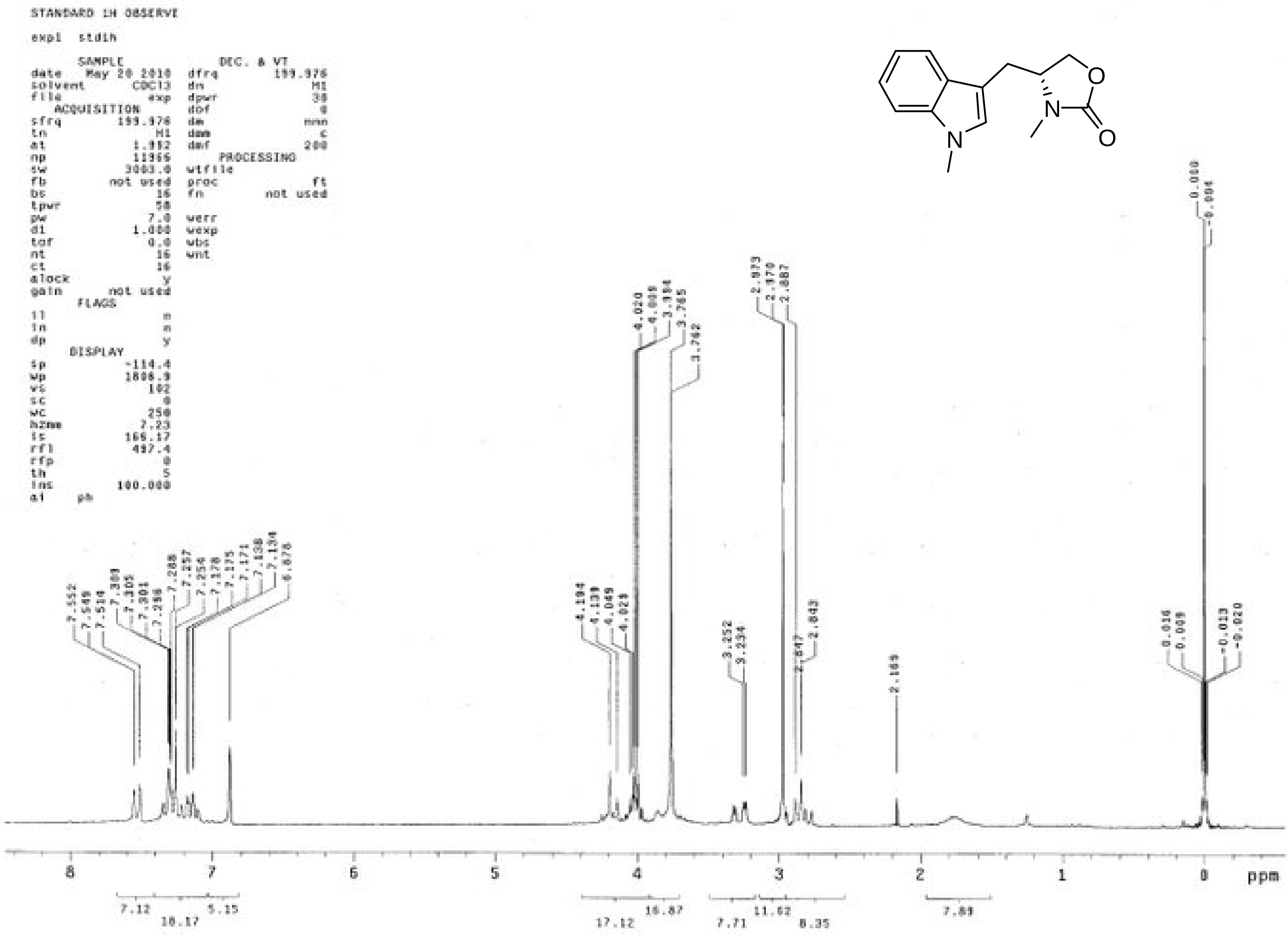

Figure 9: ^{1}H NMR spectrum of (*R*)-3-methyl-4-((1-methyl-1*H*-indol-3-yl)methyl)oxazolidin-2-one (**13**)

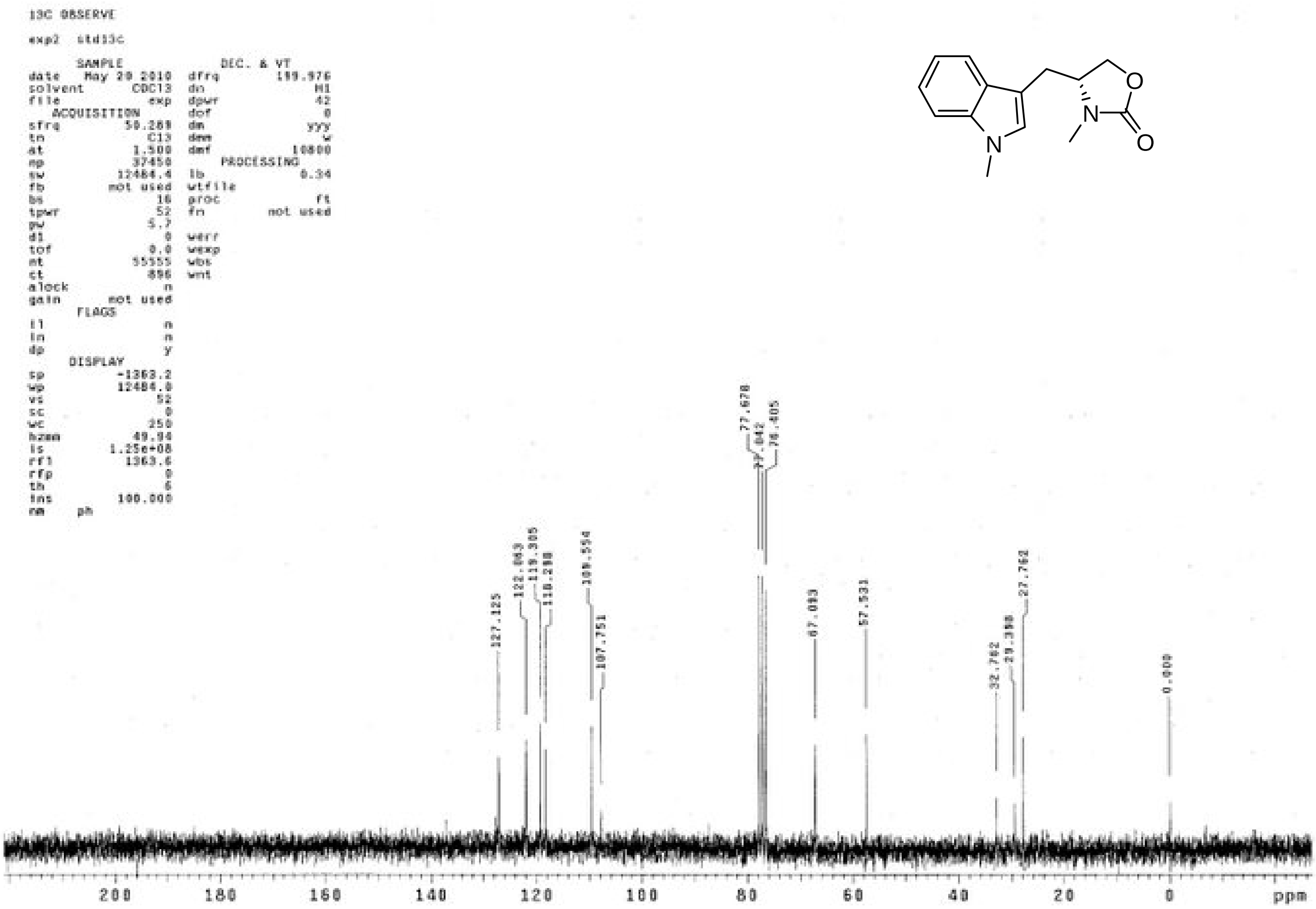

Figure 10:[1]C NMR spectrum of (*R*)-3-methyl-4-((1-methyl-1*H*-indol-3-yl)methyl)oxazolidin-2-one (**13**)

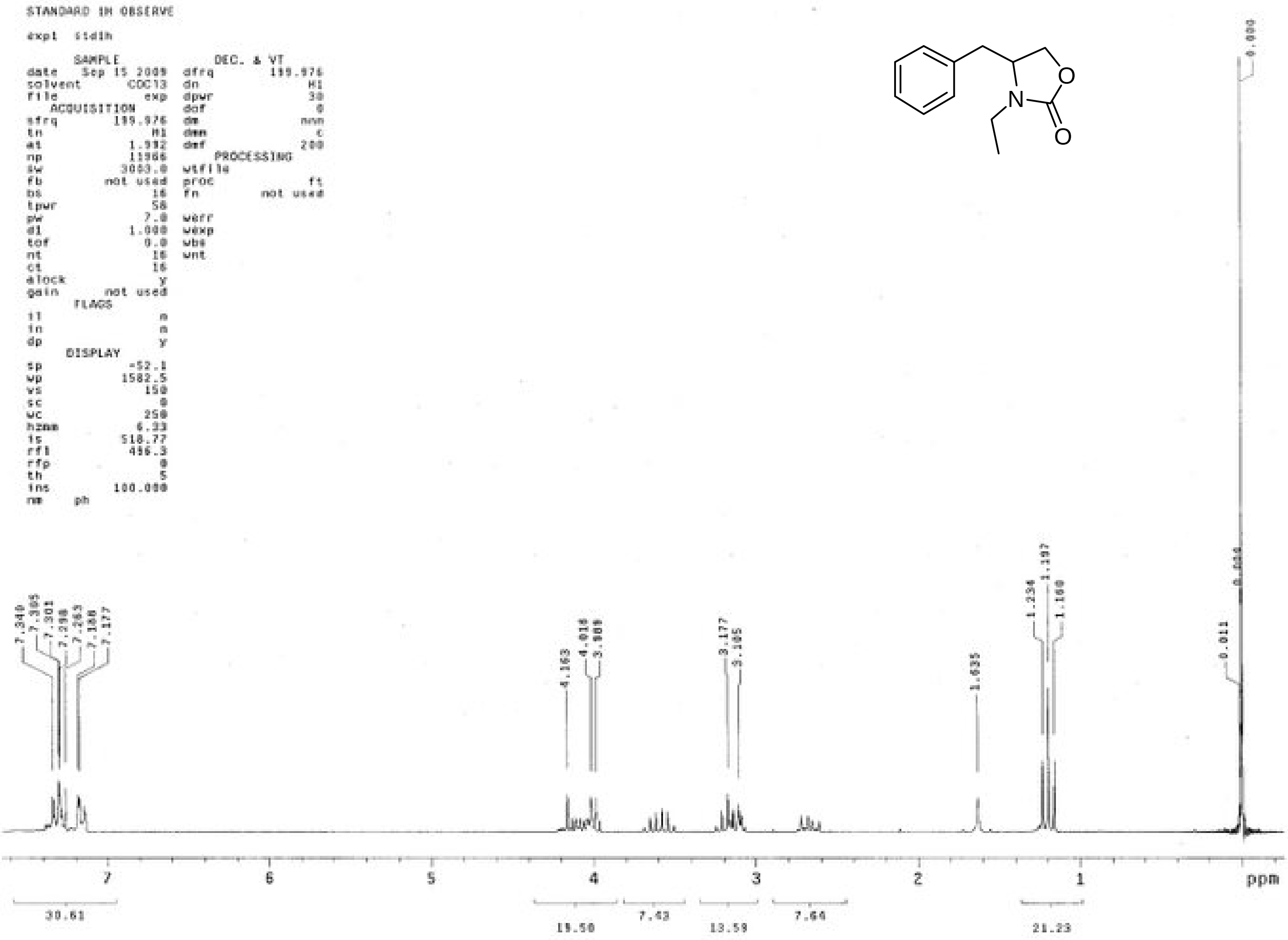

Figure 11: ^{1}H NMR spectrum of 4-benzyl-3-ethyloxazolidin-2-one (**17**)

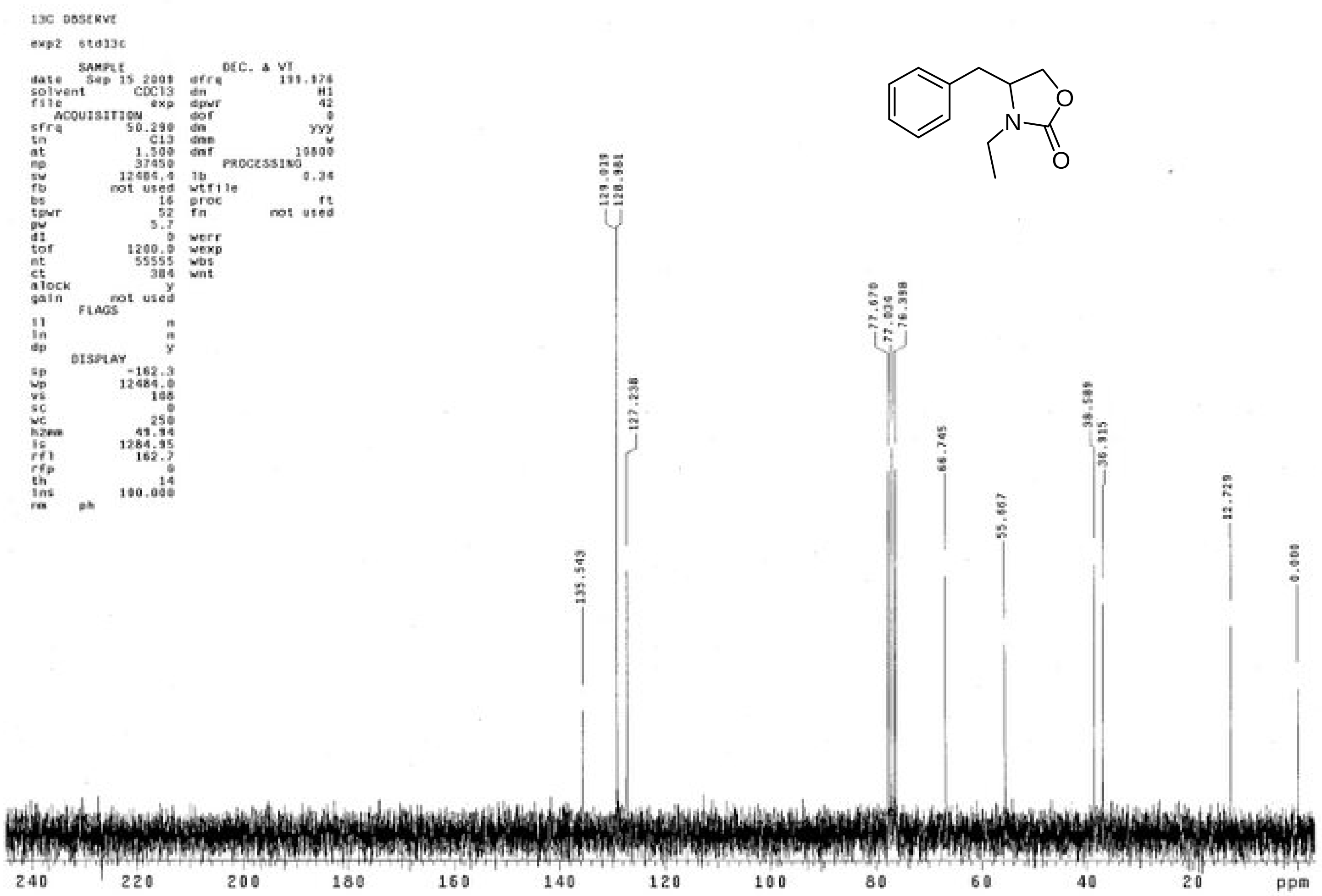

Figure 12: ^{13}C NMR spectrum of 4-benzyl-3-ethyloxazolidin-2-one (**17**)

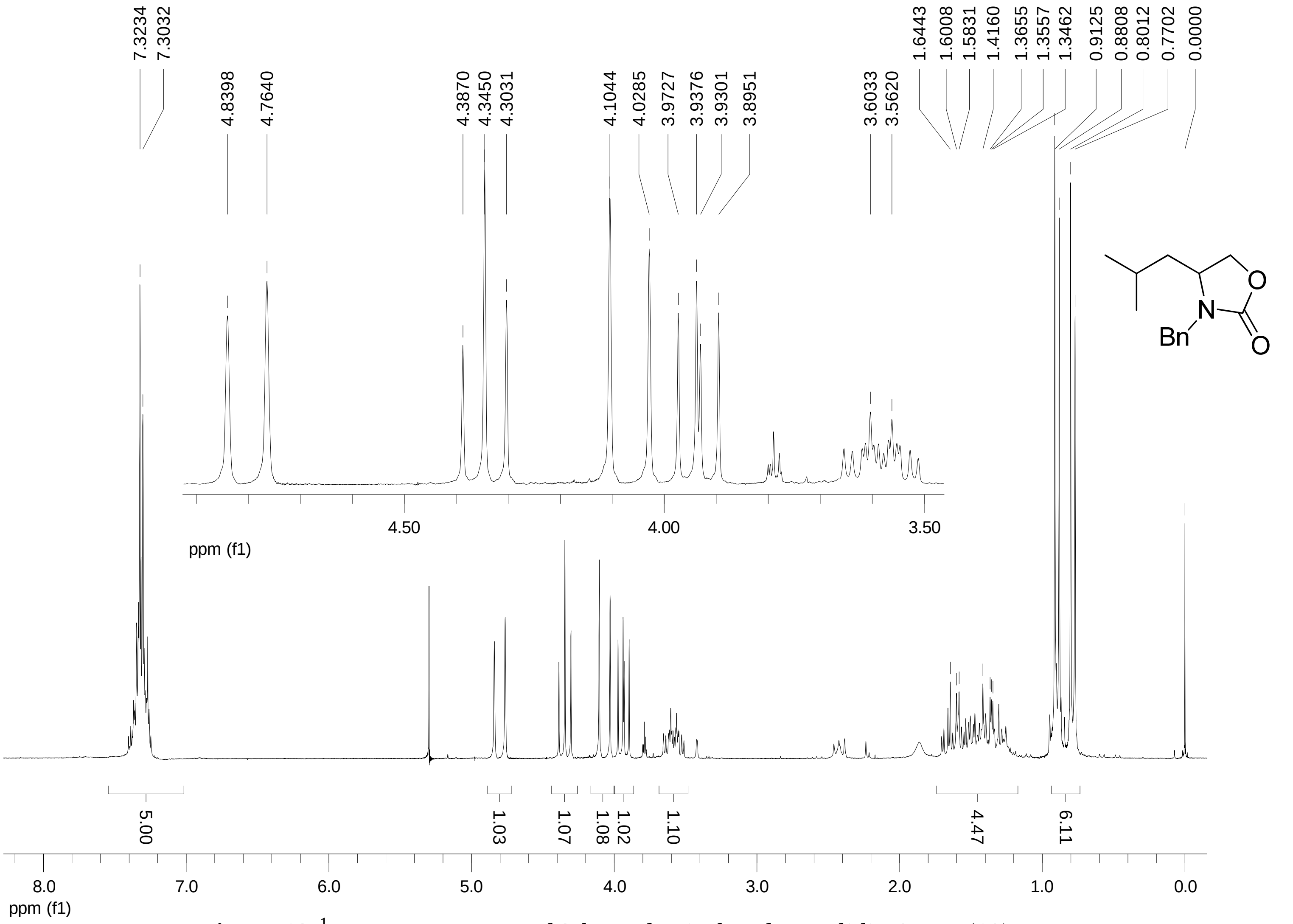

Figure 13: ^{1}H NMR spectrum of 3-benzyl-4-isobutyloxazolidin-2-one (**19**)

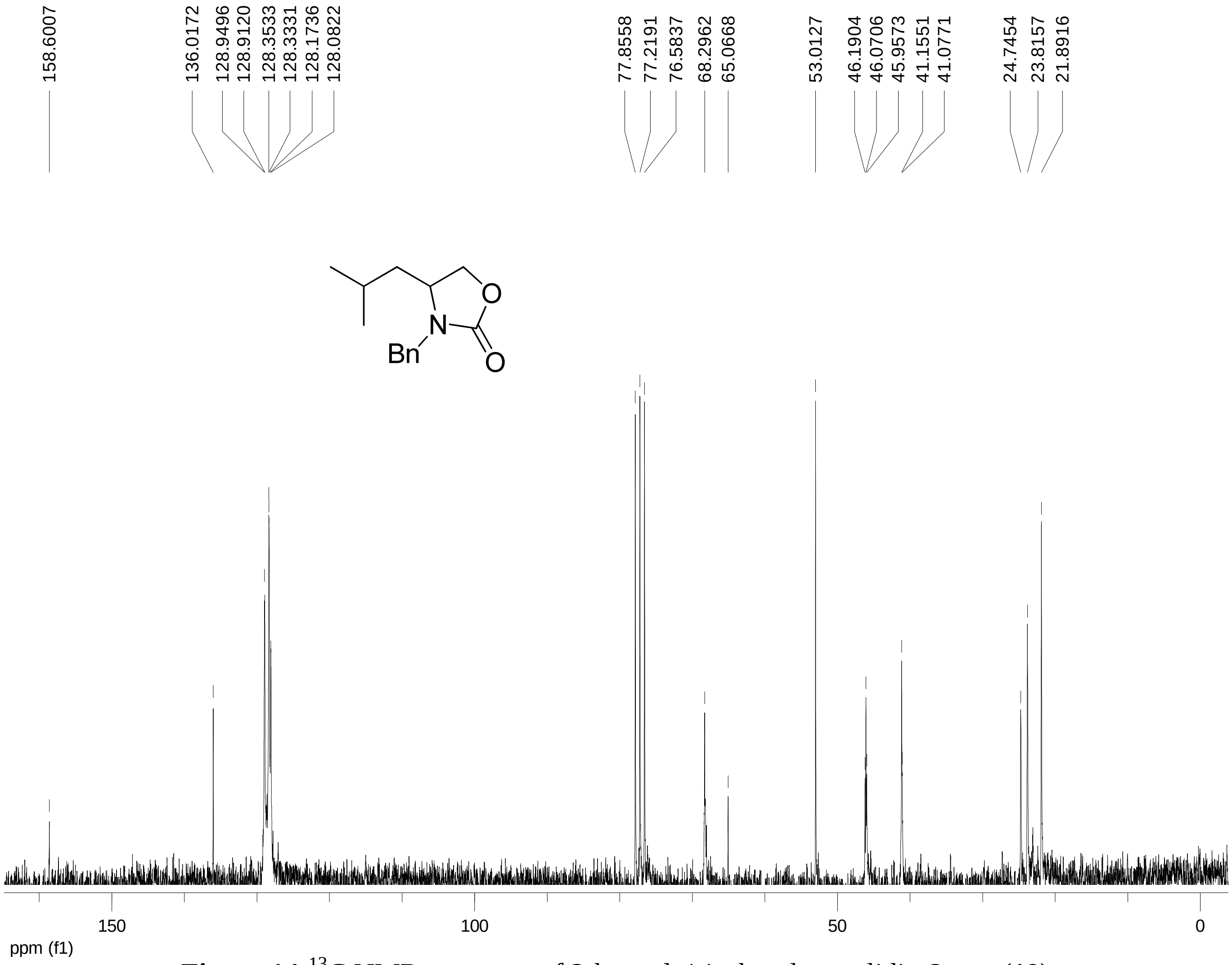

Figure 14:^{13}C NMR spectrum of 3-benzyl-4-isobutyloxazolidin-2-one (**19**)

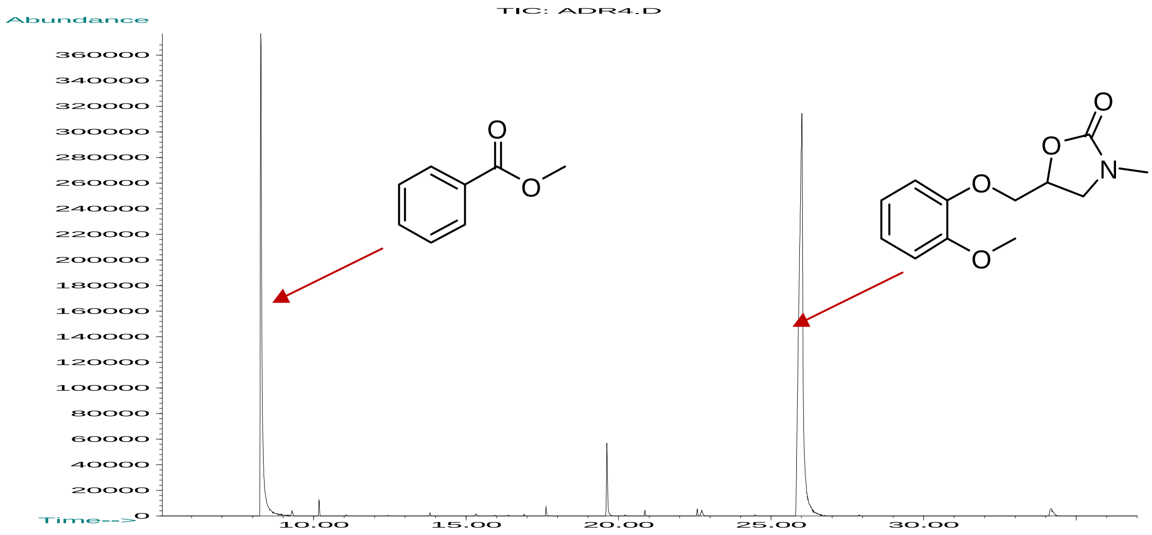

Figure 15: GC of reaction products 5-((2-methoxyphenoxy)-3-methyloxazolidin-2-one (**16**) and methyl benzoate.

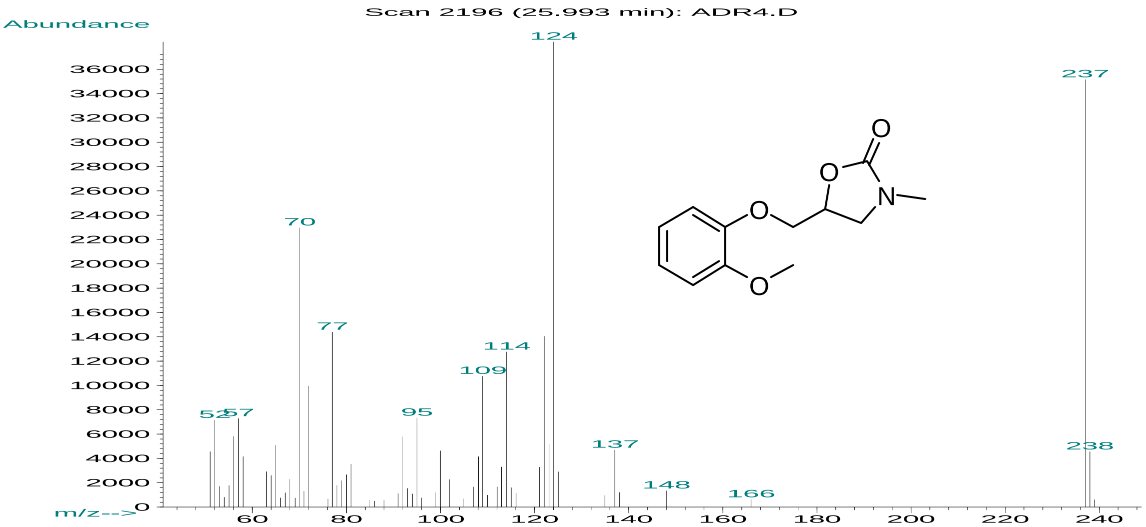

Figure 16: MS of 5-((2-methoxyphenoxy)-3-methyloxazolidin-2-one (**16**)

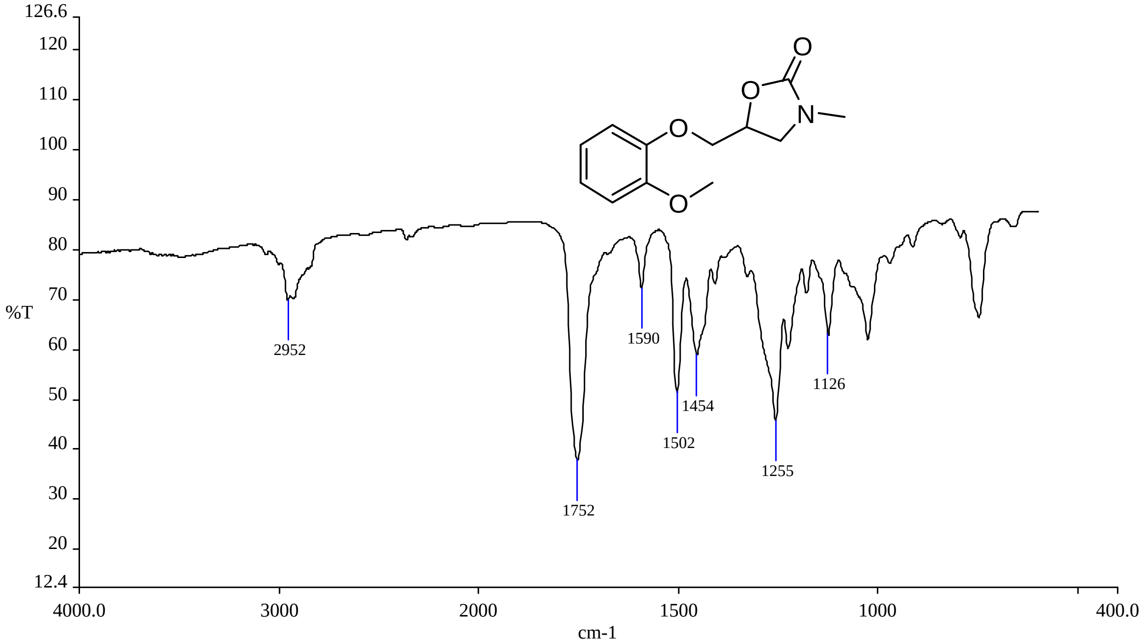

Figure 17: IR spectrum of 5-((2-methoxyphenoxy)-3-methyloxazolidin-2-one (**16**)

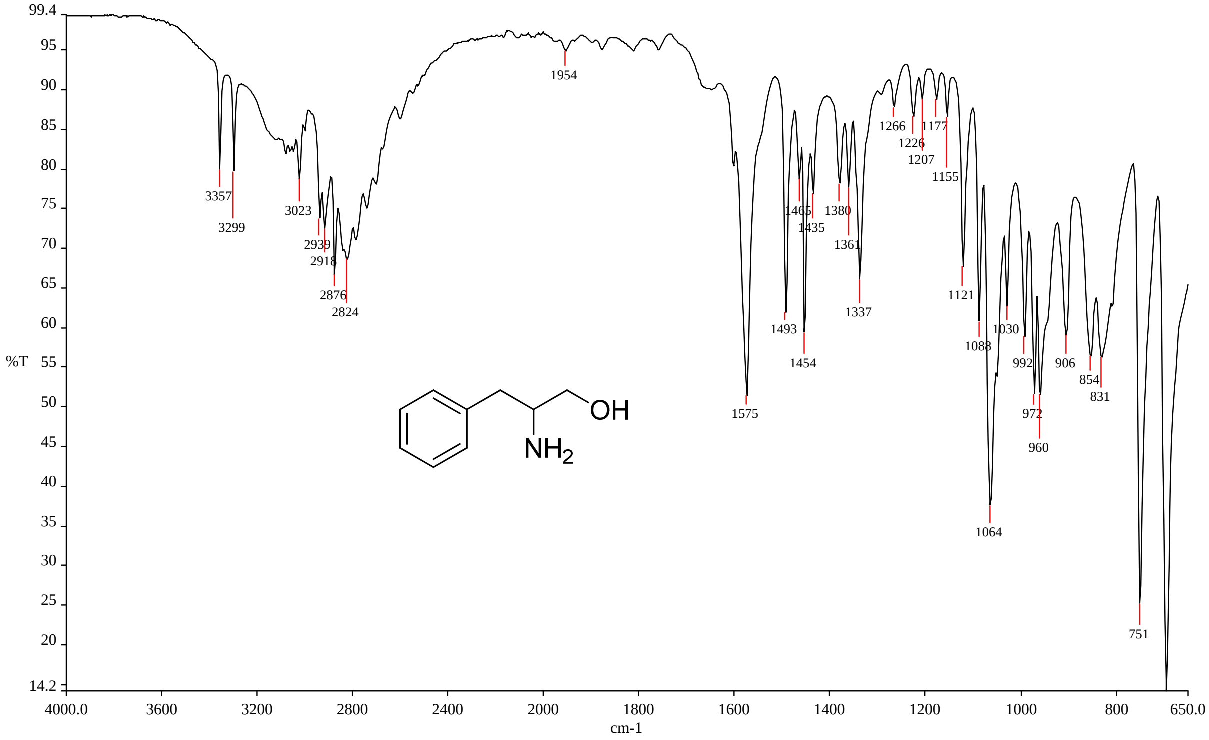

Figure 18: IR spectrum of 2-amino-3-phenylpropan-1-ol (**1**)

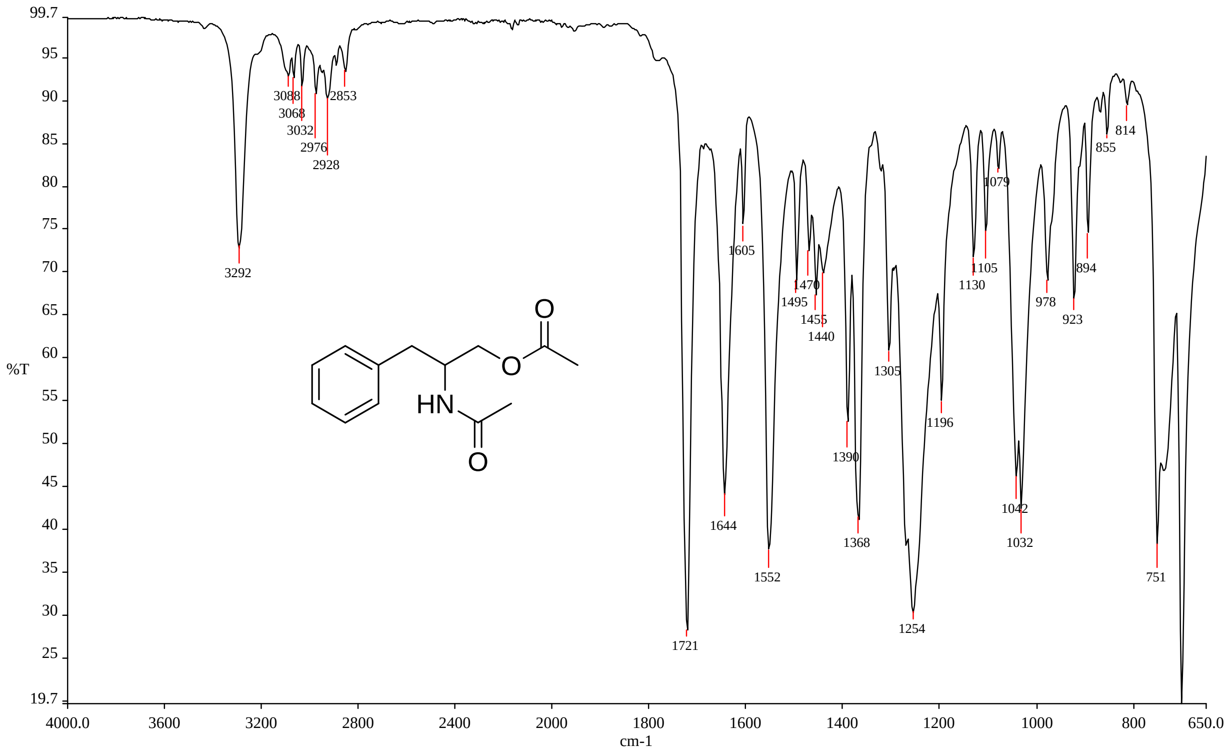

Figure 19: IR spectrum of 2-acetamido-3-phenylpropyl acetate (**3**)

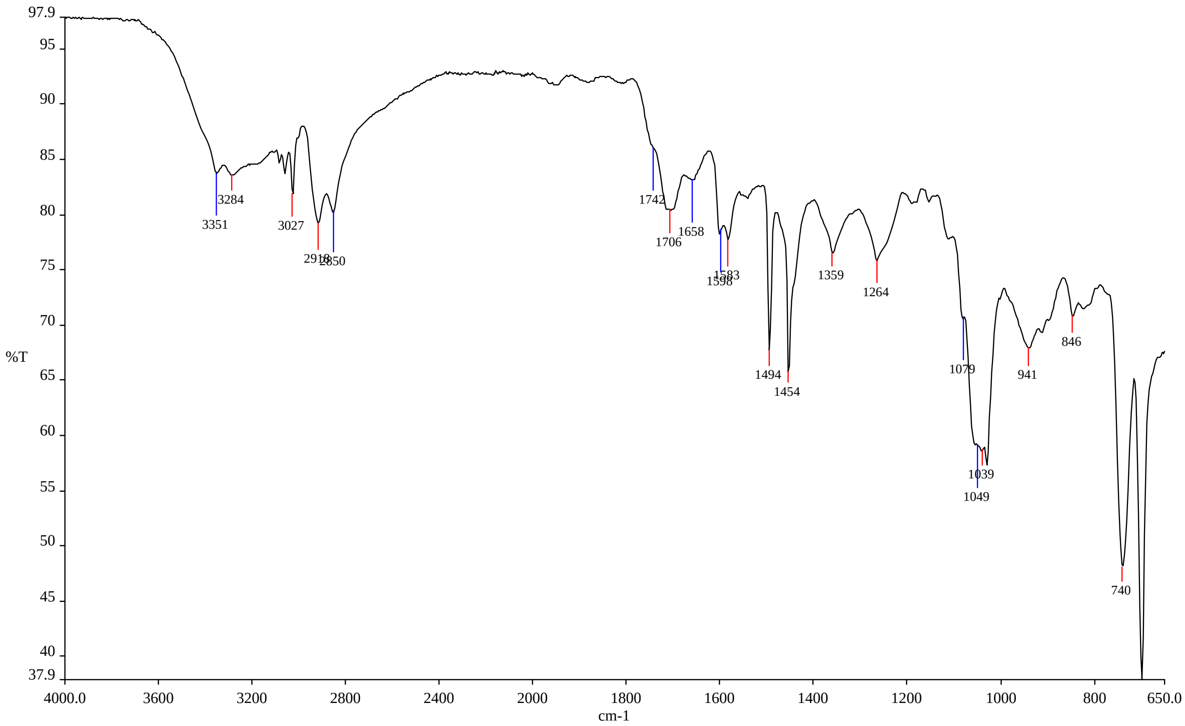

Figure 20:IR spectrum of crude obtained in conditions described in Table 2, entry 1

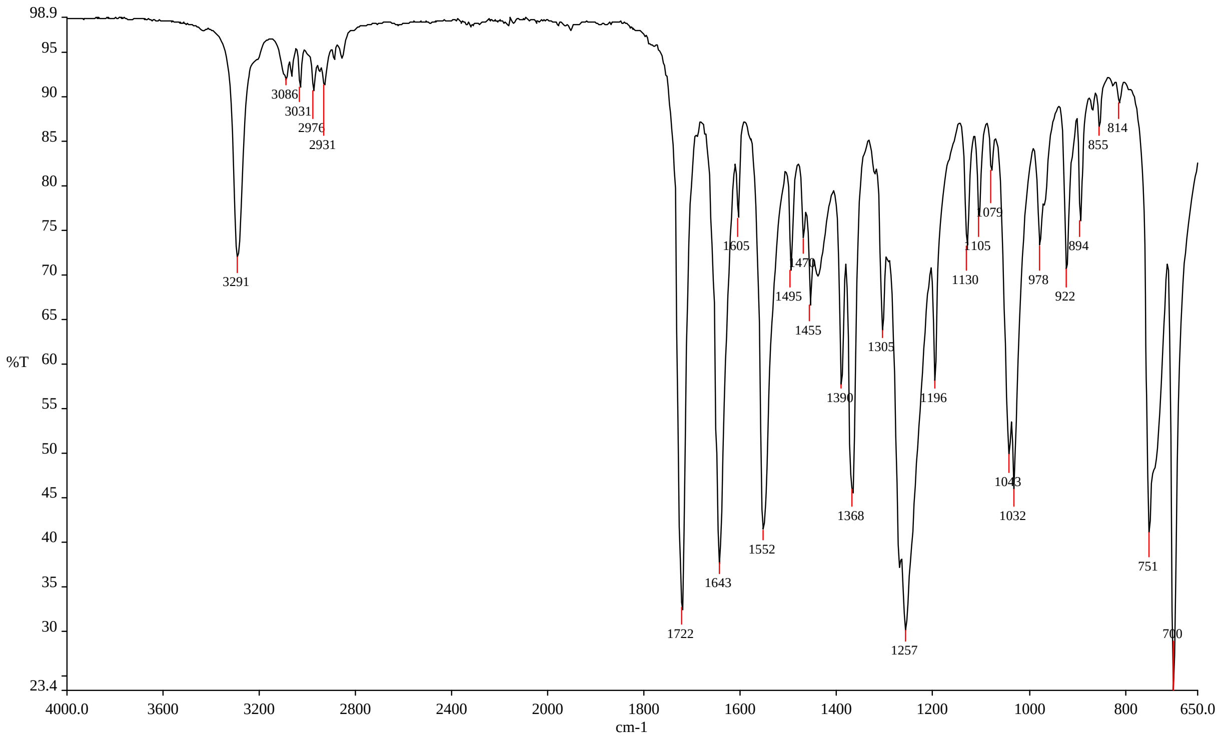

Figure 21: IR spectrum of crude product described in Table 2, entry 2.

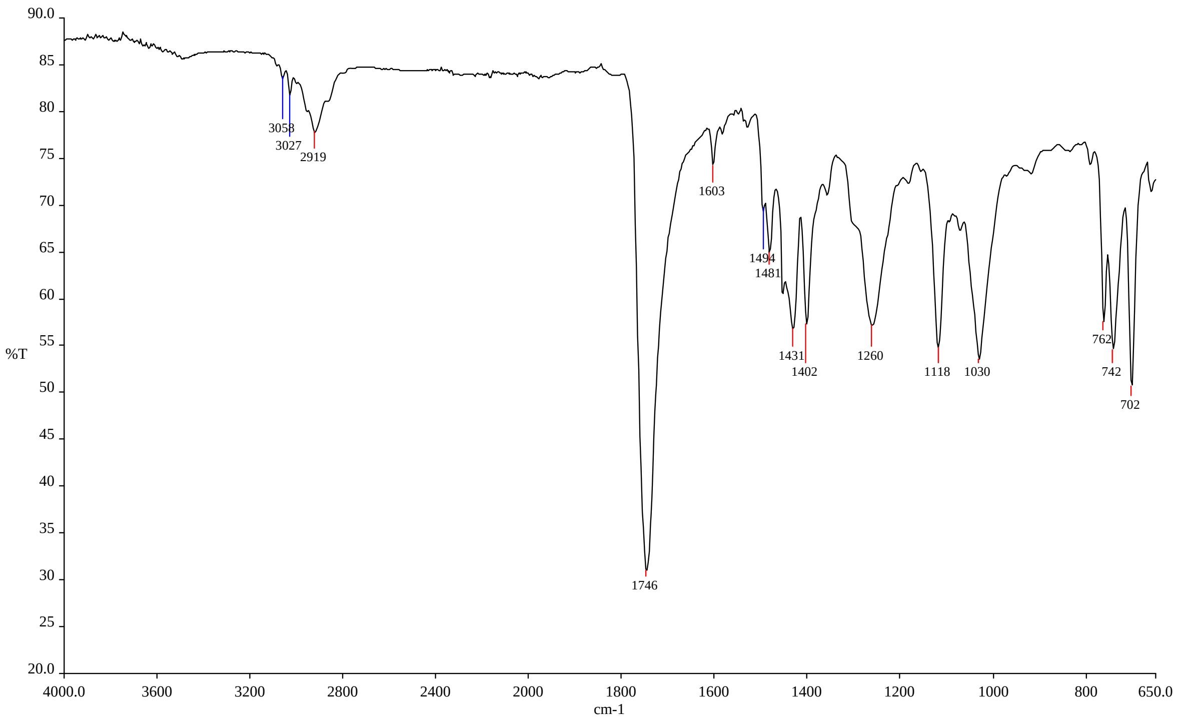

Figure 22: IR spectrum of crude obtained in conditions described in Table 2, entry 3

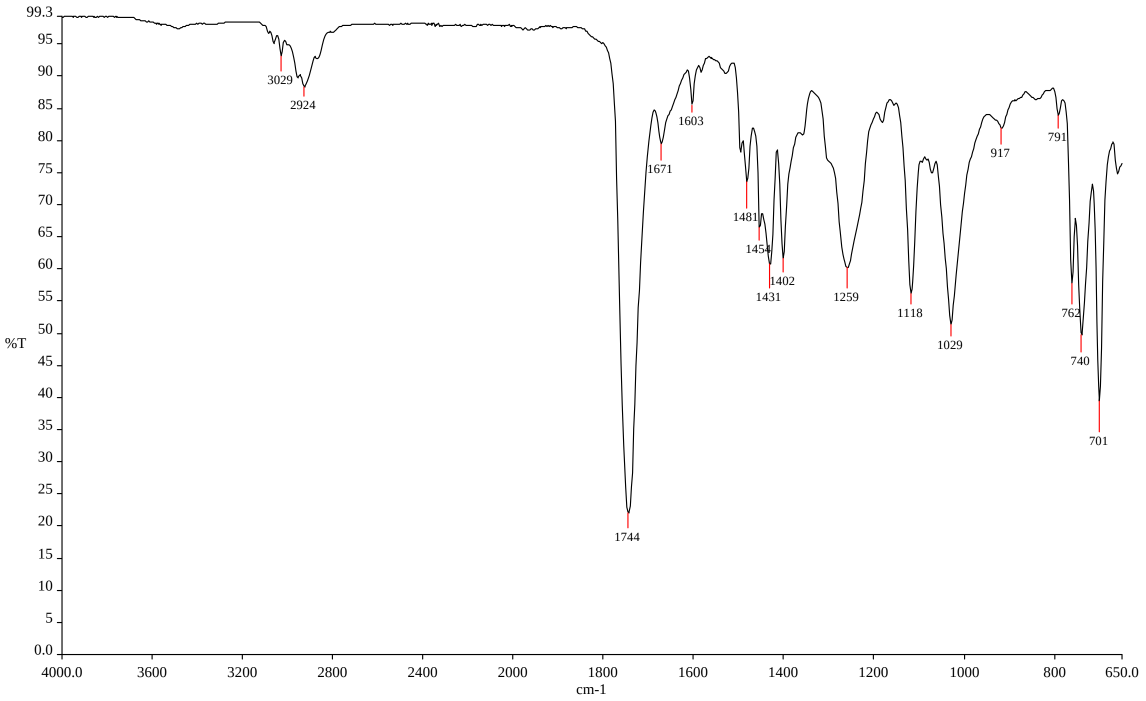

Figure 23: IR spectrum of crude obtained in conditions described in Table 2, entry 4

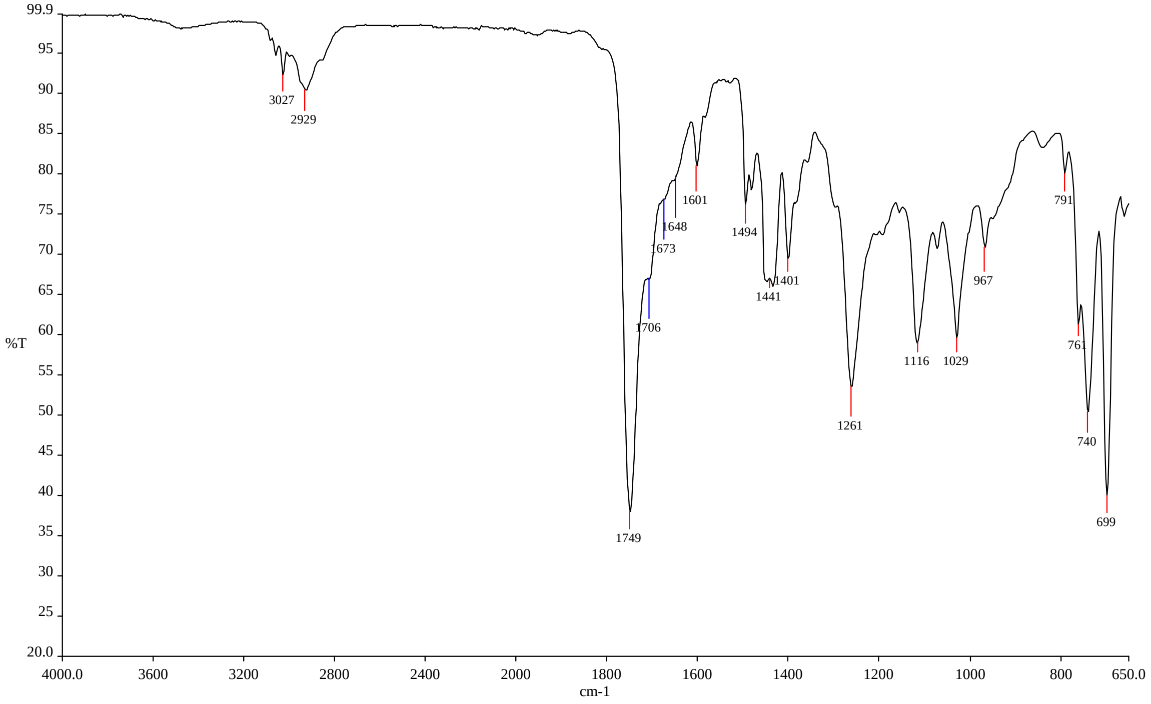

Figure 24.IR spectrum of crude obtained in conditions described in Table 2, entry 5

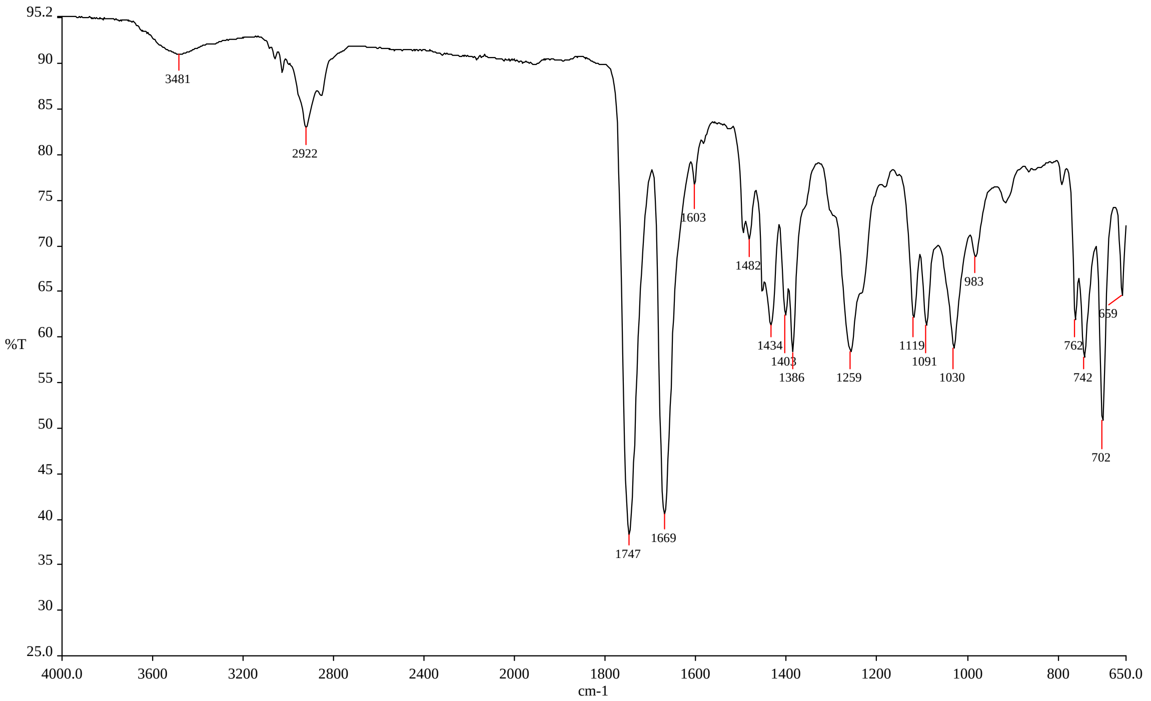

Figure 25: IR spectrum of crude obtained in conditions described in Table 2, entry 6

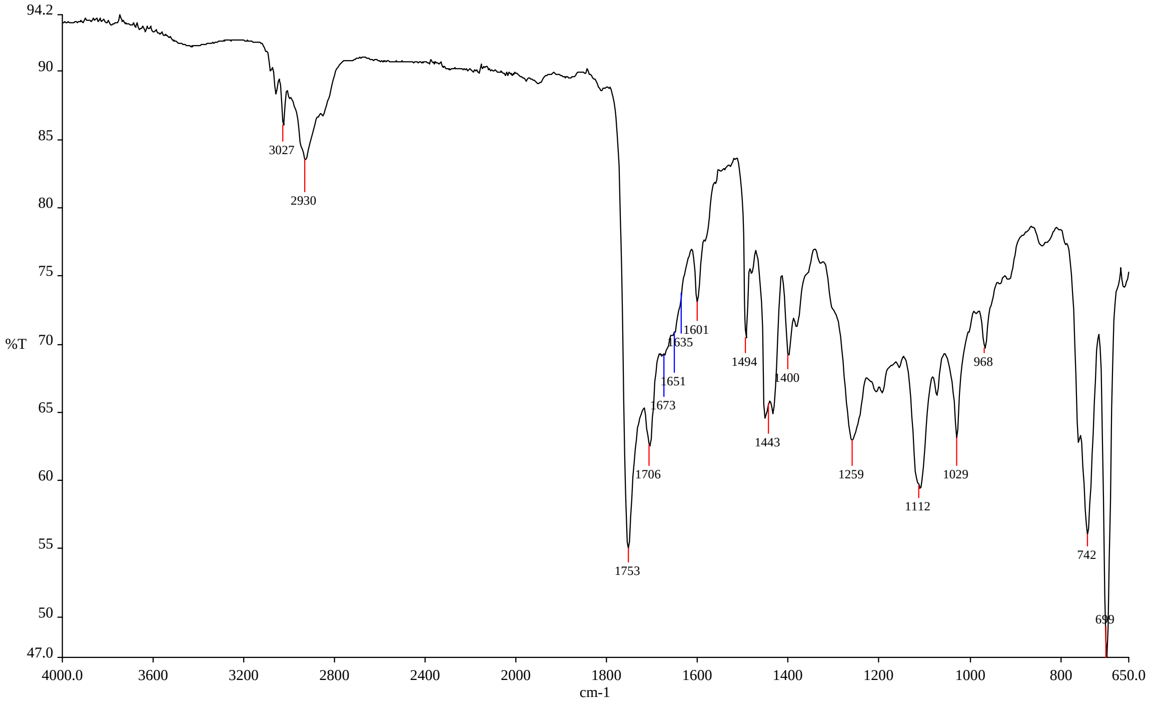

Figure 26: IR spectrum of crude obtained in conditions described in Table 2, entry 7

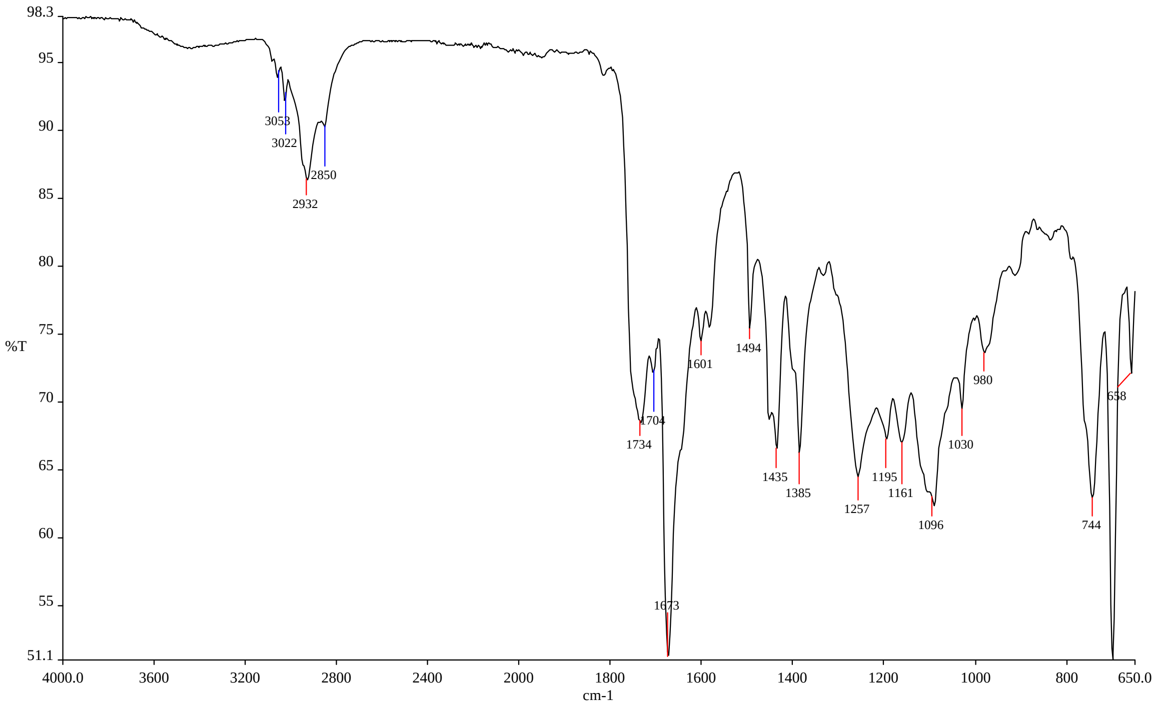

Figure 27: IR spectrum of crude obtained in conditions described in Table 2, entry 8

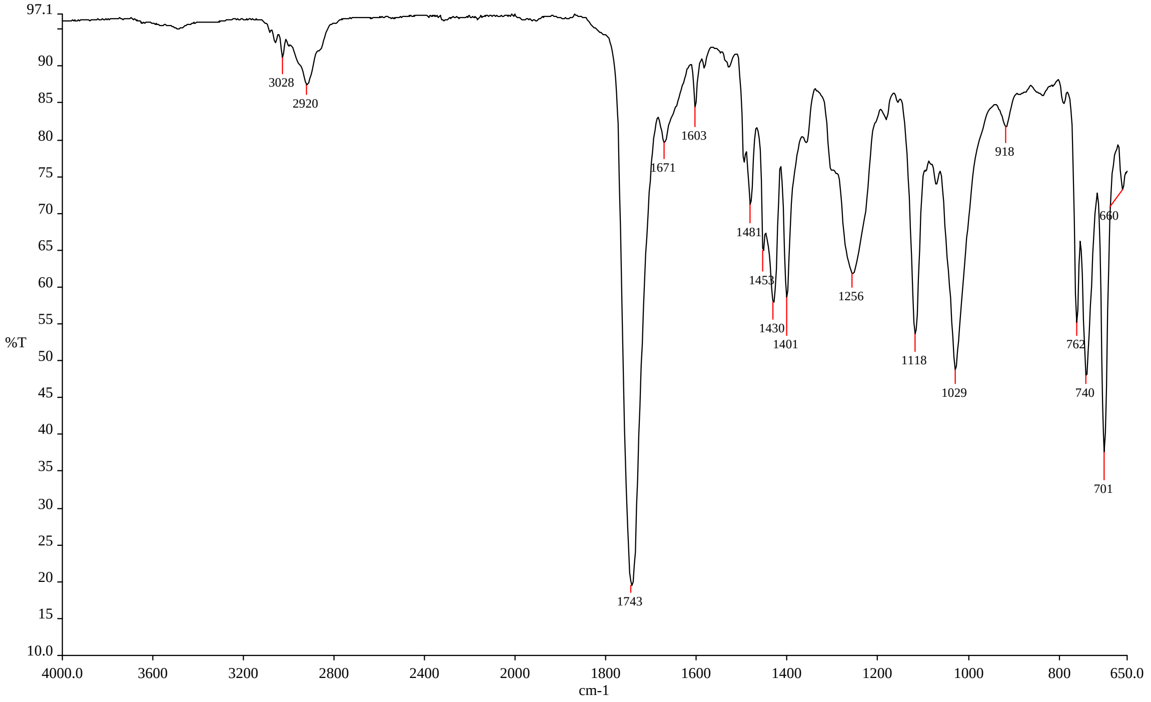

Figure 28: IR spectrum of crude obtained in conditions described Table 2, entry 9

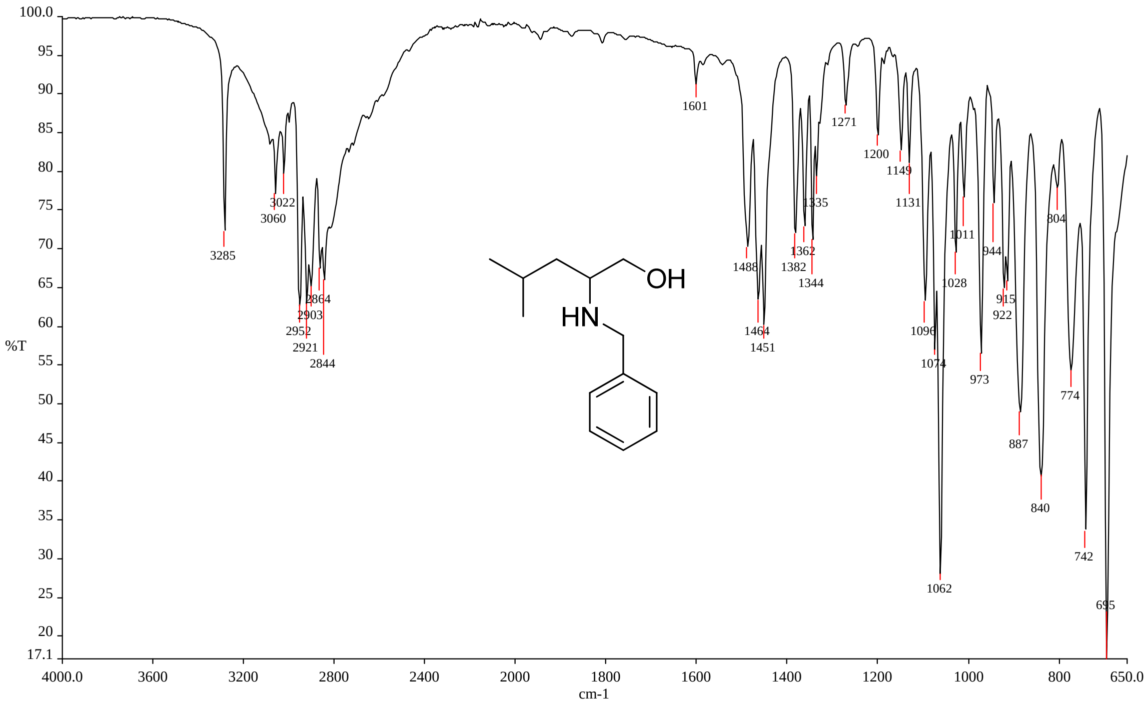

Figure 29: IR spectrum of 2-(benzylamino)-4-methylpentan-1-ol (**18**)

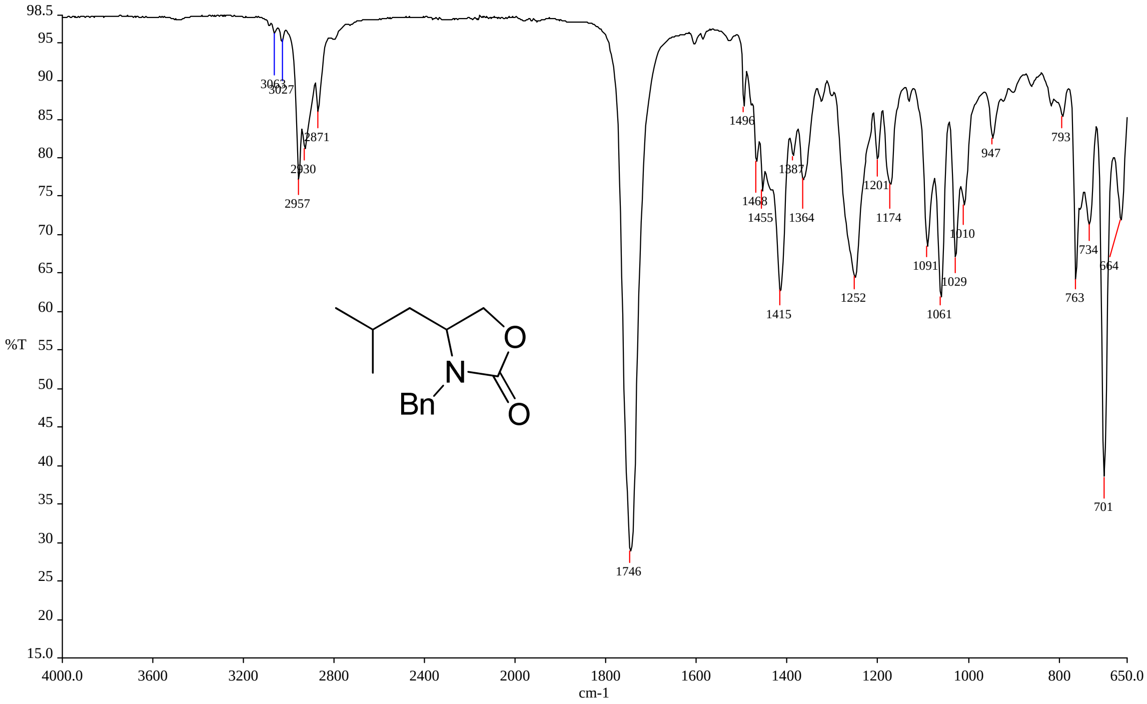

Figure 30: IR spectrum of crude obtained in conditions described in Table 2, entry 10

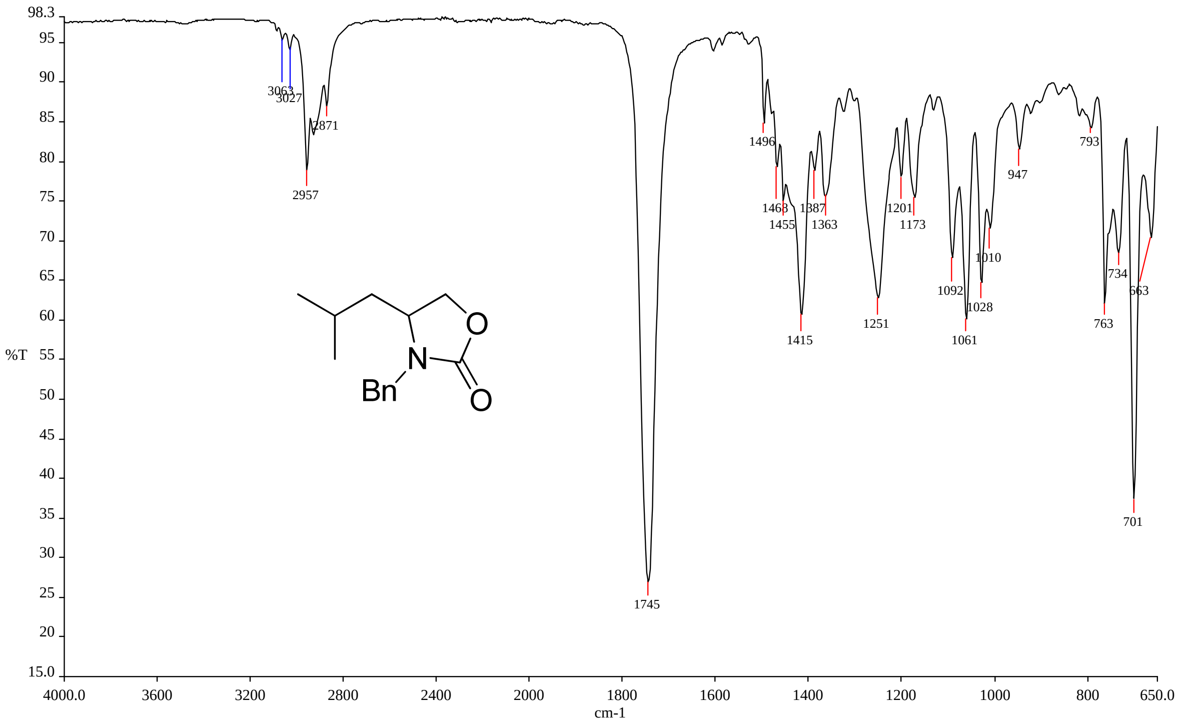

Figure 31: IR spectrum of crude obtained in conditions described in Table 2, entry 11

File   :C:\MSDCHEM\1\DATA\OCHOA\Snapshot\ADRIAN72.d
Operator   : D. CHAVEZ
Acquired   : 12 Aug 2014 13:43   using AcqMethod VOLATILESPESADOS.M
Instrument :   Numero 1
Sample Name: TETRABUTILAMINA
Misc Info :
Vial Number: 5

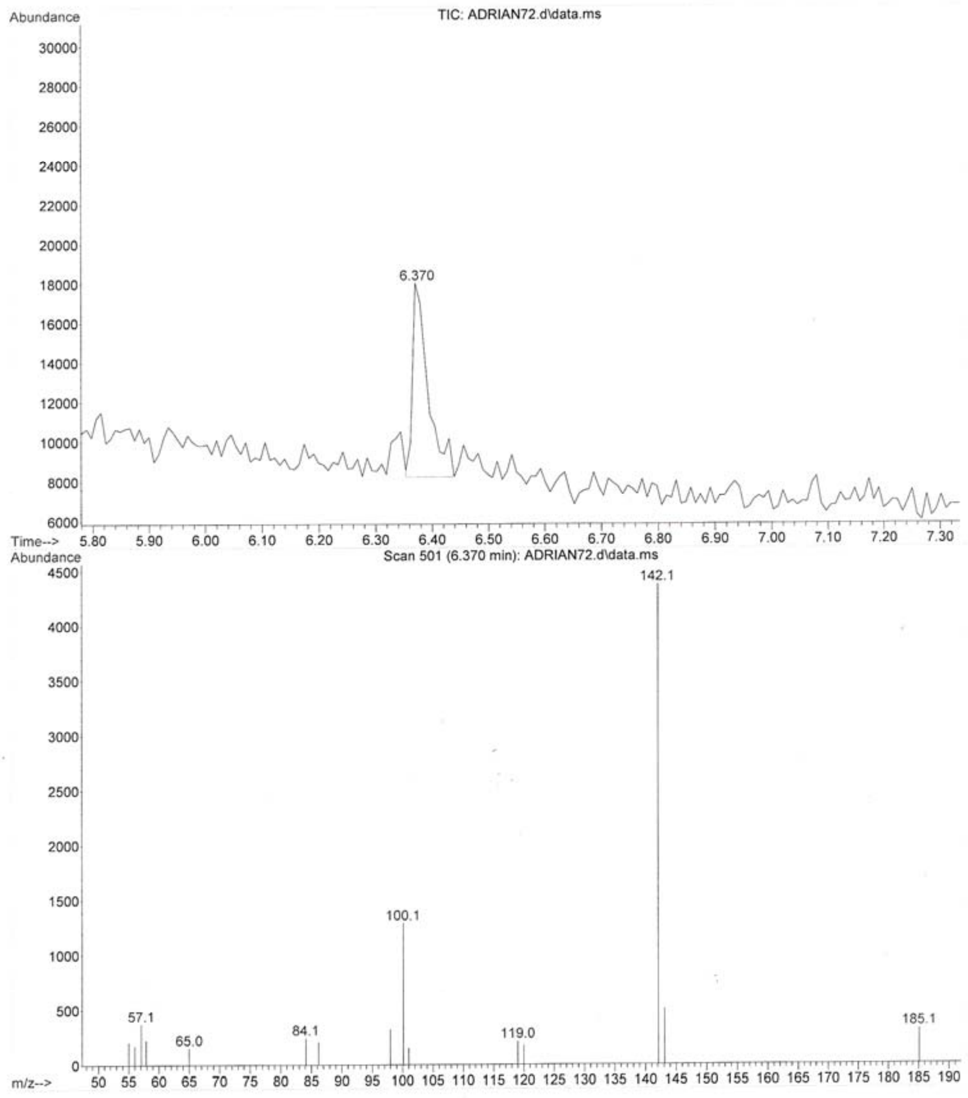

Figure 32:GC-MS of TBAC in solution (part 1).

Hit 1 : Tributylamine
C12H27N; MF: 738; RMF: 774; Prob 12.8%; CAS: 102-82-9; Lib: mainlib; ID: 104480.

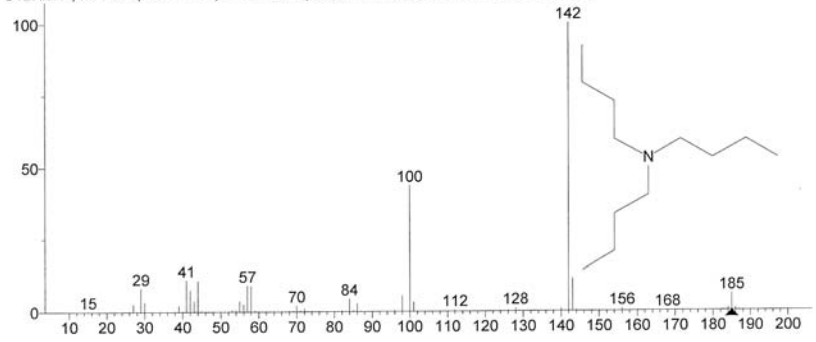

(mainlib) Tributylamine

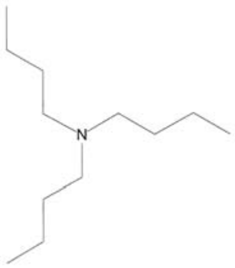

Name: Tributylamine
Formula: $C_{12}H_{27}N$
MW: 185 CAS#: 102-82-9 NIST#: 291265 ID#: 104480 DB: mainlib
Other DBs: Fine, TSCA, RTECS, HODOC, EINECS, IRDB
Contributor: NIST Mass Spectrometry Data Center, 1998.
10 largest peaks:
142 999 | 100 435 | 143 112 | 41 110 | 44 107 | 57 91 | 58 90 | 29 82 | 42 75 | 185 58 |
Synonyms:
1.1-Butanamine, N,N-dibutyl-
2.Tri-n-butylamine
3.Tris[N-butylamine]
4.(n-C4H9)3N
5.Tributilamina
6.UN 2542
7.N,N-Dibutyl-1-butanamine #

Page 1 of 1

Figure 33:GC-MS of TBAC in solution (part 2).

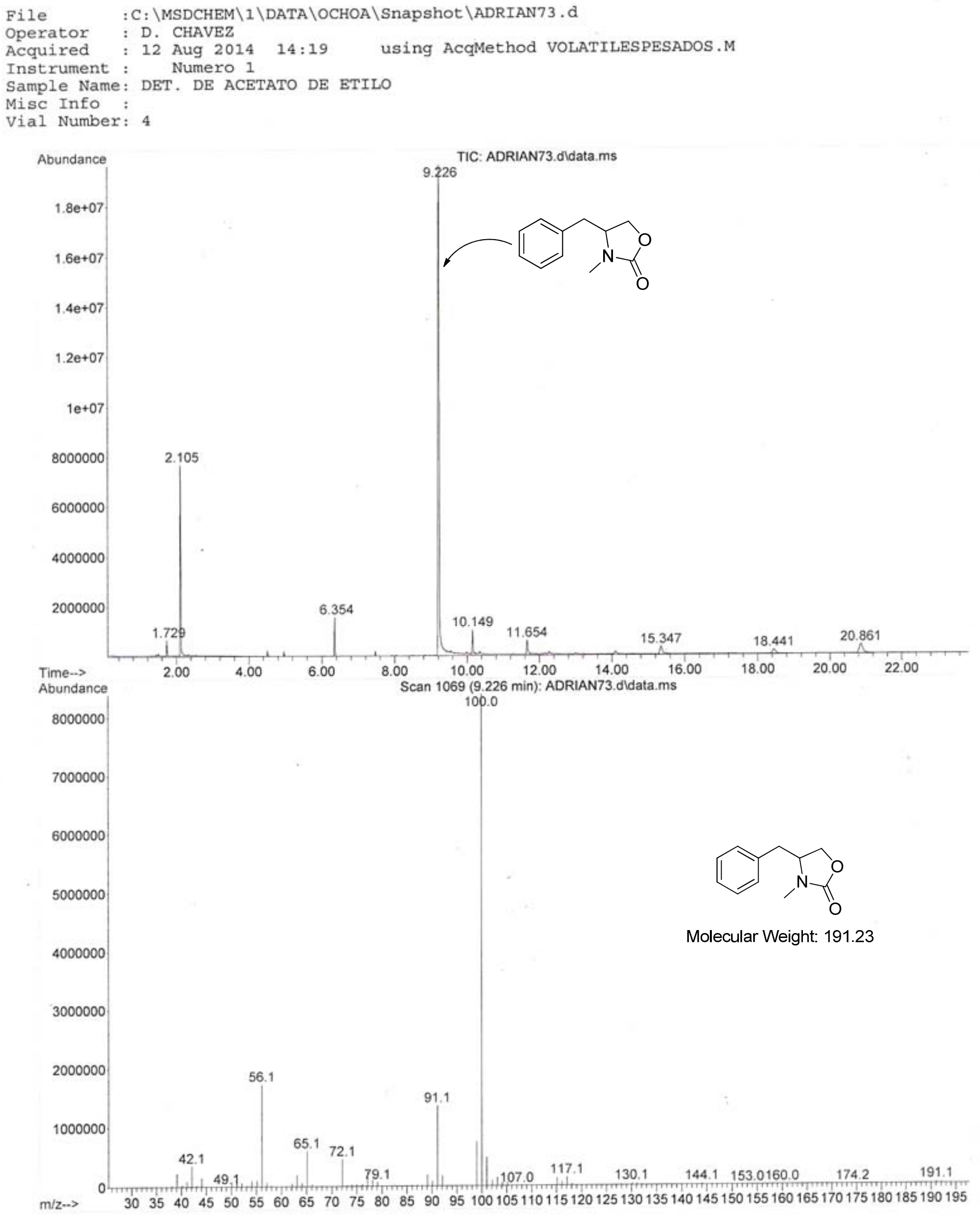

Figure 34:GC-MS of the crude of reaction obtained in conditions described in Table 2, entry 4 (part 1)

File   :C:\MSDCHEM\1\DATA\OCHOA\Snapshot\ADRIAN73.d
Operator   : D. CHAVEZ
Acquired   : 12 Aug 2014 14:19   using AcqMethod VOLATILESPESADOS.M
Instrument :   Numero 1
Sample Name: DET. DE ACETATO DE ETILO
Misc Info   :
Vial Number: 4

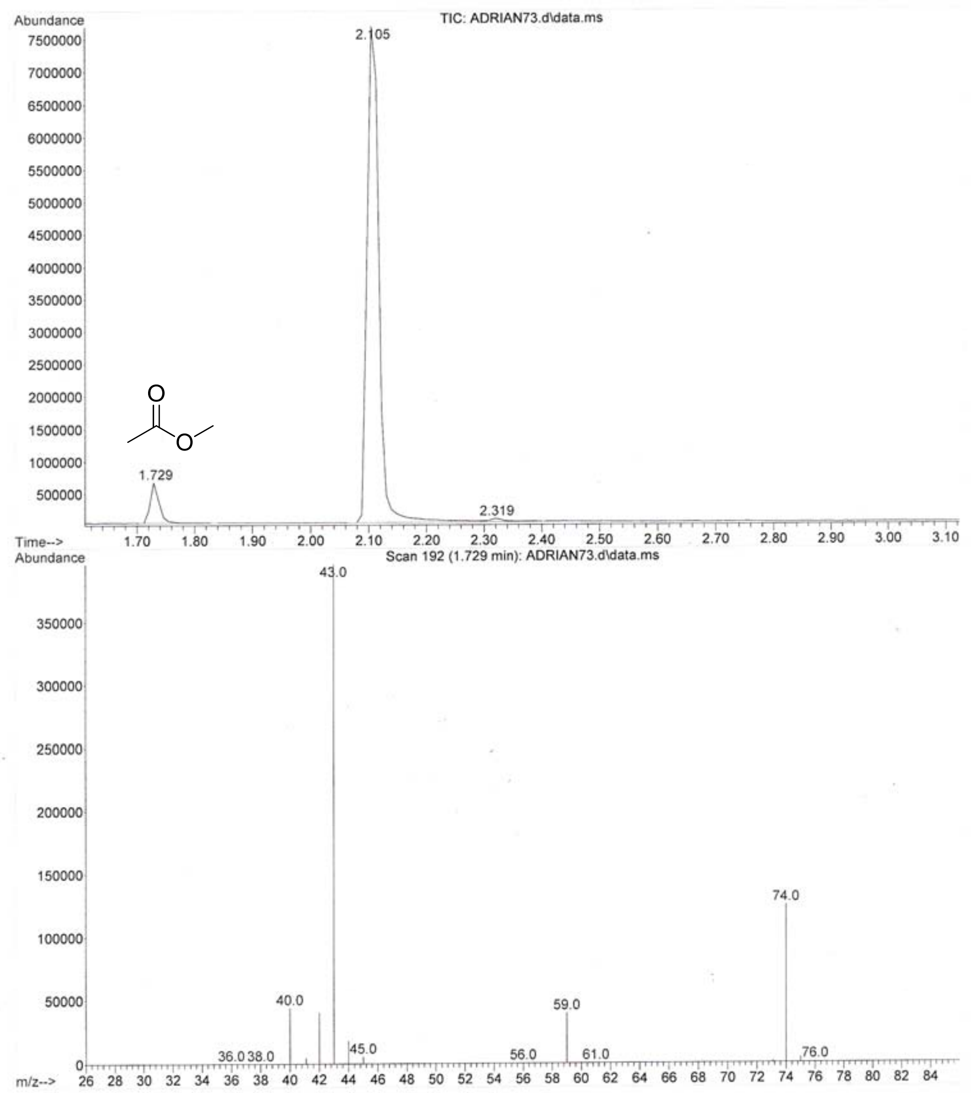

Figure 35:GC-MS of the crude of reaction obtained in conditions described in Table 2, entry 4 (part 2)

Hit 1 : Acetic acid, methyl ester
C3H6O2; MF: 903; RMF: 929; Prob 86.0%; CAS: 79-20-9; Lib: replib; ID: 2573.

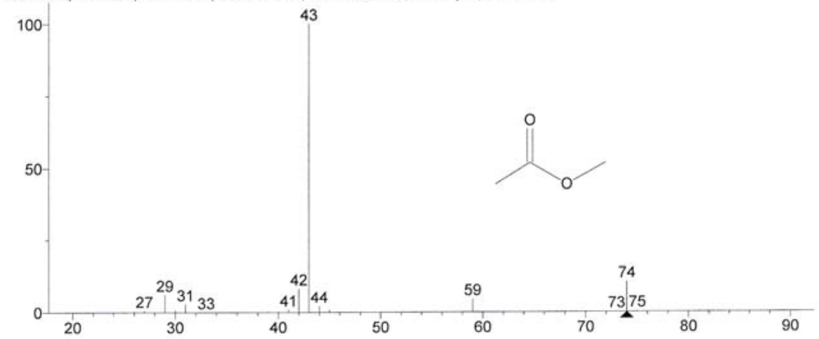

(replib) Acetic acid, methyl ester

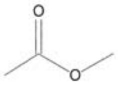

Name: Acetic acid, methyl ester
Formula: $C_3H_6O_2$
MW: 74 CAS#: 79-20-9 NIST#: 154747 ID#: 2573 DB: replib
Other DBs: Fine, TSCA, RTECS, HODOC, NIH, EINECS, IRDB
Contributor: Chemical Concepts
10 largest peaks:
43 999 | 74 105 | 42 82 | 29 62 | 59 46 | 31 29 | 44 22 | 41 9 | 30 8 | 45 8 |
Synonyms:
1.Methyl acetate
2.Devoton
3.Tereton
4.CH3COOCH3
5.Methyl ethanoate
6.Acetate de methyle
7.Methyl acetic ester
8.Methylacetaat

Page 1 of 2

Figure 36:GC-MS of the crude of reaction obtained in conditions described in Table 2, entry 4 (part 3)

```
File       :C:\MSDCHEM\1\DATA\OCHOA\Snapshot\ADRIAN73.d
Operator   : D. CHAVEZ
Acquired   : 12 Aug 2014  14:19     using AcqMethod VOLATILESPESADOS.M
Instrument :    Numero 1
Sample Name: DET. DE ACETATO DE ETILO
Misc Info  :
Vial Number: 4
```

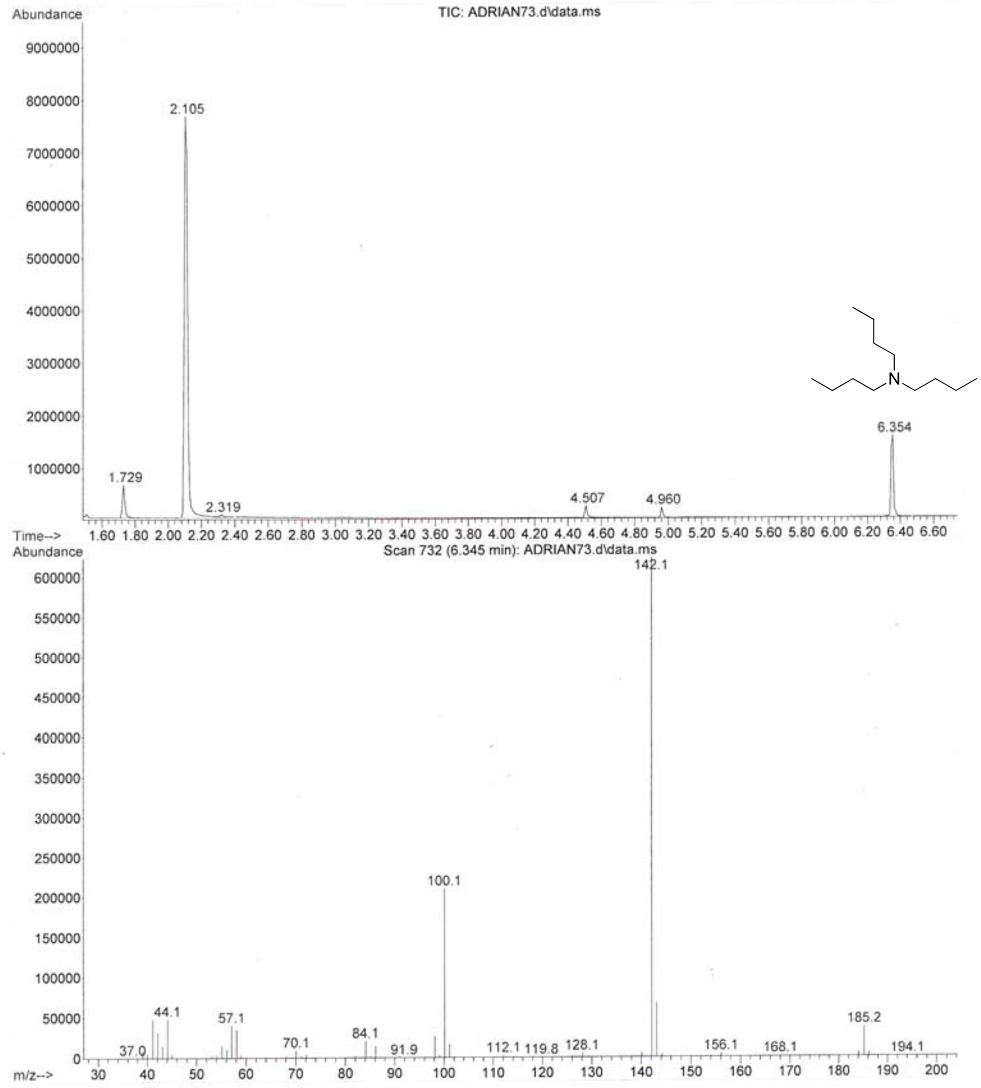

Figure 37:GC-MS of the crude of reaction obtained in conditions described in Table 2, entry 4 (part 4)

Hit 1 : Tributylamine
C12H27N; MF: 933; RMF: 935; Prob 82.8%; CAS: 102-82-9; Lib: mainlib; ID: 104480.

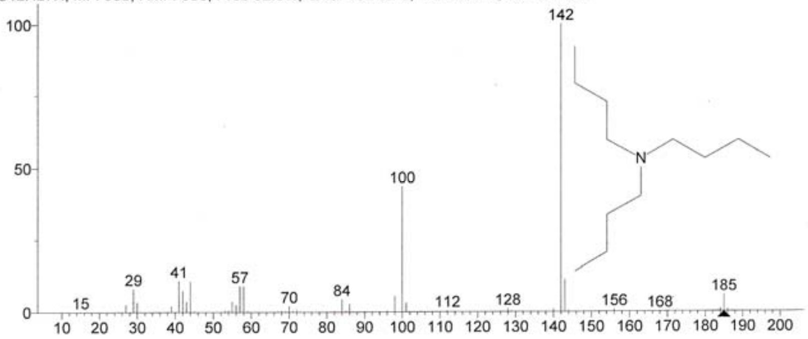

(mainlib) Tributylamine

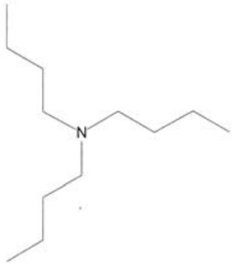

Name: Tributylamine
Formula: $C_{12}H_{27}N$
MW: 185 CAS#: 102-82-9 NIST#: 291265 ID#: 104480 DB: mainlib
Other DBs: Fine, TSCA, RTECS, HODOC, EINECS, IRDB
Contributor: NIST Mass Spectrometry Data Center, 1998.
10 largest peaks:
142 999 | 100 435 | 143 112 | 41 110 | 44 107 | 57 91 | 58 90 | 29 82 | 42 75 | 185 58 |
Synonyms:
1.1-Butanamine, N,N-dibutyl-
2.Tri-n-butylamine
3.Tris[N-butylamine]
4.(n-C4H9)3N
5.Tributilamina
6.UN 2542
7.N,N-Dibutyl-1-butanamine #

Page 1 of 1

Figure 38:GC-MS of the crude of reaction obtained in conditions described in Table 2, entry 4 (part 5)

```
File       :C:\msdchem\1\data\OCHOA\ADRIAN73.D
Operator   : D. CHAVEZ
Acquired   : 12 Aug 2014  14:19     using AcqMethod VOLATILESPESADOS.M
Instrument :    Numero 1
Sample Name: DET. DE ACETATO DE ETILO
Misc Info  :
Vial Number: 4
```

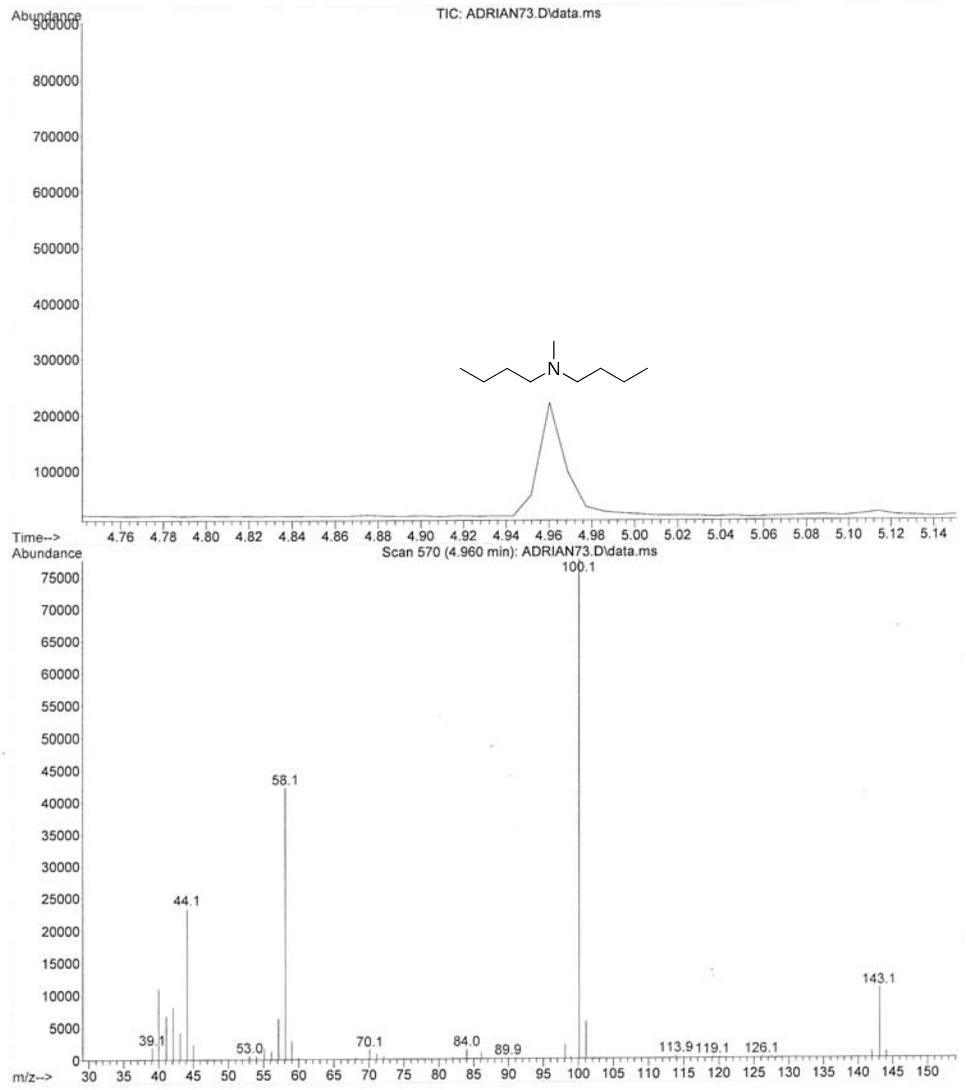

Figure 39:GC-MS of the crude of reaction obtained in conditions described in Table 2, entry 4 (part 6)

Hit 1 : 1-Butanamine, N-butyl-N-methyl-
C9H21N; MF: 836; RMF: 866; Prob 67.0%; CAS: 3405-45-6; Lib: mainlib; ID: 62561.

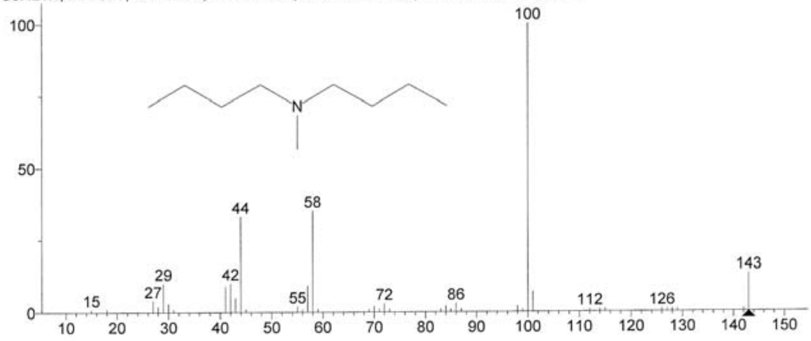

(mainlib) 1-Butanamine, N-butyl-N-methyl-

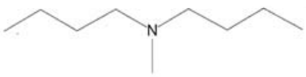

Name: 1-Butanamine, N-butyl-N-methyl-
Formula: $C_9H_{21}N$
MW: 143 CAS#: 3405-45-6 NIST#: 46649 ID#: 62561 DB: mainlib
Other DBs: Fine, TSCA, RTECS, EINECS
Contributor: CARL DJERASSI DEPT OF CHEM STANFORD UNIV STANFORD CALIF 94305
10 largest peaks:
100 999 | 58 350 | 44 330 | 143 130 | 29 100 | 42 100 | 41 90 | 57 90 | 101 70 | 43 50 |
Synonyms:
1.Dibutylamine, N-methyl-
2.Methanamine, N,N-dibutyl-
3.Methyldibutylamine
4.N-Methyldibutylamine
5.N,N-Dibutylmethylamine
6.di-n-Butylmethylamine
7.Methyl di-n-butylamine
8.N,N-di-n-Butylmethylamine

Page 1 of 2

Figure 40:GC-MS of the crude of reaction obtained in conditions described in Table 2, entry 4 (part 7)

File   :C:\MSDCHEM\1\DATA\OCHOA\Snapshot\ADRIAN74.d
Operator   : D. CHAVEZ
Acquired   : 12 Aug 2014 14:58   using AcqMethod VOLATILESPESADOS.M
Instrument :   Numero 1
Sample Name: REACCION 14
Misc Info   : DET. DE ACETATO DE ETILO
Vial Number: 6

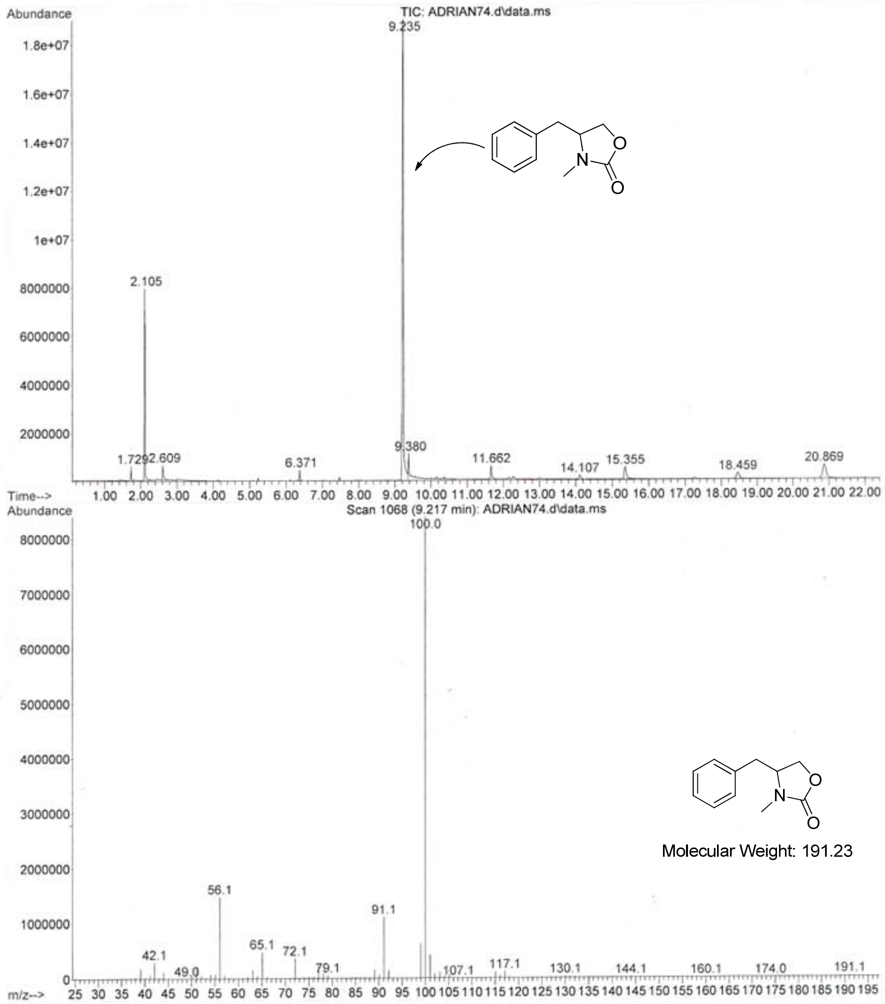

Figure 41:GC-MS of the crude of reaction obtained in conditions described in Table 2, entry 9 (part 1)

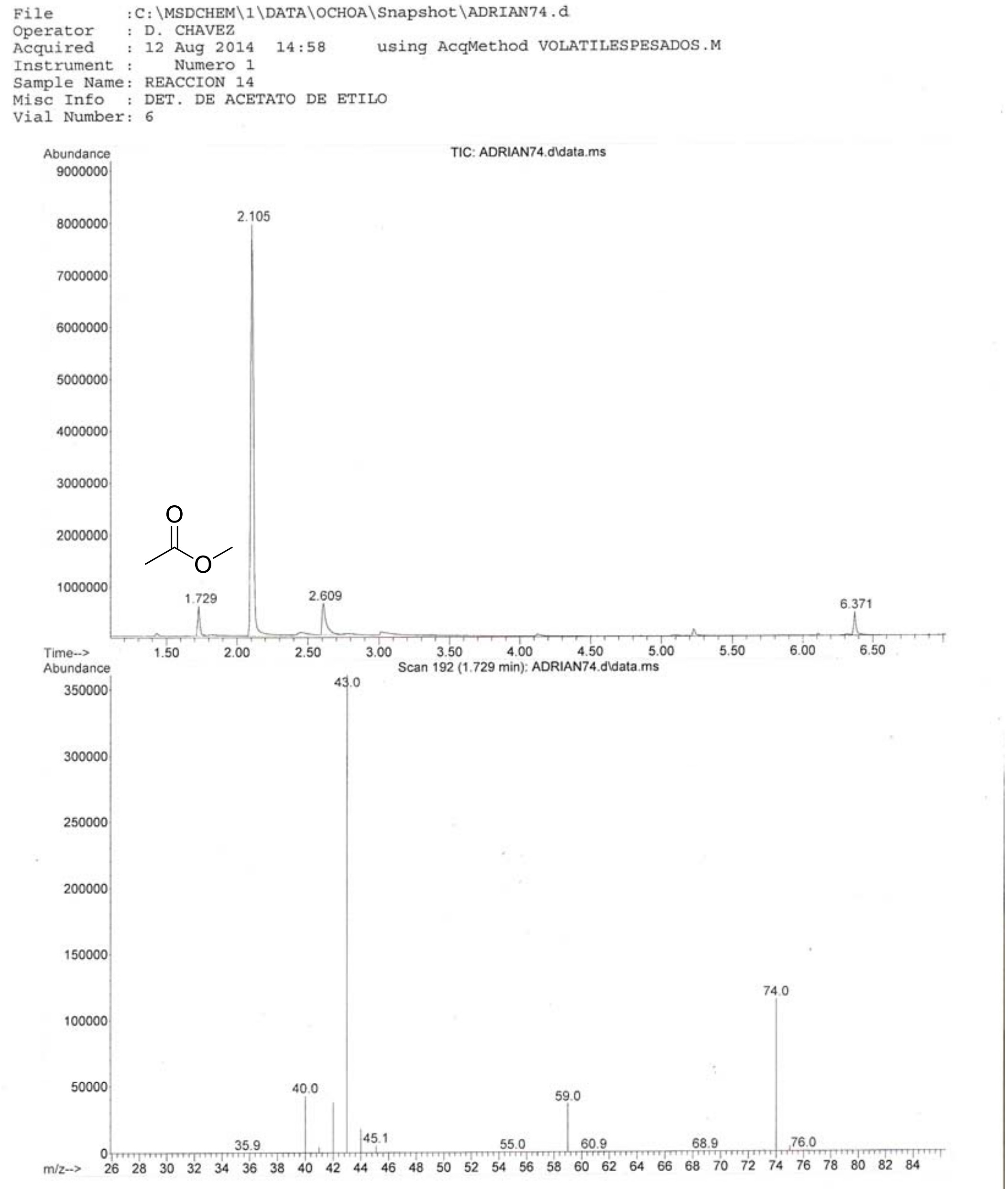

Figure 42:GC-MS of the crude of reaction obtained in conditions described in Table 2, entry 9 (part 2)

Hit 1 : Acetic acid, methyl ester
C3H6O2; MF: 907; RMF: 937; Prob 86.3%; CAS: 79-20-9; Lib: replib; ID: 2569.

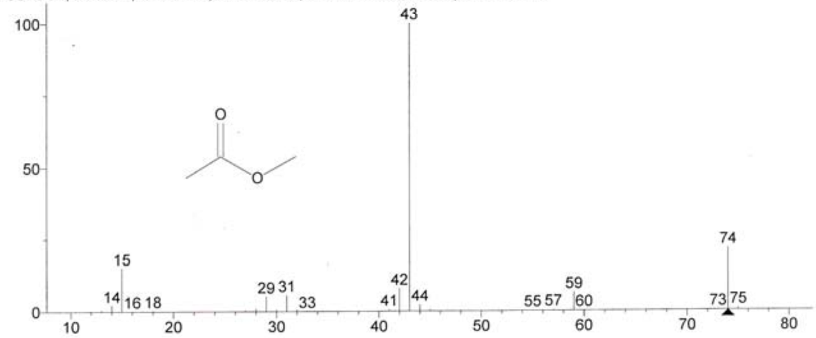

(replib) Acetic acid, methyl ester

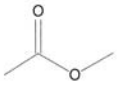

Name: Acetic acid, methyl ester
Formula: $C_3H_6O_2$
MW: 74 CAS#: 79-20-9 NIST#: 229449 ID#: 2569 DB: replib
Other DBs: Fine, TSCA, RTECS, HODOC, NIH, EINECS, IRDB
Contributor: Japan AIST/NIMC Database- Spectrum MS-NW- 27
10 largest peaks:
43 999 | 74 214 | 15 150 | 42 79 | 59 64 | 31 57 | 29 52 | 44 23 | 14 21 | 28 10 |
Synonyms:
1.Methyl acetate
2.Devoton
3.Tereton
4.CH3COOCH3
5.Methyl ethanoate
6.Acetate de methyle
7.Methyl acetic ester
8.Methylacetaat

Page 1 of 2

Figure 43:GC-MS of the crude of reaction obtained in conditions described in Table 2, entry 9 (part 3)

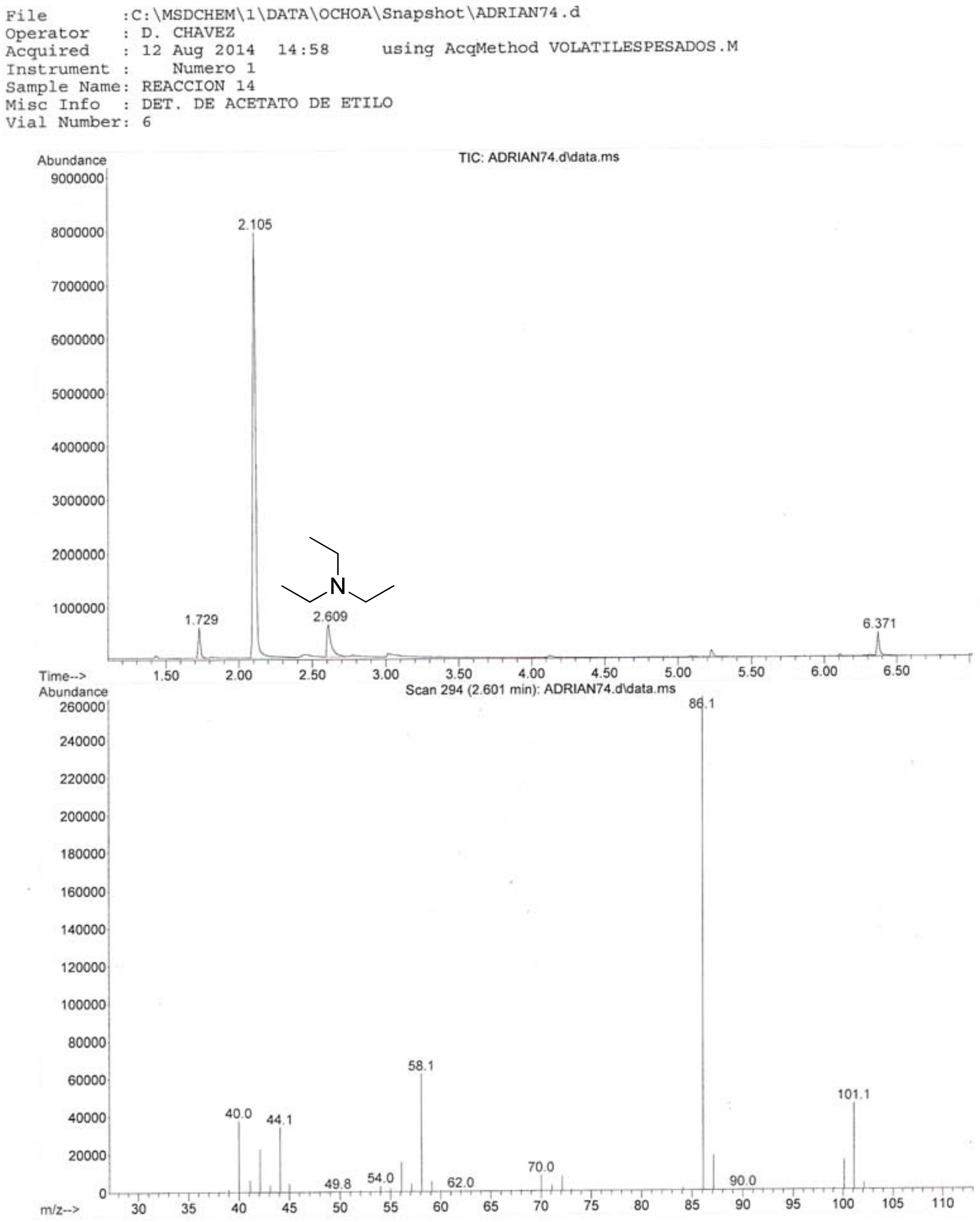

Figure 44:GC-MS of the crude of reaction obtained in conditions described in Table 2, entry 9 (part 4)

Hit 1 : Triethylamine
C6H15N; MF: 907; RMF: 908; Prob 82.4%; CAS: 121-44-8; Lib: mainlib; ID: 47867.

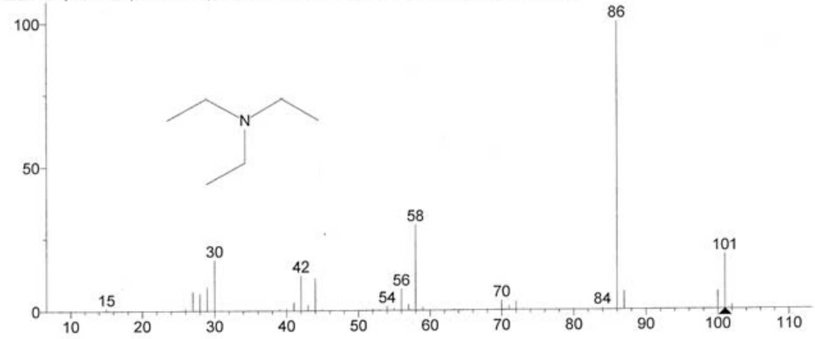

(mainlib) Triethylamine

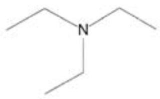

Name: Triethylamine
Formula: $C_6H_{15}N$
MW: 101 CAS#: 121-44-8 NIST#: 291545 ID#: 47867 DB: mainlib
Other DBs: Fine, TSCA, RTECS, EPA, HODOC, EINECS, IRDB
Contributor: NIST Mass Spectrometry Data Center, 1998.
10 largest peaks:
86 999 | 58 296 | 101 188 | 30 174 | 42 121 | 44 113 | 29 82 | 56 75 | 27 66 | 87 63 |
Synonyms:
1.Ethanamine, N,N-diethyl-
2.(C2H5)3N
3.(Diethylamino)ethane
4.N,N-Diethylethanamine
5.TEN
6.Triaethylamin
7.Trietilamina
8.UN 1296

Page 1 of 2

Figure 45:GC-MS of the crude of reaction obtained in conditions described in Table 2, entry 9 (part 5)

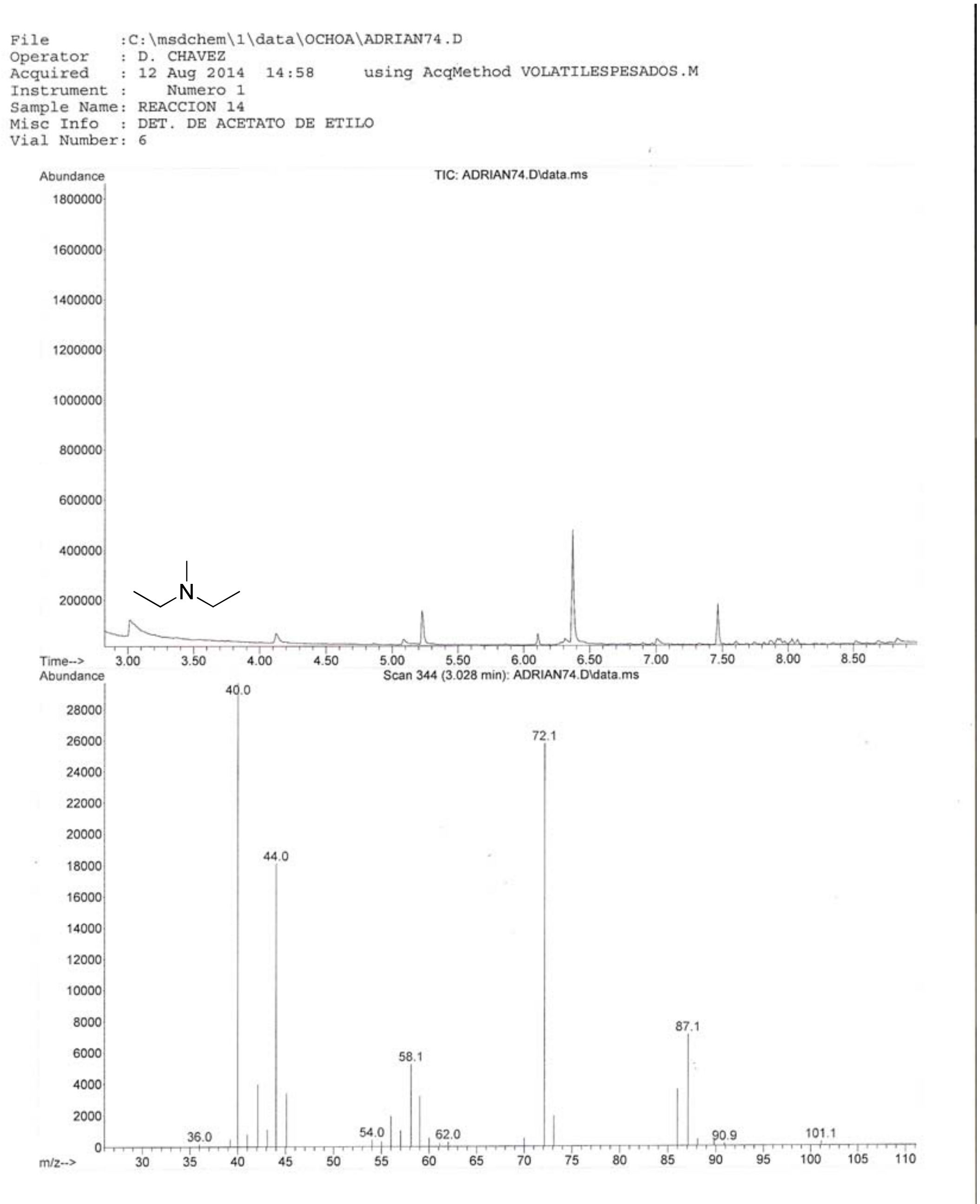

Figure 46:GC-MS of the crude of reaction obtained in conditions described in Table 2, entry 9 (part 6)

Hit 1 : Ethanamine, N-ethyl-N-methyl-
C5H13N; MF: 760; RMF: 770; Prob 50.9%; CAS: 616-39-7; Lib: mainlib; ID: 34134.

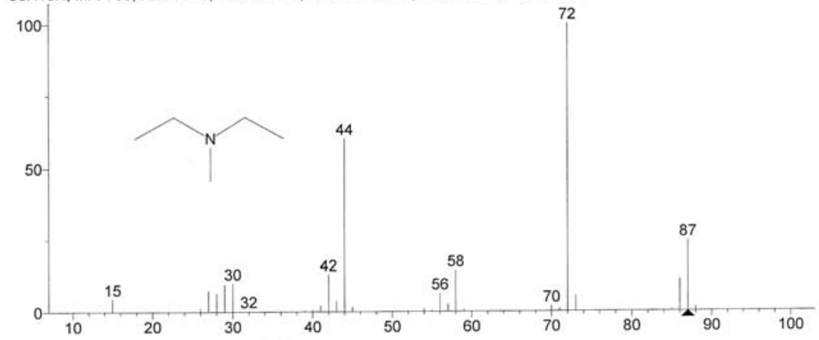

(mainlib) Ethanamine, N-ethyl-N-methyl-

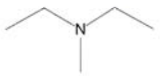

Name: Ethanamine, N-ethyl-N-methyl-
Formula: $C_5H_{13}N$
MW: 87 CAS#: 616-39-7 NIST#: 233768 ID#: 34134 DB: mainlib
Other DBs: Fine, TSCA, HODOC, EINECS
Contributor: Japan AIST/NIMC Database- Spectrum MS-NW-2121
10 largest peaks:
72 999 | 44 602 | 87 244 | 58 143 | 42 129 | 86 110 | 30 100 | 29 95 | 27 72 | 56 64 |
Synonyms:
1.Diethylamine, N-methyl-
2.Diethylmethylamine
3.Methyldiethylamine
4.N-Methyldiethylamine
5.N,N-Diethylmethylamine
6.(C2H5)2(CH3)N
7.N-Ethyl-N-methyl-ethanamine

Page 1 of 1

Figure 47:GC-MS of the crude of reaction obtained in conditions described in Table 2, entry 9 (part 7)

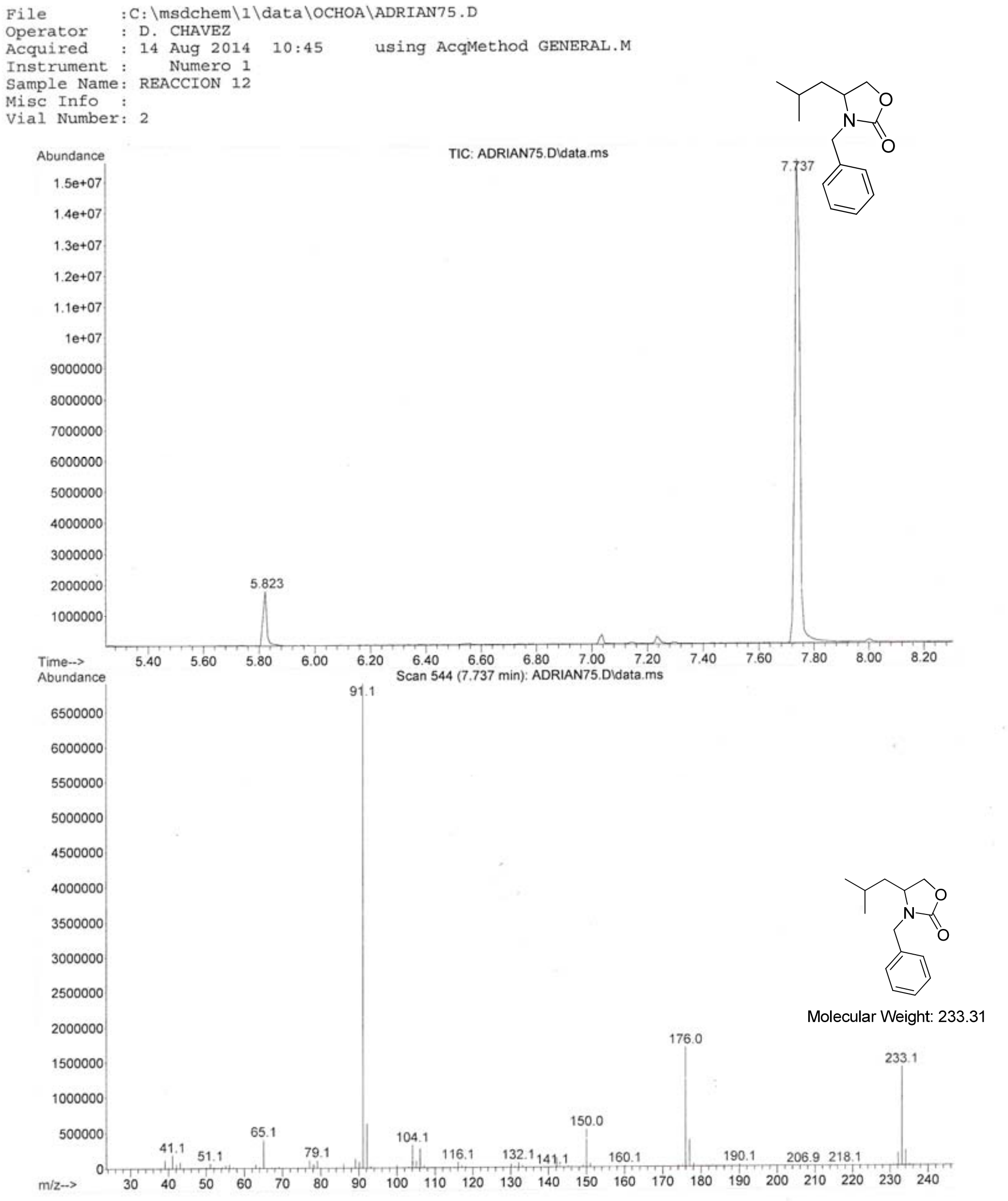

Figure 48:GC-MS of the crude of reaction obtained in conditions described in Table 2, entry 10 (part 1)

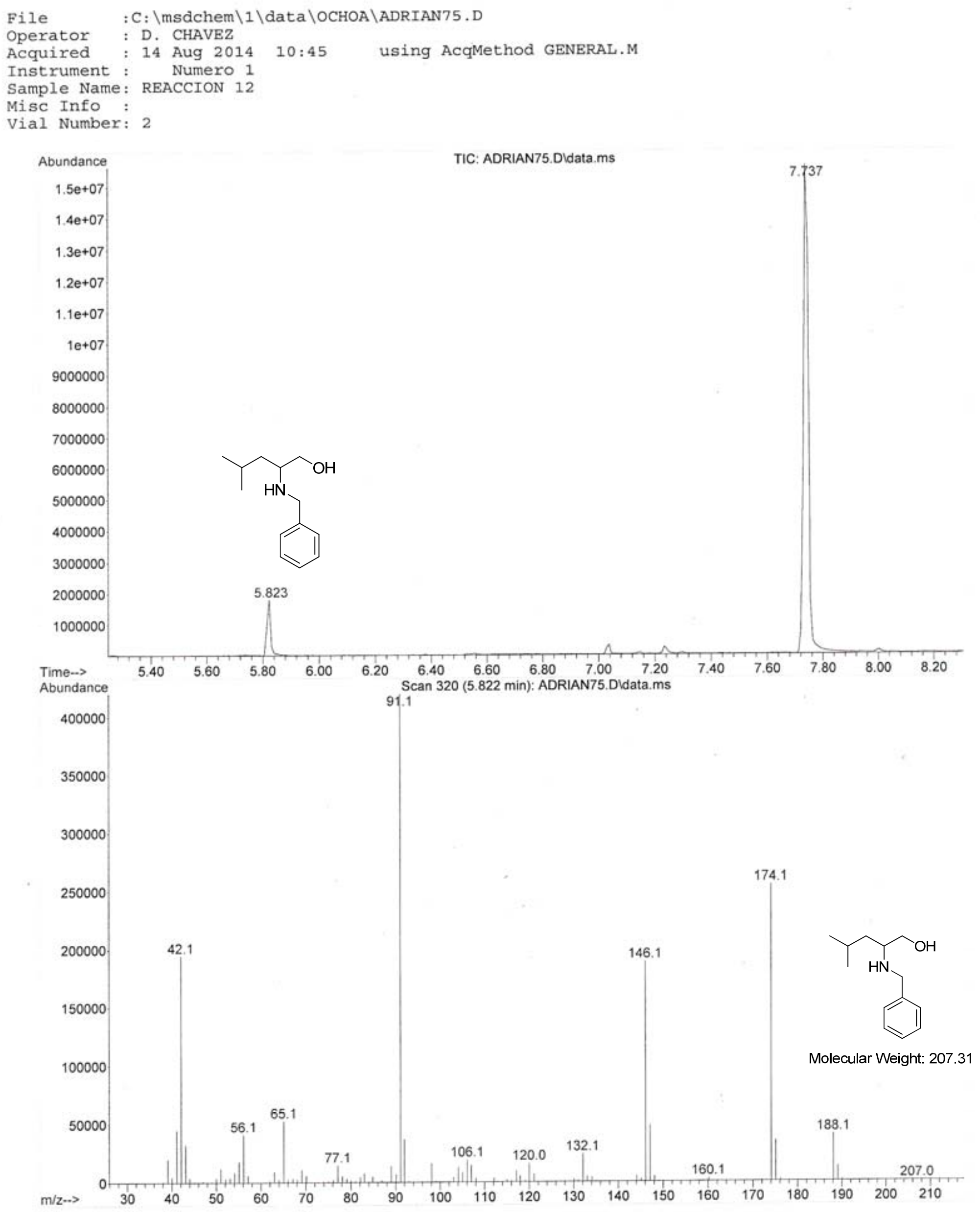

Figure 49:GC-MS of the crude of reaction obtained in conditions described in Table 2, entry 10 (part 2)
